# The Oxidative Metabolism of Fossil Hydrocarbons and Sulfide Minerals by the Lithobiontic Microbial Community Inhabiting Deep Subterrestrial Kupferschiefer Black Shale

**DOI:** 10.3389/fmicb.2018.00972

**Published:** 2018-05-15

**Authors:** Agnieszka Włodarczyk, Maciej Lirski, Anna Fogtman, Marta Koblowska, Grzegorz Bidziński, Renata Matlakowska

**Affiliations:** ^1^Laboratory of Environmental Pollution Analysis, Faculty of Biology, University of Warsaw, Warsaw, Poland; ^2^Institute of Biochemistry and Biophysics, Polish Academy of Sciences, Warsaw, Poland; ^3^Laboratory of Systems Biology, Faculty of Biology, University of Warsaw, Warsaw, Poland; ^4^KGHM Polska Miedź S.A., Lubin Mine Division, Lubin, Poland

**Keywords:** black shale, kerogen, fossil hydrocarbons, sulfide minerals, metagenome, metaproteome

## Abstract

Black shales are one of the largest reservoirs of fossil organic carbon and inorganic reduced sulfur on Earth. It is assumed that microorganisms play an important role in the transformations of these sedimentary rocks and contribute to the return of organic carbon and inorganic sulfur to the global geochemical cycles. An outcrop of deep subterrestrial ~256-million-year-old Kupferschiefer black shale was studied to define the metabolic processes of the deep biosphere important in transformations of organic carbon and inorganic reduced sulfur compounds. This outcrop was created during mining activity 12 years ago and since then it has been exposed to the activity of oxygen and microorganisms. The microbial processes were described based on metagenome and metaproteome studies as well as on the geochemistry of the rock. The microorganisms inhabiting the subterrestrial black shale were dominated by bacterial genera such as *Pseudomonas, Limnobacter, Yonghaparkia, Thiobacillus, Bradyrhizobium*, and *Sulfuricaulis*. This study on black shale was the first to detect archaea and fungi, represented by *Nitrososphaera* and *Aspergillus* genera, respectively. The enzymatic oxidation of fossil aliphatic and aromatic hydrocarbons was mediated mostly by chemoorganotrophic bacteria, but also by archaea and fungi. The dissimilative enzymatic oxidation of primary reduced sulfur compounds was performed by chemolithotrophic bacteria. The geochemical consequences of microbial activity were the oxidation and dehydrogenation of kerogen, as well as oxidation of sulfide minerals.

## Introduction

Sedimentary rocks are one of the largest reservoirs of fossil organic carbon and inorganic reduced sulfur compounds on Earth. The total content of C_organic_ and S_sulfidic_ in these rocks is estimated at ~ 1.5 × 10^22^ g and ~ 39.5 × 10^20^ g, respectively (Berner, 1989; Rickard, 2012). The richest reservoir of C_organic_ and S_sulfidic_ are shales, the most abundant ancient sedimentary rocks on Earth, in which they may constitute up to 20 and 0.4 wt%, respectively (Tourtelot, 1979; Rickard, 2012). The oxidation of these sedimentary rocks is of great significance, because it contributes to the return of organic carbon and inorganic sulfur trapped in the form of refractory fossil organic matter and insoluble sulfides to the global biogeochemical cycles on the Earth's near surface and subsurface.

Microbial processes of sulfide mineral dissolution based on chemolithotrophic oxidation have been known since the 1950s (Bryner et al., 1954), and the heterotrophic processes of assimilation of the fossil organic carbon trapped in black shales were discovered at the beginning of the Twenty first century (Petsch et al., 2001). These processes have always been studied and described independently; however, in the case of sedimentary rocks such as kerogen- and sulfide mineral-bearing black shales, these processes can coexist. There is still very little knowledge about such simultaneous processes and many questions remain unanswered. The taxonomy, physiology and metabolism of microbial communities inhabiting black shales are some of the most important and as yet understudied areas of research. The biochemical pathways of microbial processes cause oxidation, and the way these processes affect the geochemistry of oxidized rocks and waters are largely unknown. In addition, the diversity of sedimentary rocks, not only in terms of their origin and age, location, geochemical composition but also their physicochemical properties, justifies further research toward a better understanding of these unique geomicrobial processes.

In our field studies, we examined the lithobiontic microbial community (LMC) inhabiting the subterrestrial Kupferschiefer black shale at a depth of 777 meters and its role in the oxidative weathering of fossil hydrocarbons and sulfide minerals. Kupferschiefer is a neutral Lopingian sedimentary rock of marine origin, containing large amounts of organic carbon (up to 16 wt%) dominated by hydrocarbons (kerogen type II), and also a high content of sulfur (~2.9 wt%), dominated by reduced sulfur compounds [bornite (Cu_5_FeS_4_), chalcocite (Cu_2_S), digenite (Cu_9_S_5_), chalcopyrite (CuFeS_2_), pyrite (FeS_2_), sphalerite (ZnS), galena (PbS), covellite (CuS), and minerals belonging to the tennantite-tetrahedrite series; (Cu, Fe)_12_As_4_S_13_ – (Cu, Fe)_12_Sb_4_S_13_] (Sawłowicz, 1989; Speczik, 1994; Oszczepalski, 1999).

**Figure 1:**
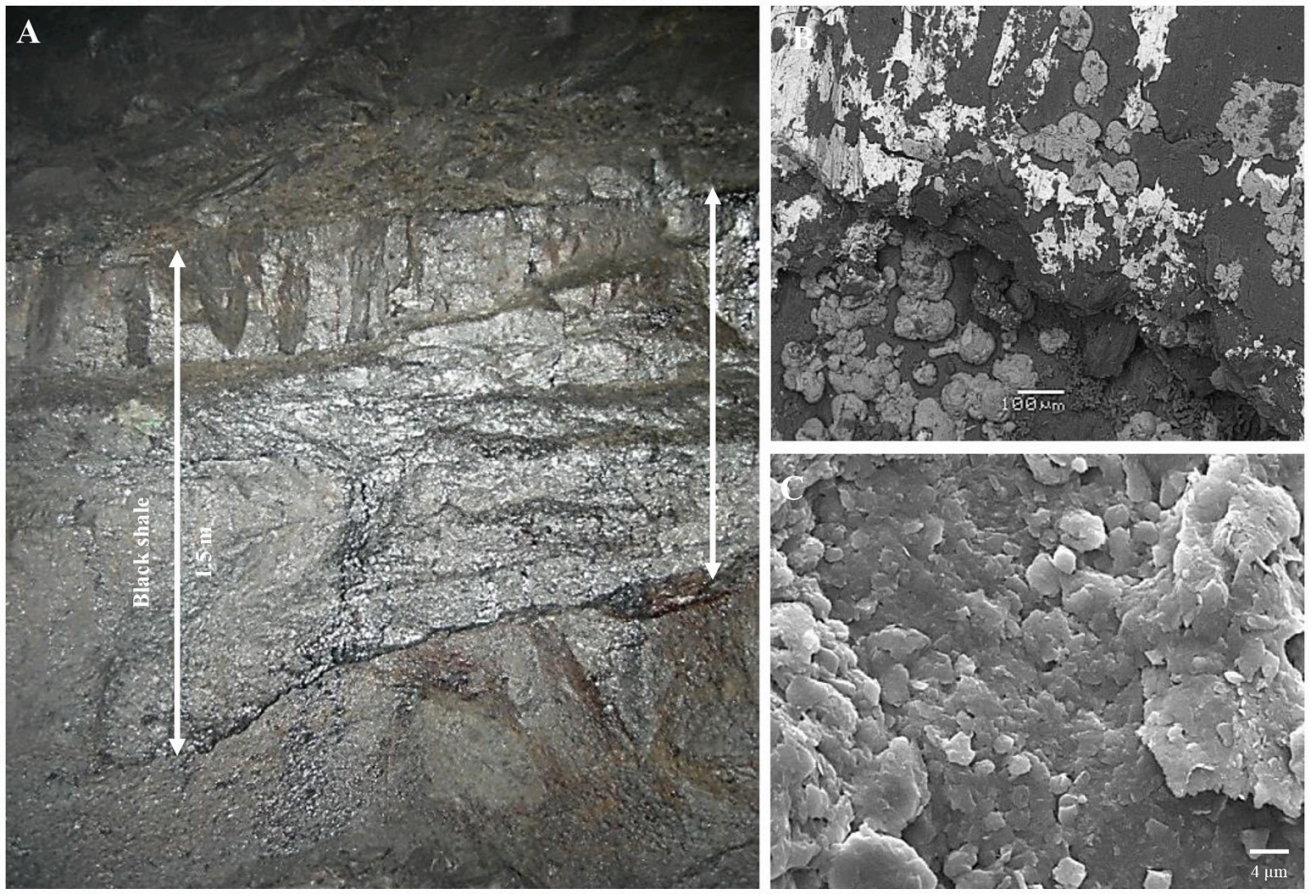
Outcrop of subterrestrial black shale - macroscopic **(A)** and scanning electron microscope **(B,C)** images.

In the current studies, the profile of the black shale which had been exposed 12 years ago during mining activity in the area of Lubin copper mine was analyzed (Figure 1). In a subterrestrial mine the processes of the aerobic oxidation of fossil hydrocarbons and sulfide minerals occurring in the Kupferschiefer black shale may be intensive and may be proceed with the participation of either abiotic factors (oxygen, water) or microorganisms inhabiting the black shale. In previous laboratory studies, we described the long-term oxidation of Kupferschiefer kerogen by an indigenous bacterial consortium (Stasiuk et al., 2017). We reported that the bacterial oxidation of fossil hydrocarbons may be one of the processes responsible for kerogen weathering. However, the research so far had been limited to only a bacterial metaproteome analysis and identification of oxidized organic compounds. Metagenomic tools had not been utilized, and the participation of archaea and fungi in these processes had also not been recognized. Similarly, our knowledge about the influence of microorganisms on sulfide minerals deposited in Kupferschiefer black shale is even more limited. The dissolution of these minerals has been observed in laboratory experiments, although this was caused by chemical compounds—intermediates and products of fossil organic matter degradation by heterotrophic bacteria (Włodarczyk et al., 2016). So far, neither bacterial nor archaeal nor fungal enzymatic processes of oxidation of inorganic sulfur compounds have been studied.

The aim of our study was to define the microbial metabolic processes of fossil hydrocarbons and sulfide mineral oxidation at the molecular level of resolution. The taxonomic diversity of LMC inhabiting the black shale, including bacteria, archaea and fungi, was identified. Next, protein-encoding genes (PEGs) and enzymes responsible for the oxidative metabolism of hydrocarbons and sulfide minerals were indicated in the metagenome and metaproteome of LMC, respectively. The microorganisms and the metabolic processes crucial in the oxidation of these two components of black shale were indicated. Finally, the geochemical consequences of microbial activity were described.

## Materials and methods

### Site and samples description

Three samples of black shale were collected from a 12-year-old outcrop at a depth of 777 m in Lubin copper mine (Figure 1). The mine is located in Lubinsko-Głogowski Copper District (51.434015, 16.156069) (SW Poland). The samples were collected aseptically from outcrops (to a depth of 10 cm) and kept at −80 and −4°C until processing in the lab. The unweathered black shale originating from a freshly exposed outcrop (2 days) in Lubin mine has previously been described (Stasiuk et al., 2017).

### Microscopic observations

The samples were fixed in paraformaldehyde vapor, gold coated and observed under a scanning electron microscope (Leo 1430VP, LEO Electron Microscopy Inc., USA).

### DNA isolation

DNA was isolated from 100 g of sample according to a modified procedure of Zhou et al. (1996). The samples were resuspended in 100 ml of DNA extraction buffer (0.1 M Na_2_EDTA, 0.1 M Tris-HCl, 0.1 M Na_2_HPO_4_, 1.5 M NaCl, 1% CTAB (hexa-decylmethylammonium bromide), pH 8.0) containing proteinase K and lysozyme (750 μl; 10 mg/ml) and then were incubated overnight at 37°C with horizontal shaking. Following the addition of 20% SDS, each sample was incubated for 4 h at 65°C and centrifuged (6,000 × g, 10 min). Harvested supernatant was mixed with an equal volume of chloroform/isoamyl alcohol mixture (24:1; v/v) and centrifuged (6,000 × g, 10 min). The aqueous phase was collected and precipitated overnight with 0.6 volume of isopropanol at room temperature. Next, the sample was centrifuged (16,000 × g, 20 min, 4°C), and the pellet was washed with cold 70% ethanol and allowed to dry. Isolated DNA was resuspended in 50 μl of sterile deionized water and stored at −80°C.

### DNA sequencing and analysis

Isolated DNA was used to prepare barcoded library with Ion Xpress™ Plus Fragment Library Kit (Thermo Fisher) according to manufacturer's manual. Library was subjected to clonal amplification on Ion One Touch 2 system (Thermo Fisher) using Ion PI™ Template OT2 200 Kit v2 (Thermo Fisher) and sequenced on Ion Proton sequencer using Ion PI™ Sequencing 200 Kit v2 (Thermo Fisher) according to manufacturer's manual. Reads were demultiplexed with Torrent Suite software. Fastq file was quality trimmed and filtered for low complexity reads, reads shorter than 34 nucleotides and duplicate reads using PRINSEQ (Schmieder and Edwards, 2011). Filtered reads were aligned to National Center for Biotechnology Information Non-Redundant (NCBI nr) protein database, using DIAMOND-0.8.18 Basic Local Alignment Search Tool X (BLASTX) Alignment Mode (Buchfink et al., 2015) with “more sensitive” option and VTML200 scoring matrix and following parameters: λ = 0.318, K = 0.13, gap open penalty = 15 and gap extend penalty = 1.5. Statistical parameters for VTML200 scoring matrix were computed using fastx36 script from FASTA-36.3.8d suite (Pearson 1988-2014). Resulting alignment file was “meganized” using script supplied with MEGAN 6.6.1 (Huson et al., 2007). Taxonomical analyses were performed in MEGAN using Lowest Common Ancestor (LCA) algorithm on alignments from DIAMOND. To minimize false positive hits a cutoff of minimum bitscore 60 and at least 207 reads to report a taxon was selected. Cutoff parameters were defined by thorough analysis of rarefaction curves generated with different filtering parameters. Other LCA parameters were set to default values. Krona charts were generated with data from tab-separated “read name—taxon id” file exported from MEGAN, using ktImportTaxonomy function from KronaTools (Ondov et al., 2011) with default settings. Coverage of selected proteins was calculated using custom pipeline consisting of bash and python scripts, implementing GNU Parallel (Tange, 2011) and ETE3 (Huerta-Cepas et al., 2016). Briefly, for each read, hits with minimum bitscore 60 were used, and the best hits were selected based on other BLAST parameters. Coverage was calculated for unique best scoring hits. Next, reads having multiple best scoring hits were divided and distributed among references present in unique hits based on weights calculated from unique read coverage of those references. Non unique reads (URs), which had no hits among references present in unique hits, were divided based on coverage of species they derived from. Non URs aligning to species not present in unique hits were dropped. Summed coverage for selected proteins was calculated based on protein descriptions from NCBI nr database. Results were manually supervised and corrected until no erroneous assignments were found.

### Isolation of proteins

Proteins were isolated from 100 g of sample according to the modified procedure of Ram et al. (2005). Each sample was resuspended in 120 ml of 20 mM Tris-HCl, pH 8, shaken for 3 min, and sonicated on ice−10 × 1 min, with 1-min pauses (Sonics Vibracell; LABOPLUS, ModelCV18head). One hundred milliliters of 0.4 M Na_2_CO_3_ (pH 11) were added to the suspension of the lysed cells and the sample was centrifuged to remove the unlysed cells and cell membrane fragments (6,000 × g, 20 min, 4°C) and filtered (filter with 0.22 μm pore size). Proteins were precipitated from the solution with trichloroacetic acid (TCA) [1:10(v/v)]. The precipitation was performed overnight at 4°C, and then the sample was centrifuged (20,000 × g, 10 min, 4°C). The aqueous phase was discarded and the protein pellet was resuspended in 0.5 ml of methanol precooled to 4°C, and then centrifuged (20,000 × g, 10 min, 4°C). The resulting precipitate was dried. The proteins were stored at −80°C. The analysis was performed in triplicate.

### Identification of proteins

The identification of proteins was performed by LC-MS-MS/MS (liquid chromatography coupled to tandem mass spectrometry) using a Nano-Acquity (Waters) LC system and Orbitrap Velos mass spectrometer (Thermo Electron Corp., San Jose, CA). Prior to the analysis, proteins were subjected to a standard “in-solution digestion” procedure during which proteins were reduced with 50 mM Tris(2-carboxyethyl)phosphine (for 60 min at 60°C), alkylated with 200 mM S-methyl methanethiosulfonate (45 min at room temperature) and digested overnight with trypsin (Sequencing Grade Modified Trypsin; Promega V5111). The peptide mixture was applied to an RP-18 precolumn (nanoACQUITY Symmetry® C18; Waters 186003514) using water containing 0.1% trifluoroacetic acid as the mobile phase and then transferred to a nano-HPLC RP-18 column (nanoACQUITY BEH C18; Waters 186003545) using an acetonitrile (ACN) gradient (5–35% ACN in 180 min) in the presence of 0.05% formic acid with a flow rate of 250 μl/min. The column outlet was directly coupled to the ion source of the spectrometer working in the regime of data dependent MS to MS/MS switch. A blank run ensuring a lack of cross-contamination from previous samples preceded each analysis. Acquired raw data were processed by Mascot Distiller followed by Mascot Search (Matrix Science, London, UK, on-site license) against the NCBI nr database. Peptides with a Mascot Score exceeding a threshold value corresponding to < 5% expectation value, calculated by Mascot procedure, were considered to have been positively identified.

### Pyrolytic analysis

The pyrolytic analysis of each sample was performed using the Rock-Eval technique. This involved thermal decomposition of rock samples (100 mg) in two cycles, pyrolytic and oxidizing, respectively (Lafargue et al., 1998). In the pyrolysis step, the sample was heated to 300°C in a nitrogen atmosphere to release the S1 fraction (mg HC/g rock) made of volatile compounds. This stage was followed by heating the sample to 650°C to release the S2 (mg HC/g rock), S3CO_2_ (mg CO_2_/g rock), and S3CO (mg CO/g rock) fractions. S1 and S2 were measured with a flame ionization detector, while S3 with infrared spectroscopy. The S2 fraction represented the products of kerogen cracking and the S3 fraction derived from oxygen-containing moieties (e.g., aldehydes and alcohols). In the second cycle, the sample was heated to 850°C in an oxygen atmosphere to release carbon monoxide and carbon dioxide from the residual and unproductive organic matter and mineral matter. The obtained results were converted into pyrolyzed organic carbon content (PC), residual organic carbon content (RC), and total organic carbon content (TOC). Other important parameters provided by the method are the hydrogen index (HI) and the oxygen index (OI), which allow the characterization of the type of organic matter and petroleum potential of the rock. The analysis was performed in triplicate.

### Extraction of organic compounds

The sample was dried at 60°C and powdered. Organic compounds were extracted from 20 g of sample using a mixture of dichloromethane/methanol (9:1; v/v) for 24 h with a Soxhlet extraction apparatus. The solvent was evaporated with an N_2_ stream and then the sample was derivatized with 0.5 ml of BSTFA:TMCS (N,O-bis(Trimethylsilyl)trifluoroacetamide: Trimethylchlorosilane), 99:1 (Supelco, USA), for 30 min at 70°C. A blank sample was prepared according to the same procedure. All analyses were performed in triplicate.

### Analysis of extractable organic compounds

The separation of organic compounds was performed using an Agilent 7890A Series Gas Chromatograph (GC) interfaced to an Agilent 5973c Network Mass Selective Detector and an Agilent 7683 Series Injector (Agilent Technologies, USA). A 5 μl sample was introduced (split by 0.3% SD) to an HP-5MS column (30 m × 0.25 mm I.D., 0.25 μm film thickness, Agilent Technologies, USA) using He as the carrier gas at 1 ml/min. The ion source was maintained at 250°C; the GC oven was programmed with a temperature gradient starting at 100°C (for 3 min) and this was gradually increased to 300°C (for 5 min) at 8°C/min. A mass spectrometry (MS) analysis was performed in electron-impact mode at an ionizing potential of 70 eV. Mass spectra were recorded from m/z 40 to 800 (0–30 min).

### Selection, identification and classification of organic compounds

The identification of organic compounds was performed with an Agilent Technologies Enhanced ChemStation (G1701EA ver. E.02.00.493) and The Wiley Registry of Mass Spectral Data (version 3.2, Copyright 1988–2000 by Palisade Corporation, 8th Edition with Structures, Copyright 2000 by John Wiley and Sons, Inc.) using a 3% cut-off threshold. The selected peaks representing organic compounds whose mass spectra indicated compliance with reference mass spectra equal to or higher than 80% were identified using the mass spectra library. The rest of the organic compounds representing lower compliance (50–79%) were assigned to the major classes of organic compounds based on the presence of characteristic and dominating fragmentation ions (aromatic hydrocarbons–m/z 65, 77, 78, 79; aliphatic hydrocarbons–m/z 43, 57, 71, 85, 99; alcohols–m/z 45, 59, 73, 87; aldehydes–m/z 44, 58, 72; carboxylic acids–m/z 43, 45, 57, 59, 60, 71, 73, 85, 87) (Silverstein et al., 2014).

### Sulfur speciation analysis

The total sulfur concentration was determined by infrared absorption spectroscopy using the Leco method (Leco Application Notes No ASTM E1915). The mean detection limit for this method is 0.02 wt%.

Sulfur speciation analysis was conducted using the methods described by Shimp et al. (1977) and Tuttle et al. (1986). Sulfates were extracted from the shale with hydrochloric acid (6 M) and precipitated from the obtained solution by barium chloride (10 wt%). The barium sulfate precipitate was filtered, calcined (925°C) and weighed and the content of sulfate sulfur was calculated from its mass. Pyrite included in the shale (after extraction of non-pyrite iron) was extracted with nitric acid (18 wt%). Iron was oxidized and its amount was determined using inductively coupled plasma-atomic emission spectrometry. The content of pyritic sulfur was calculated from the amount of oxidized iron(III). Sulfides were separated in an acid medium and absorbed to a solution of zinc acetate (1.0 wt%). Their amount was determined using the colorimetric method based on the reaction of N,N-dimethyl-p-phenylenediamine dihydrochloride in the presence of ferric chloride(III), which results in the formation of methylene blue. The organic sulfur content was calculated by subtracting the sulfate, pyrite and sulfite sulfur content from the total sulfur content. All analyses were performed in triplicate.

## Results and discussion

### Taxonomic diversity of LMC

**Figure 2:**
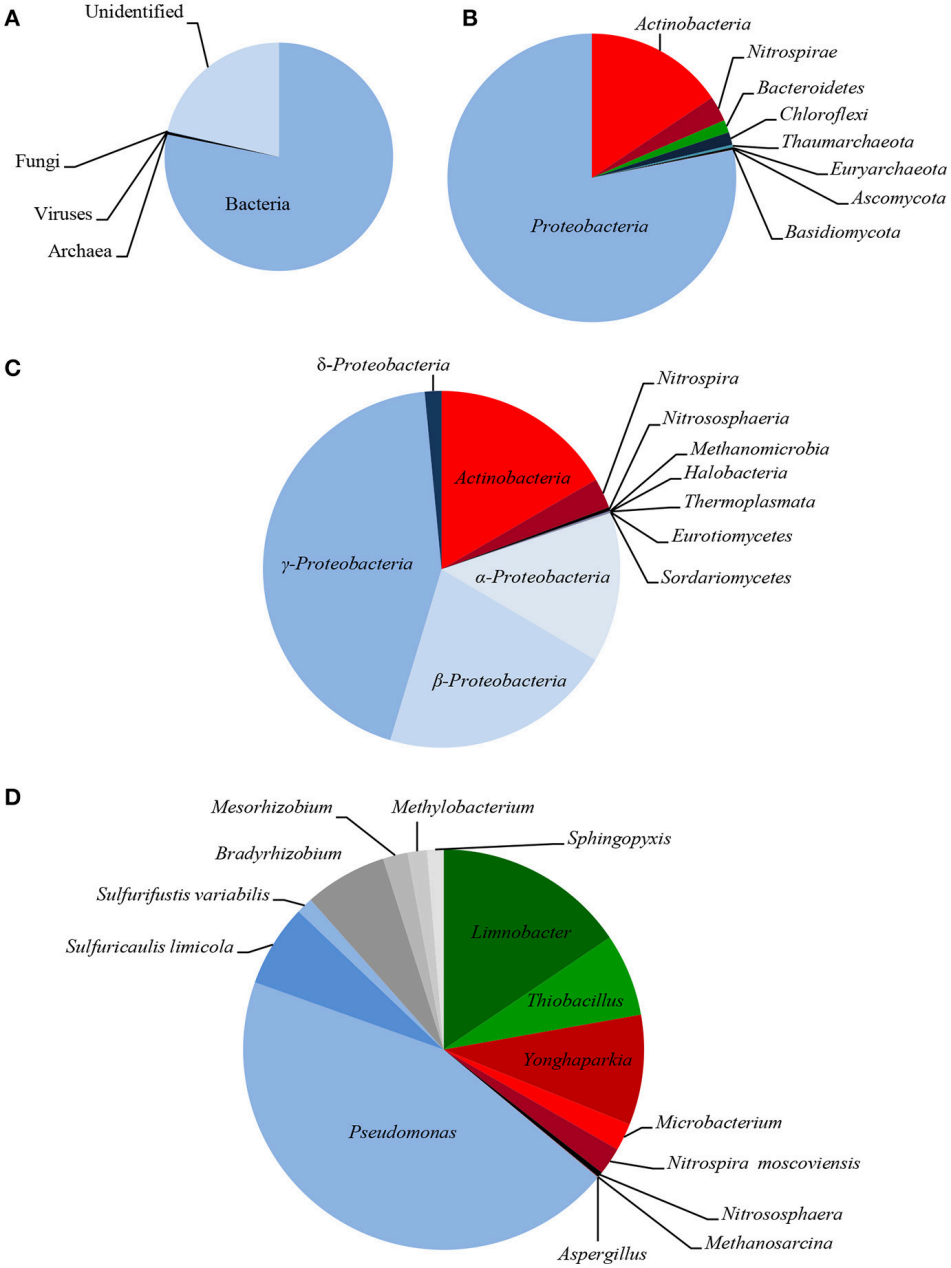
Taxonomic diversity of LMC: microbial superkingdoms (Bacteria, Archaea and Viruses) and kingdom (Fungi) **(A)**; phyla **(B)**; classes **(C)** and genera/species **(D)** (bacterial phyla/classes ≥ 1%, genera/species ≥ 0.5%; archaeal and fungal taxons ≥ 0.01%). The detailed taxonomic diversity of LMC is presented in Supplementary Presentation 1.

The taxonomic composition of LMC inhabiting the studied black shale based on the Ion Torrent^TM^ next-generation sequencing of the metagenome is presented in Figure 2, and in Supplementary Presentation 1. The rarefaction curve tended to approach the saturation plateau (Figure S1). LMC consisted of 23 bacterial phyla accounting for 78% of the total reads, as well as two phyla of archaea (0.4%), and two phyla of fungi (0.1%). Moreover, 0.02% of total reads belonged to viruses and the rest (21%) were unclassified (Figure 2A). Two phyla of bacteria dominated in LMC: *Proteobacteria* (55%) mostly represented by γ-*Proteobacteria* class (29%), and *Actinobacteria* (11%) (Figures 2B,C). *Nitrospirae* constituted 2% of the total reads, the next two bacterial phyla (*Bacteroidetes* and *Chloroflexi*) accounted for 1%, while the remaining 18 phyla represented < 1%. *Pseudomonadaceae* (20%) was the most abundant family and it was dominated by the *Pseudomonas stutzeri* group (14%) (Figure 2D). Six other bacterial families represented from 1.9 to 8% of the total reads: *Microbacteriaceae* (8%), *Burkholderiaceae* (7%), *Acidiferrobacteraceae* (4%), *Bradyrhizobiaceae* (3%), *Hydrogenophilaceae* (3%), and *Nitrospiraceae* (1.9%). Besides the genus *Pseudomonas, six* other genera and one species were dominant: *Limnobacter* (7%), *Yonghaparkia* (4%), *Thiobacillus* (3%), *Sulfuricaulis limicola* (3%), *Bradyrhizobium* (3%), *Nitrospira* (1.8%), and *Microbacterium* (1%) (Figure 2D). *Thaumarchaeota* (*Nitrososphaeria*) and *Euryarchaeota (Methanomicrobia, Halobacteria*, and *Thermoplasmata)* were the identified archaeal phyla, accounting for 0.2 and 0.1% of the total reads, respectively (Figures 2B,C). Two fungal phyla, *Ascomycota* (*Eurotiomycetes, Sordariomycetes*, and *Dothideomycetes*) and *Basidiomycota* (*Agaricomycetes*), constituting 0.09 and 0.02%, respectively, were also detected (Figures 2B,C).

So far, little is known about the microorganisms inhabiting black shales. The most commonly known cultivable bacteria isolated from terrestrial and subterrestrial black shales have been reported previously. Petsch et al. (2001, 2005) showed that microorganisms inhabiting Late Devonian New Albany Shale (USA) are represented by *Pseudomonas* spp., *Acinetobacter* spp., *Dechloromonas* spp., *Clostridium* spp., and *Comamonas* spp. Berlendis et al. (2014) detected *Ralstonia picketii* in Lower Autunian Autun black shale (France). Only heterotrophic bacteria such as *Pseudomonas* spp., *Bacillus* spp., *Microbacterium* spp. and *Acinetobacter* spp. as well as *Rhodotorulla mucilaginosa* yeast have previously been isolated from subterrestrial Kupferschiefer black shale (Matlakowska and Skłodowska, 2009; Rajpert et al., 2013). Only the studies by Li et al. (2014) into the bacterial biodiversity of the terrestrial exposed profile of the Lower Cambrian black shale from Chengkou County (China) have been based on an analysis of the sequence of the amplified 16S rRNA gene. They revealed that the shale was inhabited by bacteria belonging to 33 different groups and here *Proteobacteria, Actinobacteria*, and *Firmicutes* were dominant. The presented research is, therefore, the first report on metagenome-based taxonomic bacterial diversity in black shale and the first insight into the diversity of archaea and fungi inhabiting black shale.

### General characteristics of metagenome and metaproteome

To identify the metabolic processes of LMC as well as indicate the microorganisms crucial for the oxidation of fossil hydrocarbons and sulfide minerals, an analysis of its metagenome and metaproteome was conducted.

In the metagenome of LCM, 446,915 bacterial, 3,529 archaeal and 1,415 fungal PEGs were identified. Interestingly, among the identified PEGs, 112412 (25%), 1245 (35%), and 866 (61%) bacterial, archeal and fungal proteins, respectively, had no meaningful annotation (described as either hypothetical or unknown protein, or protein of unknown function).

The metaproteome of LMC comprised 788 bacterial, 694 archaeal, and 655 fungal proteins. Among these, 376 bacterial, 503 archaeal, and 455 fungal proteins were identified as high-confidence proteins matching two or more peptides or possessing one characteristic peptide (occurring only in this specific protein). About 60% of bacterial and archaeal high-confidence proteins, as well as 28% of fungal high-confidence proteins, constituted housekeeping proteins. The examined LMC was also enriched in novel proteins of unknown function (hypothetical proteins) representing nearly 70% of fungal and 30–25% of bacterial and archaeal high-confidence proteins.

### Oxidative metabolism of fossil hydrocarbons

**Figure 3:**
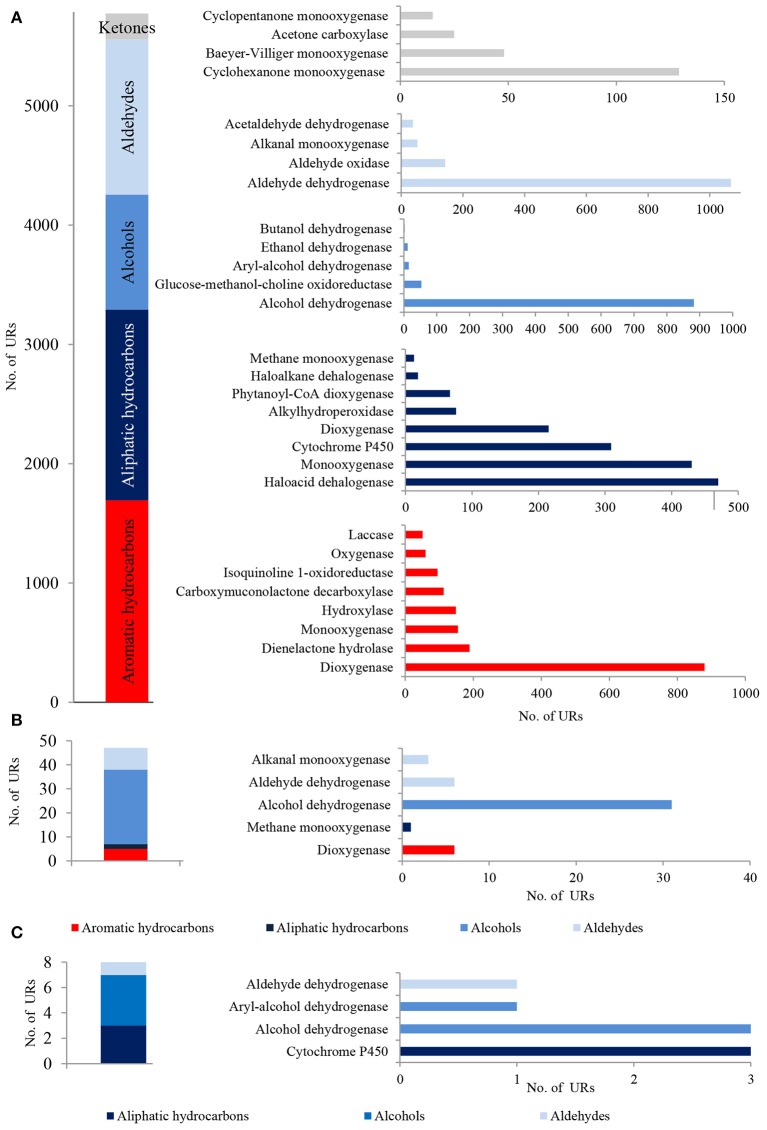
The unique bacterial **(A)**, archaeal **(B)** and fungal **(C)** PEGs detected in LMC involved in the oxidative metabolism of hydrocarbons, alcohols, aldehydes and ketones. The detailed list of the PEGs is presented in Tables S1–S3 and in Supplementary Data Sheet 1.

In total, 5,748 bacterial, 47 archaeal and 8 fungal PEGs identified in the metagenome of LMC were potentially involved in the oxidative metabolism of aromatic and aliphatic hydrocarbons, as well as the metabolism of alcohols, aldehydes and ketones (Figure 3). A detailed summary of hydrocarbon metabolism-related PEGs is presented in Supplementary Data Sheet 1 and Tables S1–S3. Among these, bacterial mono- and dioxygenases (2373 URs), aldehyde dehydrogenases (1106 URs), and alcohol dehydrogenases (912 URs) were dominant (Figure 3A). Bacterial laccase encoding genes (51 URs) were also identified. In the archaeal PEGs the genes encoding alcohol dehydrogenases prevailed (31 URs) (Figure 3B), and in the fungal PEGs the genes encoding alcohol dehydrogenases (3 URs) and cytochrome P450 (3 URs) were predominant (Figure 3C).

**Figure 4:**
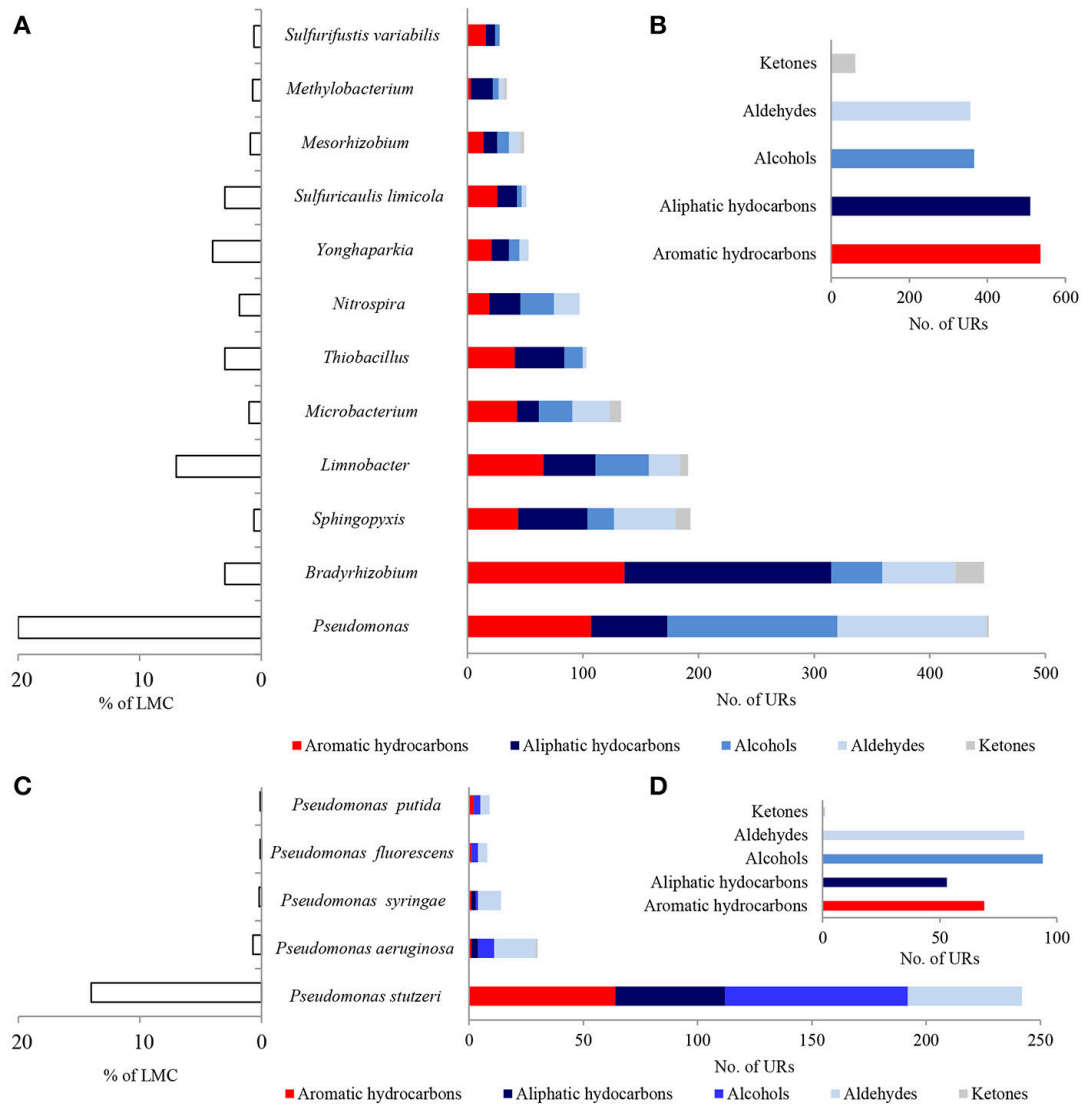
Taxonomic origin **(A,C)** and number **(B,D)** of bacterial PEGs detected in the dominating genera and species of bacteria of LMC and in bacteria belonging to genus *Pseudomonas* related to the oxidative metabolism of hydrocarbons, alcohols, aldehydes and ketones. The detailed list of PEGs is presented in Tables S1–S3 and Supplementary Data Sheet 1; the detailed taxonomic diversity of bacteria is presented in Supplementary Presentation 1.

A considerable number of identified PEGs potentially involved in the metabolism of hydrocarbons were derived from *Pseudomonas* spp. (451 URs) and *Bradyrhizobium* spp. (447 URs), as well as *Sphingopyxis* spp. (193 URs), *Limnobacter* spp. (191 URs), and *Microbacterium* spp. (133 URs) (Figure 4A; Tables S1–S3). *Bradyrhizobium* PEGs could play a crucial role in the oxidation of aliphatic (179 URs) and aromatic hydrocarbons (136 URs) present in black shales. Among the *Pseudomonas*, PEGs were also involved in the oxidative metabolism of aromatic (107 URs) and aliphatic (66 URs) hydrocarbons, as well as alcohols (147 URs) and aldehydes (129 URs) (Figures 4A,B). PEGs of *Bradyrhizobium* were derived from various strains, whereas most *Pseudomonas* PEGs originated from bacteria belonging to the *P. stutzeri* group (Figures 4C,D). Archaeal and fungal PEGs potentially engaged in hydrocarbon metabolism were mainly derived from *Nitrososphaera* spp. (38 URs) and *Aspergillus* sp. (4 URs), respectively (Tables S2 and S3). The examined LMC is the first described subsurface community comprised of *Limnobacter* spp. potentially involved in hydrocarbon metabolism (Figure 4A). The identified *Limnobacter* genes encoded cytochrome P450, phenol 2-monooxygenases, monooxygenase flavin-binding family proteins, 4-hydroxyacetophenone monooxygenases, quercetin 2,3-dioxygenases, catechol 2,3-dioxygenases, 4-carboxymuconolactone decarboxylases and haloacid dehalogenases (Supplementary Data Sheet 1). Phenol-degrading *Limnobacter* strains possessing catechol 2,3-dioxygenases had previously been isolated from Baltic Sea surface water (Vedler et al., 2013). Another bacteria potentially participating in hydrocarbon metabolism was *Yonghaparkia* spp. (Figure 4A). Bacteria belonging to this genus had previously been isolated from microbial communities detected in a gold mine (Linglong, China) (Li et al., 2016) and a uranium mine (Athabasca Basin, Canada) (Bondici et al., 2013), as well as from the black shale mentioned earlier (Chengkou County, China) (Li et al., 2014). Moreover, surprisingly numerous hydrocarbon metabolism-related PEGs, including dioxygenases, laccases, dienelactone hydrolases and haloacid dehalogenases, originated from two chemolithotrophic bacteria, *S. limicola* and *Sulfurifustis variabilis* (Figure 4A; Supplementary Data Sheet 1), isolated for the first time by Kojima et al. (2015, 2016).

**Figure 5:**
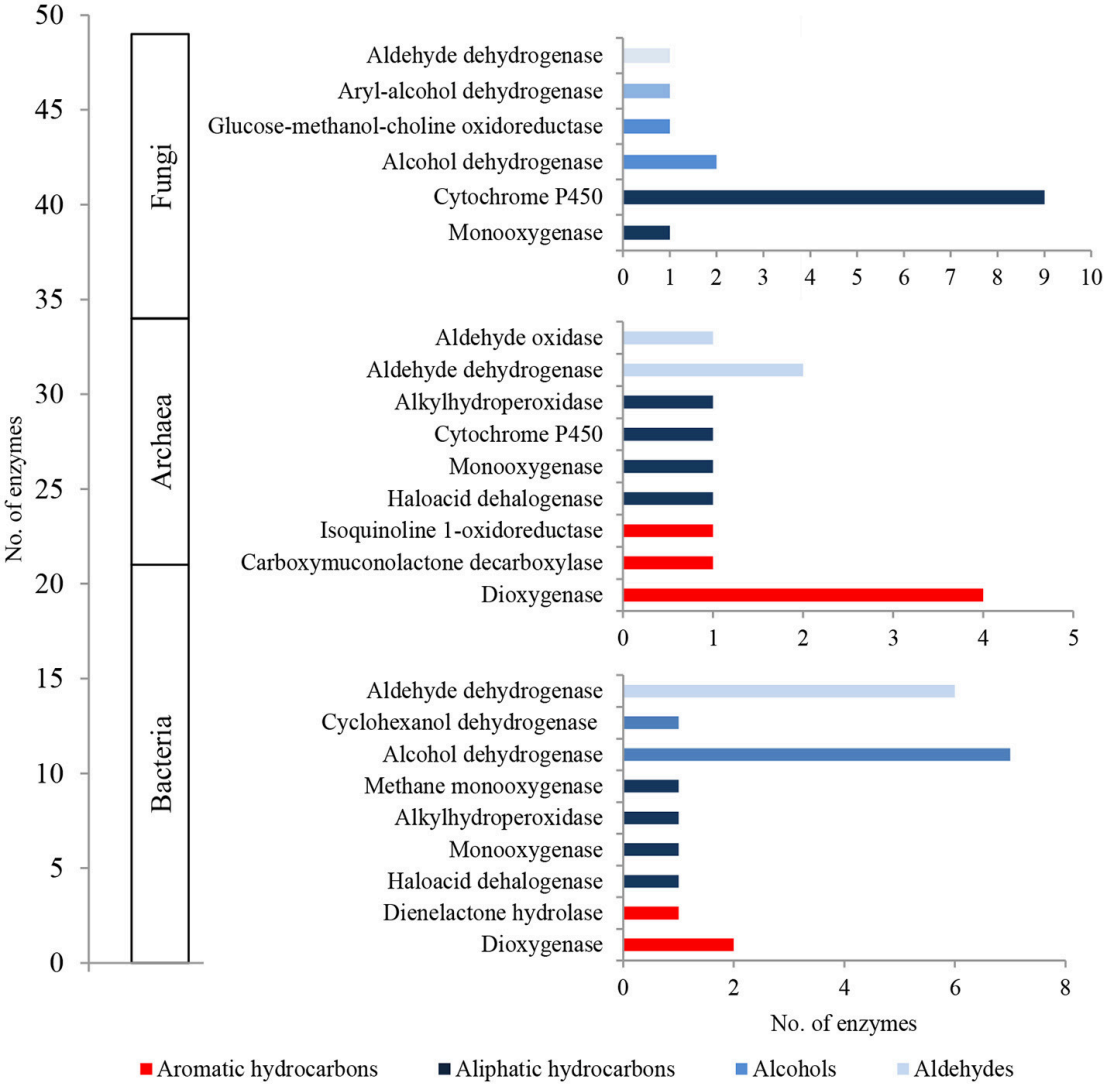
The unique bacterial, archaeal and fungal enzymes detected in the metaproteome of LMC involved in the oxidative metabolism of hydrocarbons, alcohols and aldehydes. The detailed list of the detected enzymes is presented in Tables S4–S6.

The metaproteome of LMC comprised 21 bacterial, 13 archaeal and 15 fungal enzymes related to hydrocarbon metabolism (Figure 5; Tables S4–S6). They were dominated by bacterial enzymes participating in the aerobic metabolism of alcohols and aldehydes, including seven alcohol dehydrogenases and six aldehyde dehydrogenases in particular. Four detected dioxygenases were only archaeal enzymes, whereas nine cytochromes P450 were identified primarily among fungal enzymes.

Furthermore, we also detected a number of bacterial, archaeal and fungal PEGs, as well as enzymes involved in the utilization of carboxylic acids (e.g., enzymes of the β-oxidation process, such as acyl-CoA dehydrogenase, 3-hydroxyacyl-CoA dehydrogenase and long-chain fatty acid - CoA ligase) and esters (e.g., sulfatase, carboxyesterase, and lipase) (unpresented results).

The presence of the above PEGs, as well as enzymes, demonstrates the potential ability of LMC toward the oxidative metabolism of aromatic and aliphatic hydrocarbons. Most of the identified PEGs and enzymes belong to aliphatic hydrocarbon metabolism pathways which consist of terminal or subterminal oxidation of aliphatic hydrocarbons to carboxylic acids (fatty acids) utilized in the β-oxidation process (Ji et al., 2013).

Investigations into fossil organic matter transformation occurring in black shales are limited to the studies by Petsch et al. (2001, 2005), Seifert et al. (2011), and Berlendis et al. (2014). The first of these proved that microorganisms inhabiting terrestrial Late Devonian New Albany Shale (USA) assimilate ancient organic carbon derived from organic matter in the shale. Berlendis et al. (2014) detected nag-like genes of *R. picketii* inhabiting terrestrial Lower Autunian Autun black shale (France) which could encode for dioxygenase genes. A research study conducted by Seifert et al. (2011) unequivocally confirmed the ability of indigenous bacteria to mobilize carbon from a Lower Silurian Variscan graptolite shale formation (Germany) and incorporate it into fatty acid metabolism. However, the molecular factors responsible for these processes were not indicated, nor was the influence of these processes on properties of kerogen.

### Oxidative metabolism of inorganic sulfur compounds

**Figure 6:**
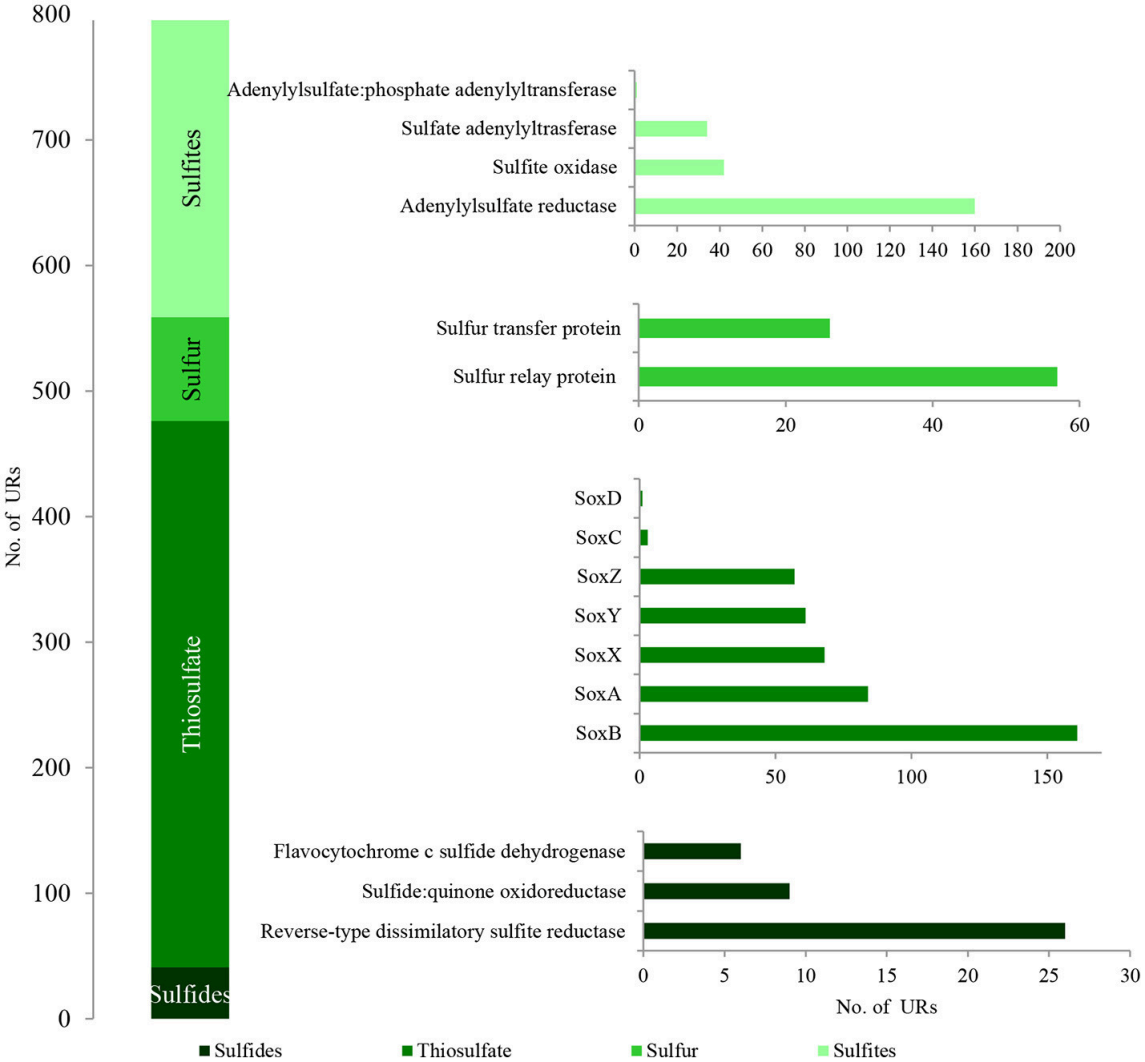
The unique bacterial PEGs detected in LMC involved in the oxidative metabolism of sulfide minerals. The detailed list of PEGs is presented in Table S1 and Supplementary Data Sheet 1.

796 bacterial PEGs related to the oxidative metabolism of reduced sulfur compounds were detected in the metagenome of the studied LMC (Figure 6). Archaeal and fungal PEGs related to this process were not detected. A detailed summary of PEGs involved in this process is presented in Supplementary Data Sheet 1 and Table S1.

**Figure 7:**
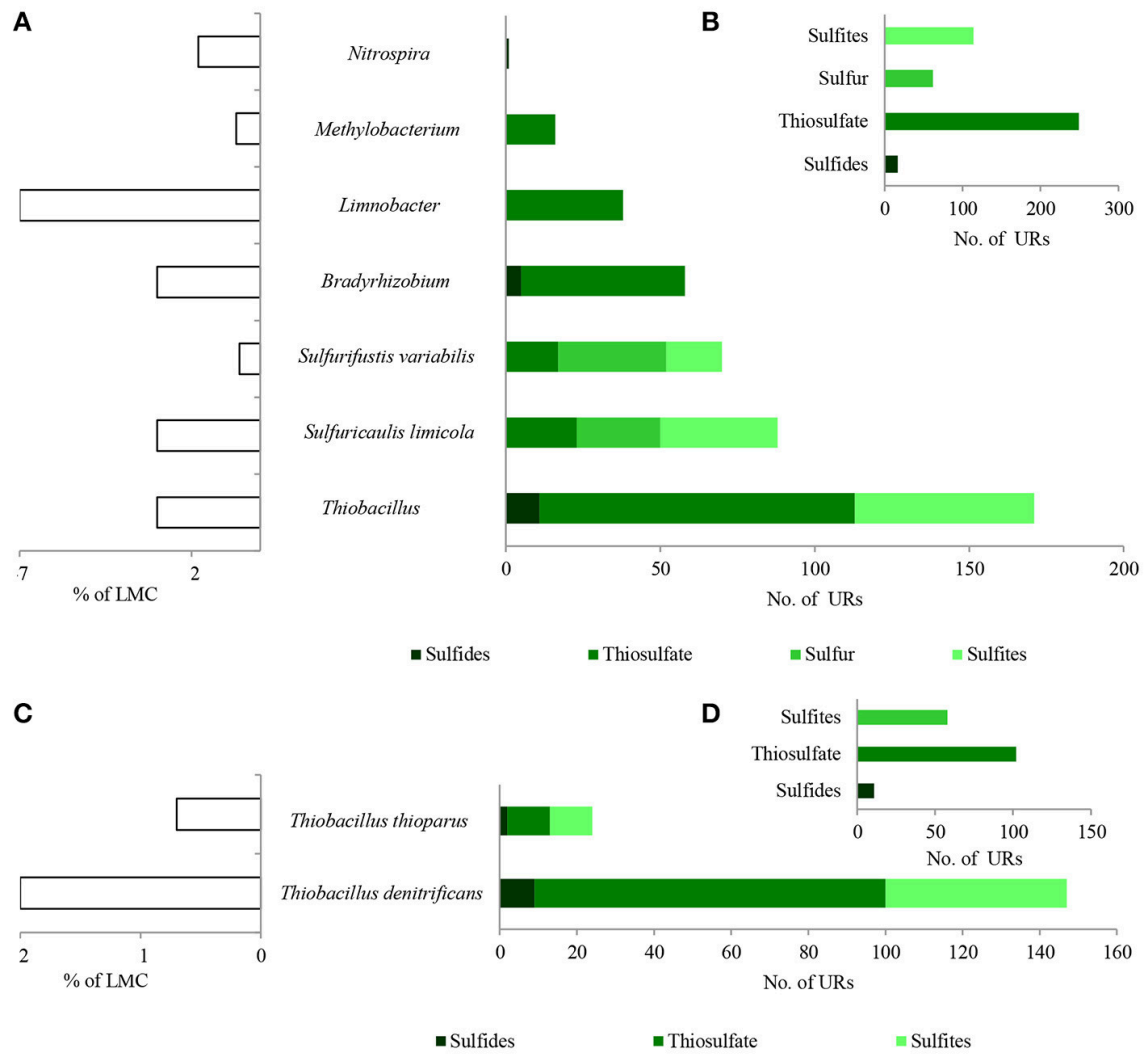
Taxonomic origin **(A,C)** and number **(B,D)** of bacterial PEGs detected in the dominating genera and species of bacteria of LMC and two species of *Thiobacillu*s related to the oxidative metabolism of sulfides, thiosulfate, sulfur and sulfites. The detailed list of PEGs is presented in Table S1 and Supplementary Data Sheet 1; the detailed taxonomic diversity of bacteria is presented Supplementary Presentation 1. URs, unique reads.

Among the identified PEGs, the most important were those potentially involved in the dissimilative oxidation of sulfides (Ghosh and Dam, 2009). Sulfide:quinone oxidoreductase (9 URs) involved in sulfide oxidation to sulfur was detected in *T. denitrificans, Bradyrhizobium* spp., and *Nitrospira* spp. (Figures 7A,B; Table S1). In addition, flavocytochrome c sulfide dehydrogenase (6 URs) involved in sulfide oxidation was also identified in *T. denitrificans* (Figures 7C,D). This enzyme catalyzes the conversion of sulfides to elemental sulfur or polysulfide and it is assumed to be the main sulfide oxidizing system in *Thiobacillus* genus. Furthermore, reverse-type dissimilatory siroheme sulfite reductase (RDsr) (26 URs) participating in the reverse system of dissimilatory sulfite reduction was identified in *T. thioparus* (Figures 7C,D).

Direct oxidation of thiosulfate to sulfate catalyzed by the thiosulfate-oxidizing multi-enzyme system, also known as the Sox enzyme system (Friedrich et al., 2001), was also detected in LMC (435 URs) (Figure 6). PEGs for all components of the Sox system (*soxX, soxA, soxB, soxC, soxD, soxY*, and *soxZ*) were present in the metagenome of LMC. These were detected mainly in bacteria belonging to genus *Thiobacillus* (102 URs), especially *T. denitrificans* (91 URs) as well as *Bradyrhizobium* (53 URs), *Limnobacter* (38 URs), *S. limicola* (23 URs), *S. variabilis* (17 URs), and *Methylobacterium* (16 URs) (Figure 7). To conclude, in the studied LMC, thiosulfate oxidation in both known pathways (to sulfates or to sulfates and sulfur) is potentially possible (Wodara et al., 1997). Beside thiosulfate, this molecular mechanism has been proved to oxidize other reduced sulfur species such as sulfide, elemental sulfur, sulfite and tetrathionate (Wodara et al., 1997).

The sulfite oxidation pathway mediated by adenylylsulfate reductase (160 URs), sulfate adenylyltransferase (34 URs) and adenylylsulfate:phosphate adenylyltransferase (1 UR) were detected in *S. limicola, T. denitrificans, S. variabilis*, and *T. thioparus* (Kappler and Dahl, 2001) (Figure 7A). Furthermore, a universal system of direct sulfite oxidation by sulfite oxidase (Kappler and Dahl, 2001) (42 URs) was identified in *S. limicola, S. variabilis* and *Rhodoplanes* sp. (Table S1).

Finally, PEGs potentially participating in the oxidation of sulfur - sulfur relay proteins (57 URs) and sulfur transfer proteins (26 URs) were detected in *S. limicola* and *S. variabilis* (Figure 7A).

In summary, a considerable number of genes potentially involved in inorganic sulfur metabolism originated from *Thiobacillus* chemolithotrophic neutrophilic bacteria (171 URs), mostly *T. denitrificans* (147 URs) (Figures 7C,D) and were potentially involved in sulfide, thiosulfate and sulfite oxidation. PEGs related to the oxidation of these three reduced sulfur compounds also originated from two chemolithotrophic bacteria: *S. limicola* (88 URs), and *S. variabilis* (70 URs) (Figure 7A). Sulfur compound oxidation PEGs were also identified in *Bradyrhizobium* spp. (58 URs), *Limnobacter* spp. (38 URs) and *Methylobacterium* spp. (16 URs) (Figure 7A), although they were mostly involved in thiosulfate oxidation. Some of these bacteria are known to be capable of chemolithoheterotrophic growth, oxidizing sulfur compounds in the presence of an organic carbon source (Spring et al., 2001; Lu et al., 2011).

In the metaproteome of the tested LMC, only two bacterial enzymes (cytochrome SoxA and DsrH protein) related to the oxidative metabolism of inorganic sulfur compounds were identified (Table S4). Archaeal and fungal enzymes related to this process were not detected.

Taken together, the presence of the described PEGs shows the potential role of LMC in the dissimilative oxidation of reduced sulfur compounds which are used as energy sources and electron donors. Several reduced sulfur compound-oxidizing pathways were detected (Figure 6). The first potential pathway is the most widespread system based on the oxidation of sulfides, sulfur and thiosulfate via sulfites to sulfates, while the second one is direct oxidation of thiosulfate to sulfates (Ghosh and Dam, 2009). Two potential pathways of sulfite oxidation were also detected—direct oxidation to sulfates and indirect oxidation with adenosine phosphosulfate as an intermediate (Kappler and Dahl, 2001). The very small number of enzymes associated with sulfur oxidation is surprising; however, this can be explained firstly by the fact that the content of key sulfur-oxidizing bacteria (*Thiobacillus* spp., *S. limicola, S. variabilis*) in LMC is small (about 7%) in comparison to main heterotrophic bacteria participating in hydrocarbon metabolism (*Pseudomonas* spp., *Bradyrhizobium* spp., *Limnobacter* spp., *Microbacterium* spp., *Sphingopyxis* spp.) (about 30%). Also the number of detected PEGs potentially involved in sulfur-compound oxidation is about seven times lower (796) than those involved in hydrocarbon metabolism (5,828). Secondly, it is supposed that the lack of expression of these enzymes may be related to the lack of reduced sulfur compounds in the studied weathered black shale, which is described in the next subsection of results.

### Geochemistry of the studied black shale

In order to confirm the potential metabolic activity of LMC, we examined the geochemical properties of the 12-year-old weathered black shale and we compared these to the properties of the unweathered shale studied earlier (Stasiuk et al., 2017). Our research included the comparison of: (i) the properties of kerogen and organic carbon content, (ii) the qualitative and semi-quantitative comparative composition of extractable organic matter, and (iii) the sulfur content and its speciation. The potential metabolic activity of LMC described above seems to correlate with geochemical properties of the studied black shale.

**Figure 8:**
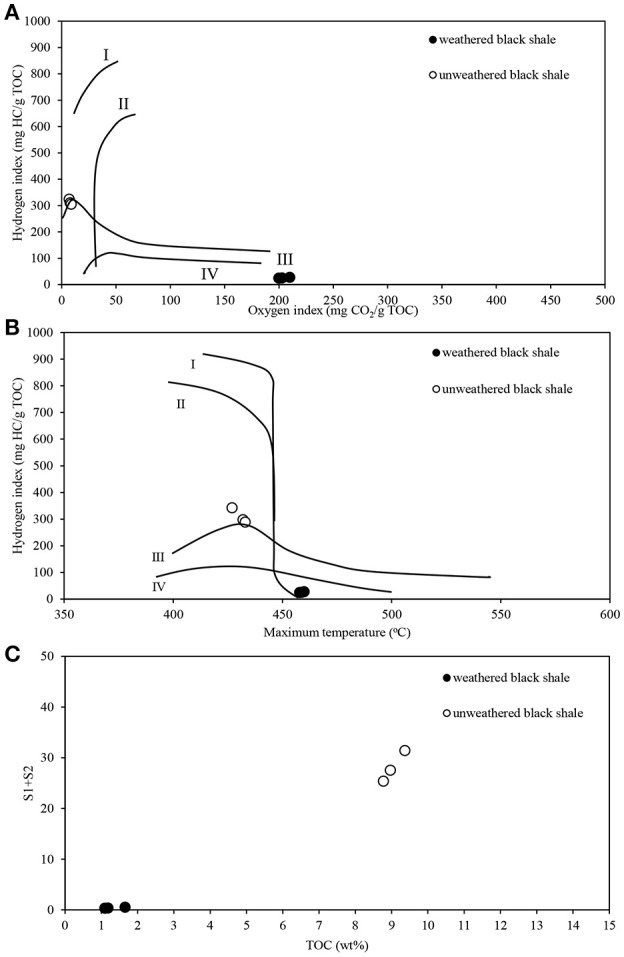
Pyrolytic characteristics of the weathered and unweathered black shale kerogen, obtained during Rock-Eval analysis. **(A)** Modified Van Krevelen diagram with the correlation of hydrogen (HI) and oxygen (OI) indexes; **(B)** HI and Tmax crossed-diagram; **(C)** Correlation of TOC and hydrocarbon potential (S1+S2). The detailed data of Rock-Eval analysis are presented in Table S7. The unweathered black shale is described in detail in Stasiuk et al. (2017).

**Figure 9:**
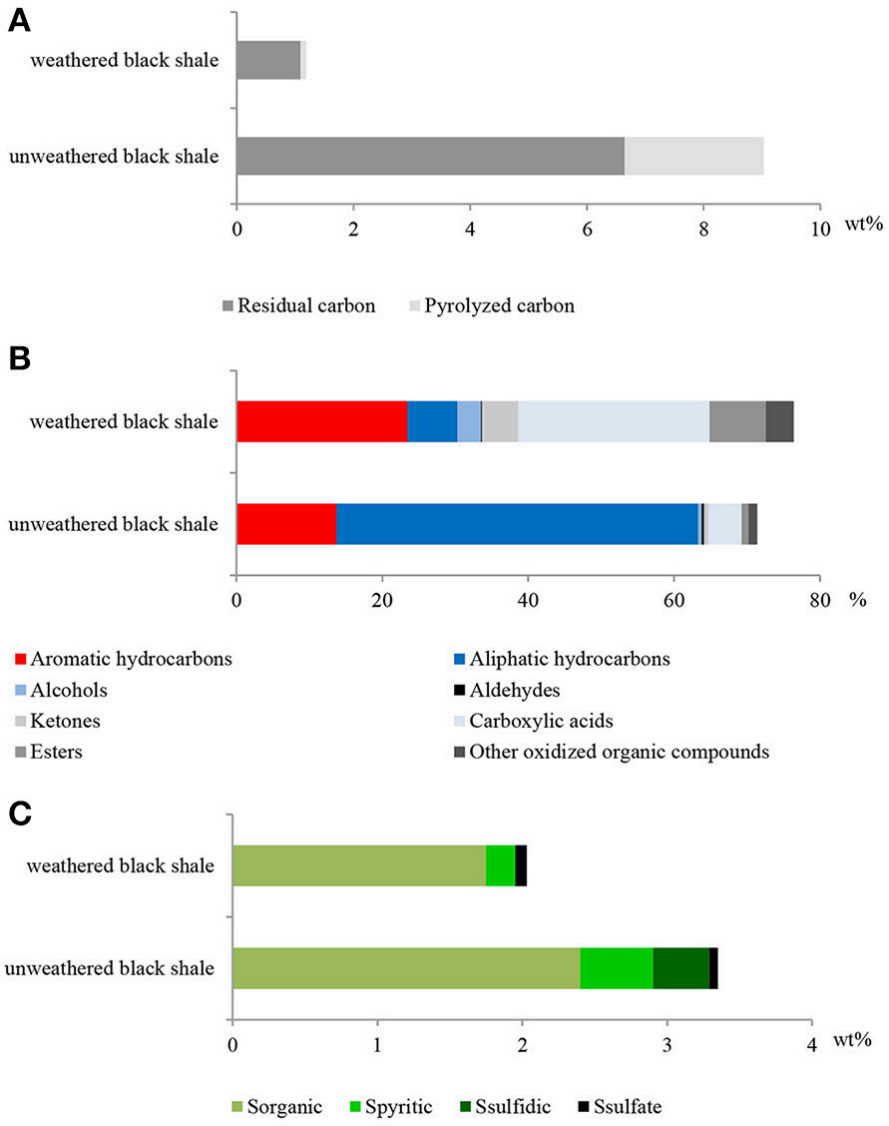
The content of organic carbon represented by pyrolized and residual carbon **(A)**; selected classes of extractable organic compounds **(B)**; content of sulfur and its speciation **(C)** in the weathered and unweathered black shales. The unweathered black shale is described in detail in Stasiuk et al. (2017).

The pyrolytic analysis of black shale by the Rock-Eval method showed an unusually high ratio of oxygen to hydrogen in this rock compared with that of the unweathered black shale (Figure 8; Table S7) (Stasiuk et al., 2017), which may confirm the strong oxidation of kerogen. At the same time, we observed a considerably reduced (about 86%) content of C_organic_ dominated by residual carbon (91%) (Figure 9A). Similarly, a reduced content of free hydrocarbons (about 96%) and hydrocarbon potential (about 91%) confirmed the dehydrogenation of kerogen. All these parameters suggest the change of its type from oil-prone type II to a non-productive, residual and hydrogen-free kerogen IV, which is usually regarded as a product of strong chemical and/or biological degradation of kerogen type III or II.

Also, the composition of extractable organic matter in the studied black shale was unusual and the amount of this mobile matter was also higher compared with that of the unweathered black shale (Figure 9B; Table S7). The content of aromatic hydrocarbons and derivatives increased and at the same moment the content of aliphatic hydrocarbons decreased. These changes could be related to kerogen weathering and partial biodegradation of mainly aliphatic hydrocarbons. About 45% of all extractable organic compounds identified in this rock were oxygenated alcohols, aldehydes, ketones, esters, and above all carboxylic acids (Figure 9, Figure S2, Tables S7, S8). In the unweathered black shale, the content of oxidized organic compounds was estimated at 8–10% (Stasiuk et al., 2017). Numerous oxygenated organic compounds could have been formed with the participation of LMC. The identified carboxylic acids have been created as a result of the oxidative metabolism of fossil hydrocarbons present in black shales. This process can be proved by the detected intermediate products of hydrocarbon degradation, such as alcohols, ketones, aldehydes and esters (Tables S7, S8) (Leahy and Colwell, 1990; Rojo, 2009) as well as by the described PEGs and enzymes (Figures 3, 5). This result is consistent with the results of our previous long-term (365 days) laboratory studies conducted with a consortium of cultivable heterotrophic bacteria isolated from the Kupferschiefer black shale composed of *Pseudomonas* spp., *Microbacterium* spp., *Bacillus* sp., and *Acinetobacter* sp. In that studies the direct role of bacteria in the oxidation of fossil organic matter and changes of the kerogen type and hydrocarbon potential as well changes in the content and composition of extractable organic matter was showed (Stasiuk et al., 2017). These changes were not observed in the sterile control, where the black shale was exposed to the oxygen and water.

The potential oxidative metabolism of sulfidic minerals by LMC suggested by metagenomic studies (Figures 6, 7) is correlated with the lack of sulfidic sulfur and also loss of pyritic sulfur (0.2 wt%) in the studied black shales, which are normally abundant in the Kupferschiefer (0.39 and 0.50 wt%, respectively) (Figure 9C; Table S7) (Matlakowska et al., 2012). In the tested weathered black shale, no common ore sulfide minerals were detected either using X-ray diffraction or reflected-light microscopy (unpresented results). However, the content of sulfates in the weathered black shale was also very low. This could be related to: (i) the mobilization of sulfates by mine waters, (ii) the dissimilative reduction of sulfates to H_2_S by sulfate reducing bacteria present in LMC (Supplementary Presentation 1), or (iii) the assimilative reduction of sulfates by microorganisms. The first two processes are the most probable, because in the studied black shale we observed a significant decrease (about 39%) in the total sulfur content and simultaneous loss of organic sulfur (about 27%) compared to the unweathered black shale (Figure 9C).

## Conclusions

Two catabolic microbial processes performed by two trophic groups of microorganisms were described in the deep subterrestrial black shale. One is the enzymatic dissimilative oxidation of fossil hydrocarbons mediated mostly by chemoorganotrophs. The other is the dissimilative enzymatic oxidation of primary reduced sulfur compounds conducted by chemolithotrophs. Twelve genera of bacteria dominate in LMC and play a crucial role in these processes (Figures 4, 7). The described microbial processes potentially play a role in the weathering of kerogen and sulfide minerals and cause organic carbon and inorganic sulfur mobilization from black shales.

## Data deposition

The results of metagenomic DNA sequencing are deposited at: http://www.ebi.ac.uk/ena/data/view/PRJEB21522.

## Author contributions

RM: concept of research and funding; GB, AW, and RM: Sampling; AW: DNA isolation protein isolation, and organic compound extraction; MK and AF: DNA sequencing; ML: Bioinformatics analyses; RM and AW: Manuscript preparation.

### Conflict of interest statement

The authors declare that the research was conducted in the absence of any commercial or financial relationships that could be construed as a potential conflict of interest.
