# Supplementary material for: The Oxidative Metabolism of Fossil Hydrocarbons and Sulfide Minerals by the Lithobiontic Microbial Community Inhabiting Deep Subterrestrial Kupferschiefer Black Shale
Source: Front Microbiol. 2018 May 15;9:972. doi: 10.3389/fmicb.2018.00972 (PMC5962744; doi:10.3389/fmicb.2018.00972)
Supplement: Supplementary file 1 [file Image_1.PDF]

A

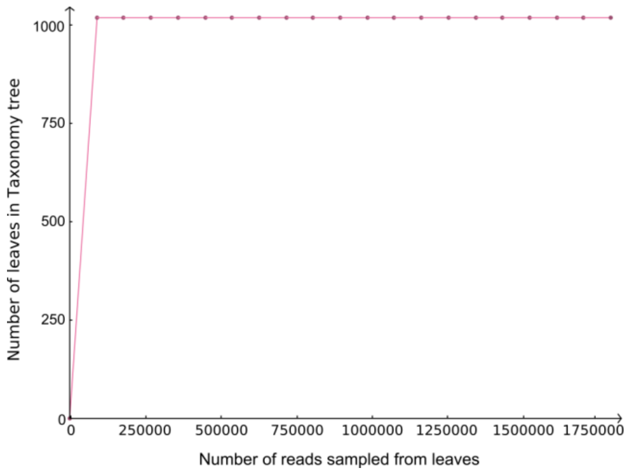

B

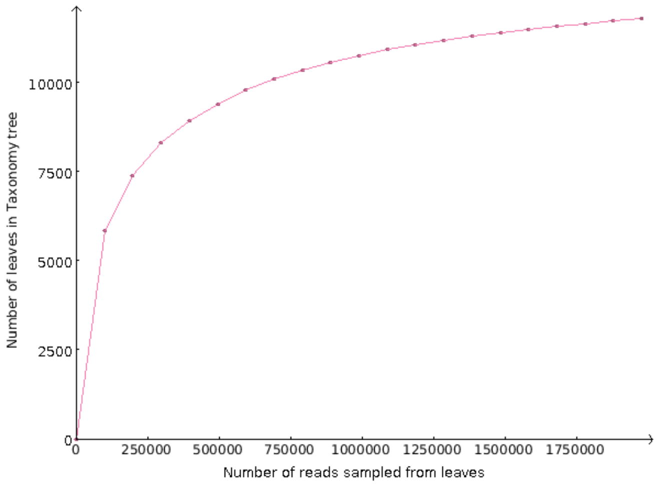

Fig. S1. Rarefaction curves for the metagenomic analysis of LMC inhabiting black shale. Curve A was plotted with coverage cut-off used in the taxonomic diversity analysis (207 reads per reported taxon); curve B depicts rarefaction on sample without coverage cut-off. The plots prove that only highly reliable calls were reported and that sample was sequenced with sufficient depth (the slight linear slope of the curve B observable for read samples bigger than ~1100000 reads most likely results from sequencing errors or wrong DIAMOND assignments of some reads. Curve A becomes quickly saturated and reaches considerably lower values for leaves in Taxonomy tree, showing that only highly reliable fraction of taxons were reported.
