# Supplementary material for: The Oxidative Metabolism of Fossil Hydrocarbons and Sulfide Minerals by the Lithobiontic Microbial Community Inhabiting Deep Subterrestrial Kupferschiefer Black Shale
Source: Front Microbiol. 2018 May 15;9:972. doi: 10.3389/fmicb.2018.00972 (PMC5962744; doi:10.3389/fmicb.2018.00972)
Supplement: Supplementary file 2 [file Image_2.PDF]

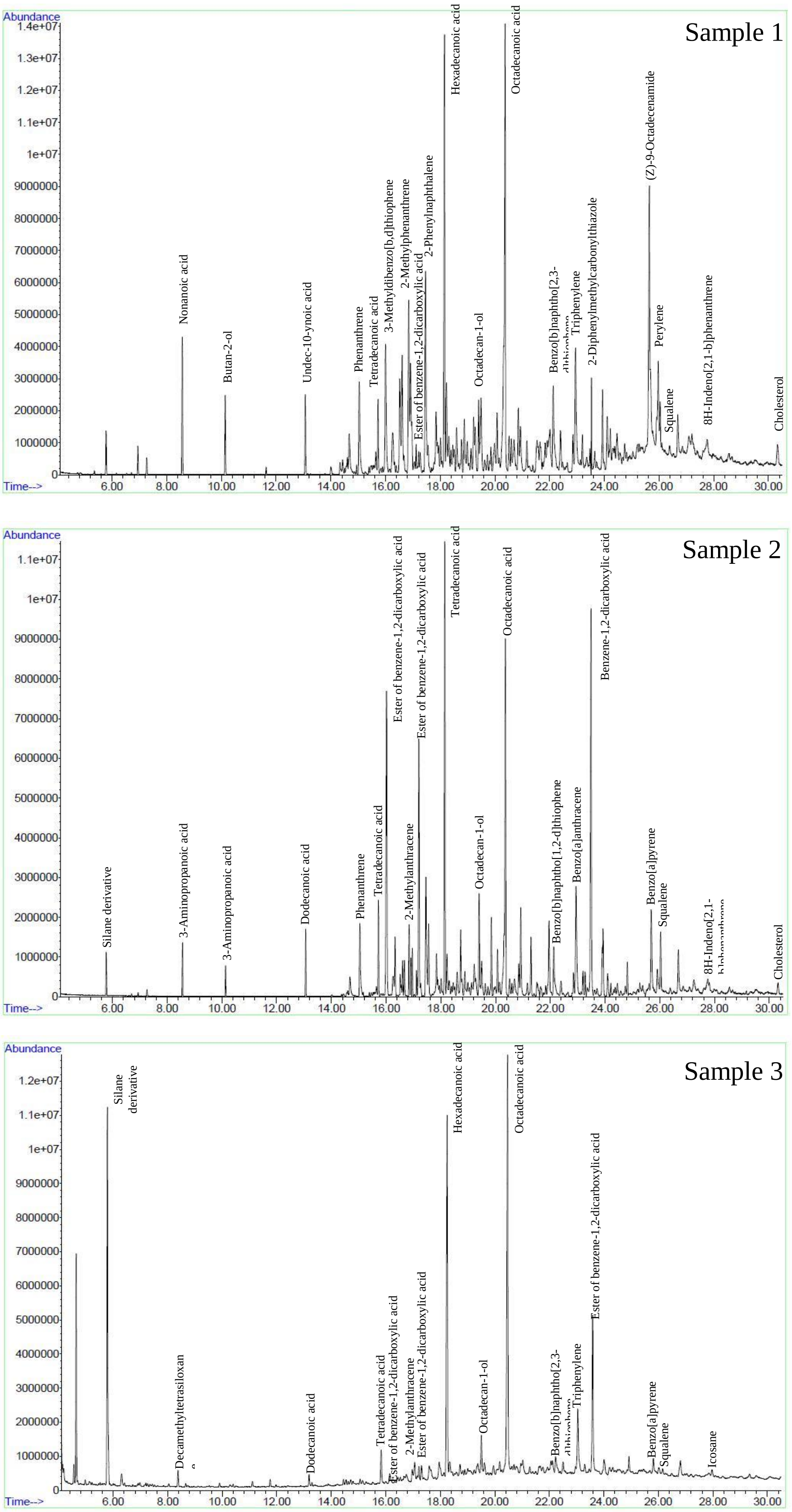

Fig. S2. Total ion current chromatograms of triplicate dichloromethane/methanol extracts of organic compounds from the studied black shale. Selected organic compounds are listed in Table S9 and S10.
