# Supplementary material for: The Oxidative Metabolism of Fossil Hydrocarbons and Sulfide Minerals by the Lithobiontic Microbial Community Inhabiting Deep Subterrestrial Kupferschiefer Black Shale
Source: Front Microbiol. 2018 May 15;9:972. doi: 10.3389/fmicb.2018.00972 (PMC5962744; doi:10.3389/fmicb.2018.00972)
Supplement: Supplementary file 4 [file Table_1.DOCX]

Table S1. Bacterial PEGs related to oxidative metabolism of hydrocarbons and sulfide minerals identified in the metagenome of LMC and the dominant species and genera from which they originated.

| **Protein name** | | **Unique reads** | | **All reads** | **Protein specific name** | **Species: unique reads/all reads** | **Genera: unique reads/all reads** |
| --- | --- | --- | --- | --- | --- | --- | --- |
| **OXIDATIVE METABOLISM OF HYDROCARBONS** | | | | | | | |
| Acetaldehyde dehydrogenase | | 38 | | 70 | Acetaldehyde dehydrogenase;  Acetaldehyde dehydrogenase (acetylating);  Acetaldehyde dehydrogenase (acylating);  Acetaldehyde dehydrogenase (acylating) (plasmid);  Acetaldehyde dehydrogenase 2;  Bifunctional acetaldehyde-CoA/alcohol dehydrogenase;  MULTISPECIES: acetaldehyde dehydrogenase. | *Novosphingobium* sp. PP1Y: 3/3  *Limnobacter* sp. CACIAM 66H1: 2/10  *Pseudomonas* sp. TTU2014-080ASC: 2/3  *Novosphingobium* sp. Fuku2-ISO-50: 2/2  *Sphingopyxis macrogoltabida*: 1/1 | *Novosphingobium*: 5/6  *Sphingopyxis*: 3/5  *Limnobacter*: 2/10  *Pseudomonas*: 2/4  *Sphingobium*: 2/3 |
| Acetone carboxylase | | 25 | | 26 | Acetone carboxylase beta subunit;  Acetone carboxylase subunit alpha;  N-methylhydantoinase A/acetone carboxylase subunit beta;  N-methylhydantoinase A/acetone carboxylase, beta subunit;  N-methylhydantoinase A/oxoprolinase/acetone carboxylase, beta subunit;  N-methylhydantoinase B/acetone carboxylase subunit alpha;  N-methylhydantoinase B/acetone carboxylase, alpha subunit. | *Rhodoplanes* sp. Z2-YC6860: 11/11  *Nevskia soli*: 3/3  *Solimonas variicoloris*: 2/2  *Rubellimicrobium thermophilum*: 2/2  *Methylobacterium aquaticum*: 1/1 | *Rhodoplanes*: 11/11  *Nevskia*: 3/3  *Solimonas*: 2/2  *Rubellimicrobium*: 2/2  *Methylobacterium*: 1/1 |
| Alcohol dehydrogenase | | 882 | | 2970 | ABC transporter, substrate binding protein, PQQ-dependent alcohol dehydrogenase system;  AF176640_2 alcohol dehydrogenase;  Alcohol dehydrogenase;  Alcohol dehydrogenase (acceptor);  Alcohol dehydrogenase (azurin);  Alcohol dehydrogenase (cytochrome c);  Alcohol dehydrogenase (plasmid);  Alcohol dehydrogenase 1 (alcohol dehydrogenase I) (ADH I);  Alcohol dehydrogenase 2 (plasmid);  Alcohol dehydrogenase AdhP;  Alcohol dehydrogenase class III;  Alcohol dehydrogenase class III, S-(hydroxymethyl)glutathione dehydrogenase / alcohol dehydrogenase;  Alcohol dehydrogenase class-3;  Alcohol dehydrogenase GroES domain protein;  Alcohol dehydrogenase GroES domain protein, L-iditol 2-dehydrogenase;  Alcohol dehydrogenase GroES domain-containing protein;  Alcohol dehydrogenase GroES-like;  Alcohol dehydrogenase GroES-like domain protein, partial;  Alcohol dehydrogenase superfamily, zinc-containing:alcohol dehydrogenase, zinc-containing;  Alcohol dehydrogenase zinc-binding domain protein;  Alcohol dehydrogenase zinc-binding domain-containing protein;  Alcohol dehydrogenase zinc-binding domain-containing protein, NADPH2:quinone reductase;  Alcohol dehydrogenase, alcohol dehydrogenase;  Alcohol dehydrogenase, partial;  Alcohol dehydrogenase, zinc-binding domain protein;  Alcohol dehydrogenase, zinc-binding protein;  Alcohol dehydrogenase, zinc-containing;  Alcohol dehydrogenase, zinc-containing, partial;  Aldehyde-alcohol dehydrogenase;  Aldehyde-alcohol dehydrogenase family protein;  Bifunctional protein zinccontaining alcohol dehydrogenase quinone oxidoreductase NADPHquinone reductase EC 111 Similar to arginate lyase CDS;  Bifunctional protein: zinc-containing alcohol dehydrogenase; quinone oxidoreductase (NADPH:quinone reductase) ; Similar to arginate lyase;  Class 3 alcohol dehydrogenase;  Iron-containing alcohol dehydrogenase;  Michael hydratase/alcohol dehydrogenase large subunit;  MULTISPECIES: alcohol dehydrogenase;  MULTISPECIES: quinonprotein alcohol dehydrogenase;  MULTISPECIES: S-(hydroxymethyl)glutathione dehydrogenase/class III alcohol dehydrogenase;  MULTISPECIES: zinc-binding alcohol dehydrogenase;  MULTISPECIES: zinc-containing alcohol dehydrogenase;  MULTISPECIES: zinc-dependent alcohol dehydrogenase;  NAD(P)-dependent alcohol dehydrogenase;  NAD(P)-dependent dehydrogenase, short-chain alcohol dehydrogenase family;  NAD-dependent alcohol dehydrogenase;  NADH-dependent alcohol dehydrogenase;  NADP-dependent alcohol dehydrogenase;  NADP-dependent alcohol dehydrogenase c;  NDMA-dependent alcohol dehydrogenase, Rxyl_3153 family protein;  Phosphonate catabolism associated alcohol dehydrogenase;  PQQ-dependent alcohol dehydrogenase;  PQQ-dependent alcohol dehydrogenase PedH;  Probable alcohol dehydrogenase (acceptor);  Probable alcohol dehydrogenase class III;  Probable iron-containing alcohol dehydrogenase;  Probable zinc-type alcohol dehydrogenase AdhD;  Putative alcohol dehydrogenase;  Putative alcohol dehydrogenase AdhA;  Putative alcohol dehydrogenase, zinc-containing (plasmid);  Putative alcohol dehydrogenase, Zn-dependent and NAD(P)-binding;  Putative NAD-dependent alcohol dehydrogenase;  Putative zinc-type alcohol dehydrogenase-like protein YjmD;  Putative zink-containing alcohol dehydrogenase;  Quino(hemo)protein alcohol dehydrogenase;  Quino(hemo)protein alcohol dehydrogenase, PQQ-dependent;  Quinohemoprotein alcohol dehydrogenase ADH IIB precursor;  Quinonprotein alcohol dehydrogenase;  Quinonprotein alcohol dehydrogenase, partial;  Quinonprotein alcohol dehydrogenase-like;  Quinoprotein alcohol dehydrogenase;  Rhamnulose-1-phosphate aldolase/alcohol dehydrogenase;  S-(hydroxymethyl)glutathione dehydrogenase / alcohol dehydrogenase;  S-(hydroxymethyl)glutathione dehydrogenase / alcohol dehydrogenase FrmA;  S-(hydroxymethyl)glutathione dehydrogenase/class III alcohol dehydrogenase;  S-(hydroxymethyl)glutathione dehydrogenase/class III alcohol dehydrogenase, partial;  Short chain alcohol dehydrogenase-like protein;  Short-chain alcohol dehydrogenase;  Zinc-binding alcohol dehydrogenase;  Zinc-binding alcohol dehydrogenase family protein;  Zinc-containing alcohol dehydrogenase;  Zinc-containing alcohol dehydrogenase family protein;  Zinc-containing alcohol dehydrogenase superfamily;  Zinc-containing alcohol dehydrogenase superfamily protein;  Zinc-containing alcohol dehydrogenase superfamily protein, partial;  Zinc-dependent alcohol dehydrogenase;  Zinc-dependent alcohol dehydrogenase, partial;  Zinc-type alcohol dehydrogenase-like protein;  Zinc-type alcohol dehydrogenase-like protein C1773.06c;  Zn-dependent alcohol dehydrogenase;  Zn-dependent alcohol dehydrogenase, class III. | *Pseudomonas stutzeri*: 70/954  *Limnobacter* sp. MED105: 29/204  *Limnobacter* sp. CACIAM 66H1: 17/151  *Thiobacillus denitrificans*: 12/17  *Mesorhizobium* sp. F7: 10/23 | *Pseudomonas*: 142/1219  *Limnobacter*: 38/356  *Bradyrhizobium*: 37/147  *Microbacterium*: 29/161  *Nitrospira*: 24/36 |
| Aldehyde dehydrogenase | | 1068 | | 3146 | Aldehyde dehydrogenase;  Aldehyde dehydrogenase (acceptor);  Aldehyde dehydrogenase (NAD(+));  Aldehyde dehydrogenase (NAD);  Aldehyde dehydrogenase (NAD) family protein;  Aldehyde dehydrogenase (NAD) family protein, partial;  Aldehyde dehydrogenase (NAD+);  Aldehyde dehydrogenase (NADP(+));  Aldehyde dehydrogenase (NADP(+)), partial;  Aldehyde dehydrogenase (nadp) family protein;  Aldehyde dehydrogenase (plasmid);  Aldehyde dehydrogenase AldH;  Aldehyde dehydrogenase B;  Aldehyde dehydrogenase B (wide specificity);  Aldehyde dehydrogenase DhaS;  Aldehyde dehydrogenase domain protein;  Aldehyde dehydrogenase family;  Aldehyde dehydrogenase family protein;  Aldehyde dehydrogenase family protein, partial;  Aldehyde dehydrogenase family, partial;  Aldehyde dehydrogenase iron-sulfur subunit;  Aldehyde dehydrogenase precursor;  Aldehyde dehydrogenase PuuC;  Aldehyde dehydrogenase subunit III;  Aldehyde dehydrogenase, 1-pyrroline-5-carboxylate dehydrogenase;  Aldehyde Dehydrogenase, aldehyde dehydrogenase (NAD+);  Aldehyde dehydrogenase, partial;  Aldehyde dehydrogenase, putative;  Aldehyde dehydrogenase, thermostable;  Bifunctional aldehyde dehydrogenase/enoyl-CoA hydratase;  Coniferyl aldehyde dehydrogenase;  Coniferyl aldehyde dehydrogenase, partial;  Coniferyl-aldehyde dehydrogenase;  EPTC-inducible aldehyde dehydrogenase;  MULTISPECIES: aldehyde dehydrogenase;  MULTISPECIES: coniferyl aldehyde dehydrogenase;  MULTISPECIES: coniferyl-aldehyde dehydrogenase;  NAD-dependent aldehyde dehydrogenase;  NAD-dependent aldehyde dehydrogenase (plasmid);  NAD-dependent aldehyde dehydrogenase, partial;  NADP-dependent fatty aldehyde dehydrogenase;  NADP-dependent fatty aldehyde dehydrogenase, partial;  Phenylacetic acid degradation protein PaaN, ring-opening aldehyde dehydrogenase;  Probable aldehyde dehydrogenase;  Putative aldehyde dehydrogenase;  Putative aldehyde dehydrogenase (plasmid);  Putative aldehyde dehydrogenase AldA;  Putative aldehyde dehydrogenase precursor (tat pathway signal);  Putative aldehyde dehydrogenase protein;  Putative coniferyl aldehyde dehydrogenase;  Putative transmembrane aldehyde dehydrogenase oxidoreductase protein. | *Pseudomonas stutzeri*: 30/344  *Limnobacter* sp. MED105: 20/239  *Pseudomonas aeruginosa*: 15/63  *Microcella alkaliphila*: 14/306  *Thermobaculum terrenum*: 12/13 | *Pseudomonas*: 106/760  *Bradyrhizobium*: 53/151  *Sphingopyxis*: 50/80  *Microbacterium*: 32/125  *Limnobacter*: 25/331 |
| Aldehyde oxidase | | 142 | | 365 | Aldehyde oxidase;  Aldehyde oxidase and xanthine dehydrogenase;  Aldehyde oxidase and xanthine dehydrogenase molybdopterin binding;  Aldehyde oxidase and xanthine dehydrogenase molybdopterin binding protein;  Aldehyde oxidase and xanthine dehydrogenase molybdopterin binding protein (plasmid);  Aldehyde oxidase and xanthine dehydrogenase molybdopterin-binding protein;  Aldehyde oxidase and xanthine dehydrogenase, a/b hammerhead domain;  Aldehyde oxidase and xanthine dehydrogenase, molybdopterin binding;  Aldehyde oxidase and xanthine dehydrogenase, molybdopterin binding protein;  Aldehyde oxidase, partial;  Molybdopterin binding aldehyde oxidase;  Molybdopterin binding aldehyde oxidase and xanthine dehydrogenase;  Molybdopterin binding aldehyde oxidase and xanthine dehydrogenase protein;  Molybdopterin-binding aldehyde oxidase and xanthine dehydrogenase;  Molybdopterin-binding aldehyde oxidase andxanthine dehydrogenase;  MULTISPECIES: aldehyde oxidase. | *Methyloversatilis universalis*: 9/45  *Gemmatirosa kalamazoonesis*: 7/19  *Methyloversatilis discipulorum*: 6/7  *Pseudomonas* *xanthomarina*: 5/49  *Pseudomonas* sp. BAY1663: 5/5 | *Methyloversatilis*: 20/59  *Pseudomonas*: 16/105  *Bradyrhizobium*: 10/30  *Gemmatirosa*: 7/19  *Skermanella*: 5/5 |
| Alkanal monooxygenase | | 52 | | 71 | Alkanal monooxygenase;  Alkanal monooxygenase alpha chain;  Alkanal monooxygenase subunit alpha;  Flavin-dependent alkanal monooxygenase;  Luciferase-like monooxygenase;  Luciferase-like monooxygenase family protein;  Luciferase-like protein monooxygenase;  Putative alkanal monooxygenase. | *Rhodoplanes* sp. Z2-YC6860: 17/17  *Pseudomonas* *syringae*: 5/7  *Microcella* *alkaliphila*: 5/6  *Arthrobacter crystallopoietes*: 3/3  *Variovorax paradoxus*: 2/2 | *Rhodoplanes*: 17/17  *Pseudomonas*: 5/7  *Microcella*: 5/6  *Arthrobacter*: 3/3  *Variovorax*: 2/2 |
| Alkylhydroperoxidase | | 76 | | 137 | Alkylhydroperoxidase | *Pseudomonas stutzeri*: 9/24  *Ralstonia pickettii*: 8/9  *Pseudomonas* *chloritidismutans*: 6/ 17  *Methylocapsa aurea*: 6/6  *Methylobacterium* sp. Leaf90: 2/3 | *Pseudomonas*: 16/44  *Ralstonia*: 8/ 9  *Methylocapsa*: 6/6  *Bradyrhizobium*: 4/17  *Methylobacterium*: 2/3 |
| Aryl-alcohol dehydrogenase | | 15 | | 24 | Aryl-alcohol dehydrogenase;  Aryl-alcohol dehydrogenase (NADP(+));  NADP-dependent aryl-alcohol dehydrogenase;  Oxidoreductase (related to aryl-alcohol dehydrogenase);  Oxidoreductase aryl-alcohol dehydrogenase like protein;  Putative oxidoreductase, aryl-alcohol dehydrogenase like protein. | *Arthrobacter nitrophenolicus*: 3/9  *Rhizobium* sp. CF080: 2/2  *Streptomyces bingchenggensis*: 1/1  *Pleurocapsa minor*: 1/1  *Opitutus terrae*: 1/1 | *Arthrobacter*: 3/9  *Rhizobium*: 2/2  *Streptomyces*:1/1  *Pleurocapsa*: 1/1  *Opitutus*: 1/1 |
| Baeyer-Villiger monooxygenase | | 48 | | 161 | Baeyer-Villiger monooxygenase;  FAD-containing monooxygenase EthA;  Phenylacetone monooxygenase. | *Limnobacter* sp. CACIAM 66H1: 7/70  *Erythrobacter* sp. AP23: 3/4  *Altererythrobacter marensis*: 3/3  *Reyranella massiliensis*: 3/3  *Bradyrhizobium* sp. CCH5-F6: 2/7 | *Bradyrhizobium*: 9/15  *Limnobacter* sp.: 7/70  *Erythrobacter*: 6/8  *Altererythrobacter*: 5/5  *Reyranella*: 3/3 |
| Butanol dehydrogenase | | 3 | | 5 | Butanol dehydrogenase | *Sedimenticola selenatireducens*: 3/4 | *Sedimenticola*: 3/4 |
| Carboxymuconolactone decarboxylase | | 113 | | 270 | 4-Carboxymuconolactone decarboxylase;  4-Carboxymuconolactone decarboxylase (plasmid);  4-Carboxymuconolactone decarboxylase, partial;  Carboxymuconolactone decarboxylase;  Carboxymuconolactone decarboxylase domain protein;  Carboxymuconolactone decarboxylase family protein;  Gamma-carboxymuconolactone decarboxylase;  MULTISPECIES: 4-carboxymuconolactone decarboxylase;  MULTISPECIES: carboxymuconolactone decarboxylase;  Uncharacterized protein, gamma-carboxymuconolactone decarboxylase subunit like protein. | *Pseudomonas stutzeri*: 8/47  *Sulfurifustis variabilis*: 8/8  *Sulfuricaulis* *limicola*: 7/60  *Limnobacter* sp. MED105: 7/32  *Limnobacter* sp. CACIAM 66H1: 5/5 | *Limnobacter*: 12/38  *Bradyrhizobium*: 12/24  *Pseudomonas*: 9/55  *Sulfurifustis*: 8/8  *Sulfuricaulis*: 7/60 |
| Cyclohexanone monooxygenase | | 129 | | 374 | Cyclohexanone 1,2-monooxygenase;  Cyclohexanone monooxygenase;  Cyclohexanone monooxygenase EC 1141322 CDS;  Cyclohexanone monooxygenase, partial;  MULTISPECIES: cyclohexanone monooxygenase;  Putative (cyclohexanone) monooxygenase. | *Microbacterium ginsengisoli*: 9/188  *Sorangium cellulosum*: 9/12  *Arthrobacter* sp. SPG23: 8/10  *Geodermatophilus* sp. Leaf369: 7/7  *Pseudonocardia autotrophica*: 6/7 | *Bradyrhizobium*: 15/22  *Pseudonocardia*: 12/13  *Microbacterium*: 10/194  *Arthrobacter*: 10/12  *Sorangium*: 9/12 |
| Cyclopentanone 1,2-monooxygenase | | 15 | | 26 | Cyclopentanone 1,2-monooxygenase;  MULTISPECIES: cyclopentanone 1,2-monooxygenase. | *Sphingopyxis fribergensis*: 4/6  *Sphingopyxis* sp. A083: 2/2  *Sphingopyxis terrae*: 1/1  *Caulobacter* sp. K31: 1/1 | *Sphingopyxis*: 12/19  *Caulobacter*: 1/1  *Sphingomonas*: 1/1 |
| Cytochrome P450 | | 309 | | 761 | Cytochrome P450;  Cytochrome P450 (plasmid);  Cytochrome P450 187A4 Cyp187A4;  Cytochrome P450 187A5 Cyp187A5;  Cytochrome P450 alkane hydroxylase, partial;  Cytochrome P450 CYP124E1;  Cytochrome P450 CYP153 alkane hydroxylase, partial;  Cytochrome P450 CYP153, partial;  Cytochrome P450 CYP153A, partial;  Cytochrome P450 family protein;  Cytochrome P450 hydroxylase;  Cytochrome P450 hydroxylase (monooxygenase);  Cytochrome P450 steroid C27-monooxygenase;  Cytochrome P450 superfamily;  Cytochrome P450 superfamily protein;  Cytochrome P450, partial;  MULTISPECIES: cytochrome P450;  MULTISPECIES: NADPH--cytochrome P450 reductase;  NADPH--cytochrome P450 reductase;  Putative cytochrome P450;  Putative cytochrome P450 120;  Putative cytochrome P450 152A1;  Putative cytochrome P450 hydroxylase;  Putative cytochrome P450 hydroxylase CDS;  Putative cytochrome P450 protein. | *Microvirga vignae*: 8/8  *Limnobacter* sp. MED105: 7/69  *Sphingopyxis* sp. LC363: 7/12  *Actinotalea ferrariae*: 6/6  *Bradyrhizobium japonicum*: 5/31 | *Bradyrhizobium*: 37/164  *Sphingopyxis*: 20/38  *Microbacterium*: 11/21  *Pseudomonas*: 8/41  *Limnobacter*: 7/69 |
| Dienelactone hydrolase | | 189 | | 391 | Dienelactone hydrolase;  Dienelactone hydrolase family protein;  Dienelactone hydrolase, carboxymethylenebutenolidase;  Dienelactone hydrolase, partial;  Dienelactone hydrolase-like enzyme;  Dienelactone hydrolase-like protein enzyme;  MULTISPECIES: dienelactone hydrolase;  Predicted dienelactone hydrolase;  Putative dienelactone hydrolase (plasmid). | *Thiobacillus* *denitrificans*: 20/59  *Pseudomonas stutzeri*: 16/59  *Thiobacillus thioparus*: 13/15  *Methylomarinum vadi*: 5/5  *Thioalkalivibrio thiocyanodenitrificans*: 4/4  *Sulfurifustis variabilis*: 3/13 | *Thiobacillus*: 33/74  *Pseudomonas*: 22/88  *Thioalkalivibrio*: 7/7  *Nitrosospira*: 6/6  *Novosphingobium*: 5/6 |
| Dioxygenase  (metabolism of aromatic compounds) | | 880 | | 2084 | 2-(2,4-Dichlorophenoxy)propionate,2-oxoglutarate dioxygenase, partial;  2,3-Dihydroxybiphenyl 1,2-dioxygenase;  2,3-Dihydroxybiphenyl dioxygenase;  2,4-Dichlorophenoxyacetate dioxygenase;  2,4-Dihydroxyacetophenone dioxygenase;  3-(2,3-Dihydroxyphenyl)propionate dioxygenase;  3,4-Dihydroxyphenylacetate 2,3-dioxygenase;  3,4-Dioxygenase subunit beta;  3-Chlorobenzoate-3,4-dioxygenase dehydrogenase;  3-Chlorobenzoate-3,4-dioxygenase oxygenase subunit;  3-Phenylpropionate dioxygenase ferredoxin subunit;  3-Phenylpropionate dioxygenase subunit alpha;  3-Phenylpropionate dioxygenase;  4,5-Dioxygenase;  6-Chlorohydroxyquinol-1,2-dioxygenase;  Aromatic 1,2-dioxygenase large subunit;  Aromatic 1,2-dioxygenase small subunit;  Aromatic ring dioxygenase beta subunit;  Aromatic ring-hydroxylating dioxygenase alpha subunit, partial;  Aromatic ring-hydroxylating dioxygenase subunit alpha;  Aromatic ring-opening dioxygenase LigA;  Aromatic-ring-hydroxylating dioxygenase subunit alpha;  Aromatic-ring-hydroxylating dioxygenase subunit beta;  Aromatic-ring-hydroxylating dioxygenase;  Benzene 1,2-dioxygenase system ferredoxin subunit;  Benzene 1,2-dioxygenase;  Benzoate 1,2-dioxygenase ferredoxin reductase subunit;  Benzoate 1,2-dioxygenase hydroxylase component beta subunit;  Benzoate 1,2-dioxygenase large subunit;  Benzoate 1,2-dioxygenase small subunit;  Benzoate 1,2-dioxygenase subunit alpha;  Benzoate dioxygenase, alpha subunit;  Benzoate dioxygenase, ferredoxin reductase component;  Bifunctional 3-phenylpropionate/cinnamic acid dioxygenase ferredoxin subunit;  Biphenyl 2,3-dioxygenase;  Carbazole dioxygenase;  Catalytic LigB subunit of aromatic ring-opening dioxygenase;  Catechol 1,2-dioxygenase, partial;  Catechol 1,2-dioxygenase;  Catechol 2,3 dioxygenase;  Catechol 2,3-dioxygenase (plasmid);  Catechol 2,3-dioxygenase, partial;  Catechol 2,3-dioxygenase;  Catechol-2,3-dioxygenase;  Dioxygenase ferredoxin subunit;  Dioxygenase hydroxylase;  Dioxygenase large subunit;  Dioxygenase subunit alpha YeaW;  Dioxygenase, partial;  Dioxygenase;  Dioxygenase-like protein;  Extradiol dioxygenase;  Extradiol ring-cleavage dioxygenase class III protein subunit B;  Extradiol ring-cleavage dioxygenase III subunit B;  Extradiol ring-cleavage dioxygenase;  Fe2+-dependent dioxygenase;  Homogentisate 1,2-dioxygenase, partial;  Homogentisate 1,2-dioxygenase;  Homogentisate 12-dioxygenase;  Hydroxyquinol 1,2-dioxygenase;  Intradiol ring-cleavage dioxygenase;  IPB-dioxygenase, ISP large subunit (IpbA1);  LigB family aromatic ring-opening extradiol dioxygenase;  LigB family dioxygenase;  Lignostilbene alpha-beta-dioxygenase;  MULTISPECIES: 2,3-dihydroxybiphenyl 1,2-dioxygenase;  MULTISPECIES: 2-chlorobenzoate 1,2-dioxygenase;  MULTISPECIES: 3,4-dihydroxyphenylacetate 2,3-dioxygenase;  MULTISPECIES: 3-phenylpropionate dioxygenase;  MULTISPECIES: alpha-ketoglutarate-dependent 2,4-dichlorophenoxyacetate dioxygenase;  MULTISPECIES: anthranilate 1,2-dioxygenase small subunit;  MULTISPECIES: benzene 1,2-dioxygenase;  MULTISPECIES: benzoate 1,2-dioxygenase small subunit;  MULTISPECIES: biphenyl 2,3-dioxygenase;  MULTISPECIES: dioxygenase;  MULTISPECIES: extradiol dioxygenase;  MULTISPECIES: glyoxalase/bleomycin resistance protein/dioxygenase;  MULTISPECIES: homogentisate 1,2-dioxygenase;  MULTISPECIES: p-cumate dioxygenase;  MULTISPECIES: protocatechuate 3,4-dioxygenase subunit beta;  MULTISPECIES: protocatechuate 4,5-dioxygenase subunit alpha;  MULTISPECIES: ring-cleavage extradiol dioxygenase;  Naphthalene 1,2-dioxygenase/salicylate 5-hydroxylase system;  Naphthalene 1,2-dioxygenase;  p-Cumate dioxygenase large subunit (CmtAb);  p-Cumate dioxygenase;  Phenoxybenzoate dioxygenase subunit alpha;  Phenoxybenzoate dioxygenase subunit beta;  Phenylpropionate dioxygenase;  Phthalate 4,5-dioxygenase oxygenase subunit;  Phthalate 4,5-dioxygenase;  Protocatechuate 3,4-dioxygenase alpha chain;  Protocatechuate 3,4-dioxygenase beta chain;  Protocatechuate 3,4-dioxygenase beta subunit, partial;  Protocatechuate 3,4-dioxygenase b-subunit, partial;  Protocatechuate 3,4-dioxygenase subunit alpha;  Protocatechuate 3,4-dioxygenase subunit beta;  Protocatechuate 3,4-dioxygenase;  Protocatechuate 4,5-dioxygenase beta chain;  Protocatechuate 4,5-dioxygenase subunit alpha;  Protocatechuate 4,5-dioxygenase subunit beta;  Putative aromatic ring hydroxylating dioxygenase, partial;  Putative dioxygenase subunit;  Putative dioxygenase;  Putative oxoglutarate/iron-dependent dioxygenase;  Putative quercetin 2,3-dioxygenase;  Putative ring hydroxylating dioxygenase large subunit, partial;  Quercetin 2,3-dioxygenase sll1773;  Quercetin 2,3-dioxygenase;  Rieske (2Fe-2S) iron-sulfur domain-containing protein, dioxygenase ferredoxin subunit;  Ring hydroxylating dioxygenase subunit alpha;  Ring-cleavage extradiol dioxygenase;  Ring-cleaving dioxygenase;  Ring-hydroxylating dioxygenase large subunit;  Ring-hydroxylating dioxygenase subunit beta;  Ring-hydroxylating dioxygenase, large terminal subunit;  Ring-hydroxylating dioxygenase, partial;  Ring-hydroxylating dioxygenase;  Terephthalate 1,2-dioxygenase;  Terminal dioxygenase component beta subunit. | *Pseudomonas stutzeri*: 38/316  *Yonghaparkia* sp. Soil809: 18/61  *Limnobacter* sp. MED105: 16/30  *Limnobacter* sp. CACIAM 66H1: 15/75  *Sulfuricaulis limicola*: 10/51 | *Bradyrhizobium*: 73/149  *Pseudomonas*: 67/450  *Microbacterium*: 31/202  *Sphingopyxis*: 29/44  *Yonghaparkia*: 21/71 |
| Dioxygenase  (metabolism of aliphatic compounds) | | 215 | | 507 | 2-Nitropropane dioxygenase;  2-Nitropropane dioxygenase, NPD;  MULTISPECIES: 2-nitropropane dioxygenase;  NAD(P)H-dependent flavin oxidoreductase YrpB, nitropropane dioxygenase family;  Oxidoreductase, 2-nitropropane dioxygenase family protein;  Putative 2-nitropropane dioxygenase. | *Sulfuricaulis limicola*: 8/92  *Pseudomonas stutzeri*: 8/23  *Limnobacter sp.* CACIAM 66H1: 7/30  *Thiobacillus denitrificans*: 6/37  *Sphingopyxis* sp. LC363: 6/9 | *Bradyrhizobium*: 17/26  *Sphingopyxis*: 15/25  *Thiobacillus*: 12/45  *Pseudomonas*: 11/36  *Limnobacter*: 9/51 |
| Ethanol dehydrogenase | | 12 | | 125 | Methanol/ethanol family PQQ-dependent dehydrogenase;  PQQ-dependent dehydrogenase, methanol/ethanol family;  Putative quinoprotein ethanol dehydrogenase;  Quinohemoprotein ethanol dehydrogenase;  Quinohemoprotein ethanol dehydrogenase type-1;  Quinohemoprotein ethanol dehydrogenase type-1 precursor;  Quinoprotein ethanol dehydrogenase;  Quinoprotein ethanol dehydrogenase precursor. | *Pseudomonas* *chloritidismutans*: 3/97  *Schlesneria paludicola*: 3/3  *Hydrogenophaga* sp. T4: 2/4  *Streptococcus pneumoniae*: 1/5  *Pseudomonas caeni*: 1/3 | *Pseudomonas*: 4/103  *Schlesneria paludicola*: 3/3  *Hydrogenophaga* sp. T4: 2/4  *Streptococcus*: 1/5  *Bradyrhizobium*: 1/3 |
| Glucose-methanol-choline oxidoreductase | | 53 | | 85 | Glucose-methanol-choline (GMC) oxidoreductase:NAD binding protein site;  Glucose-methanol-choline (GMC) oxidoreductase:NAD binding site;  Glucose-methanol-choline oxidoreductase family;  Glucose-methanol-choline oxidoreductase;  MULTISPECIES: glucose-methanol-choline oxidoreductase. | *Afipia* sp. Root123D2: 3/3  *Sphingopyxis macrogoltabida*: 2/2  *Filimonas lacunae*: 2/2  *Ralstonia* sp. PBA: 2/2  *Oceanicaulis* sp. HL-87: 2/2 | *Sphingopyxis*: 10/19  *Bradyrhizobium*: 3/14  *Afipia*: 3/3  *Burkholderia*: 2/4  *Ralstonia*: 2/2 |
| Haloalkane dehalogenase | | 19 | | 26 | Haloalkane dehalogenase;  MULTISPECIES: haloalkane dehalogenase. | *Caulobacter* sp. OV484: 3/3  *Nocardioides* sp. URHA0020: 2/2  *Marinobacter algicola*: 2/2  *Shewanella frigidimarina*: 1/1  *Hyphomonas adhaerens*: 1/1 | *Marinobacter*: 4/5  *Caulobacter*: 3/3  *Nocardioides*: 2/2  *Shewanella*: 1/1  *Hyphomonas*: 1/1 |
| Haloacid dehalogenase | | 470 | | 1155 | D-2-Haloacid dehalogenase;  Haloacid dehalogenase;  Haloacid dehalogenase (plasmid);  Haloacid dehalogenase domain protein hydrolase;  Haloacid dehalogenase superfamily enzyme, subfamily IA;  Haloacid dehalogenase, partial;  Haloacid dehalogenase, subfamily IA;  Haloacid dehalogenase, subfamily IA, Putative hydrolase of the HAD superfamily;  Haloacid dehalogenase, type II;  Haloacid dehalogenase-like hydrolase;  Hydrolase, haloacid dehalogenase-like family;  Hydrolase, haloacid dehalogenase-like family protein;  MULTISPECIES: haloacid dehalogenase;  Putative haloacid dehalogenase family hydrolase;  Putative haloacid dehalogenase superfamily hydrolase, subfamily IB, PSPase-like. | *Thiobacillus denitrificans*: 21/  *Pseudomonas stutzeri*: 18/244  *Limnobacter* sp. MED105: 16/62  *Limnobacter* sp. CACIAM 66H1: 11/52  *Sulfuricaulis* *limicola*: 9/90 | *Bradyrhizobium*: 45/82  *Thiobacillus*: 29/65  *Limnobacter*: 27/114  *Pseudomonas*: 24/267  *Yonghaparkia*: 13/42 |
| Hydroxylase  (aromatic compounds) | | 149 | | 310 | 3-(3-Hydroxyphenyl)propionate hydroxylase;  4-Hydroxyphenylacetate 3-hydroxylase;  Aromatic ring hydroxylase;  Methane/phenol/toluene hydroxylase;  MULTISPECIES: phenol hydroxylase;  Phenol hydroxylase;  Phenol hydroxylase, putative;  P-Hydroxybenzoate hydroxylase;  Putative 2-octaprenyl-3-methyl-6-methoxy-1, 4-benzoquinol hydroxylase;  Salicylate hydroxylase;  Salicylate hydroxylase, partial;  Salicylyl-CoA 5-hydroxylase. | *Pseudonocardia dioxanivorans*: 7/7  *Rhodoplanes* sp. Z2-YC6860: 7/7  *Sphingomonas* sp. URHD0057: 6/6  *Limnobacter* sp. MED105: 4/57  *Limnobacter* sp. CACIAM 66H1: 4/25 | *Sphingomonas*: 16/17  *Bradyrhizobium*: 14/48  *Pseudonocardia*: 11/11  *Limnobacter*: 8/82 |
| Isoquinoline 1-oxidoreductase | | 95 | | 153 | Isoquinoline 1-oxidoreductase;  Isoquinoline 1-oxidoreductase alpha subunit protein;  Isoquinoline 1-oxidoreductase beta subunit;  isoquinoline 1-oxidoreductase subunit alpha;  Isoquinoline 1-oxidoreductase subunit beta;  isoquinoline 1-oxidoreductase, alpha subunit;  isoquinoline 1-oxidoreductase, partial;  MULTISPECIES: isoquinoline 1-oxidoreductase;  Putative Isoquinoline 1-oxidoreductase beta subunit, iorB-like;  Putative isoquinoline 1-oxidoreductase, beta subunit. | *Rhodoplanes* sp. Z2-YC6860: 6/6  *Novosphingobium malaysiense*: 5/6  *Niastella koreensis*: 5/5  *Azoarcus* sp. KH32C: 3/15  *Inquilinus limosus*: 3/3 | *Novosphingobium*: 6/7  *Rhodoplanes*: 6/6  *Algoriphagus*: 6/6  *Niastella*: 5/5  *Azoarcus* sp.: 3/15 |
| Laccase | | 51 | | 140 | Laccase;  Laccase, partial;  Laccase domain protein YfiH;  Putative laccase, partial. | *Sulfuricaulis limicola*: 8/38  *Pseudomonas* sp. BRH_c35: 2/13  *Haliea salexigens*: 2/2  *Pseudomonas stutzeri*: 1/18  *Pseudomonas chlororaphis*: 1/1 | *Sulfuricaulis*: 8/38  *Pseudomonas*: 5/58  *Haliea*: 2/2  *Sulfurifustis*: 1/1  *Methylomonas*: 1/1 |
| Methane monooxygenase | | 13 | | 25 | Methane monooxygenase;  Methane monooxygenase A subunit;  Methane monooxygenase/ammonia monooxygenase subunit A;  Methane monooxygenase/ammonia monooxygenase subunit C;  Methane monooxygenase/ammonia monooxygenase subunit C, partial;  Particulate methane monooxygenase alpha subunit precursor;  Particulate methane monooxygenase beta subunit;  Putative methane monooxygenase, B subunit;  Soluble methane monooxygenase, partial. | *Hydrogenophaga* sp. T4: 3/3  *Mycobacterium rhodesiae*: 1/1  *Nocardioides luteus*: 1/1  *Solirubrobacter* sp. URHD0082: 1/1 | *Hydrogenophaga*: 3/3  *Mycobacterium*: 1/1  *Nocardioides*: 1/1  *Solirubrobacter*: 1/1 |
| Monooxygenase  (metabolism of aromatic compounds) | | 155 | | 462 | 2,4-Dichlorophenol 6-monooxygenase;  4-Hydroxyacetophenone monooxygenase;  4-Hydroxybenzoate 3-monooxygenase;  4-Hydroxyphenylacetate 3-monooxygenase large subuit;  4-Hydroxyphenylacetate 3-monooxygenase, oxygenase component;  4-Hydroxyphenylacetate 3-monooxygenase;  Anthraniloyl-CoA monooxygenase;  Dimethylaniline monooxygenase (N-oxide forming);  Dimethylaniline monooxygenase;  HpaB-1: 4-hydroxyphenylacetate 3-monooxygenase, oxygenase, partial;  MULTISPECIES: 4-hydroxybenzoate 3-monooxygenase;  MULTISPECIES: phenol 2-monooxygenase;  Pentachlorophenol 4-monooxygenase;  Phenol 2-monooxygenase;  p-Hydroxybenzoate hydroxylase, FAD dependent monooxygenase;  Salicylate 1-monooxygenase;  Styrene monooxygenase subunit A;  Toluene 4-monooxygenase A;  Toluene monooxygenase;  Toluene-4-monooxygenase system protein A;  Toluene-4-monooxygenase system protein E. | *Limnobacter* sp. MED105: 9/141  *Bradyrhizobium* sp. Leaf396: 9/20  *Thiolapillus brandeum*: 8/8  *Limnobacter* sp. CACIAM 66H1: 6/87  *Pseudonocardia acaciae*: 4/4 | *Bradyrhizobium*: 27/63  *Limnobacter*: 15/228  *Microbacterium*: 11/15  *Lysinimicrobium*: 5/5  *Marinobacter*: 4/5 |
| Monooxygenase  (metabolism of aliphatic compounds) | | 430 | | 1163 | Alkane 1-monooxygenase, partial;  Alkane 1-monooxygenase;  Alkane monooxygenase, partial;  Alkanesulfonate monooxygenase;  Alpha subunit monooxygenase protein, partial;  Copper type II ascorbate-dependent monooxygenase;  FAD-binding monooxygenase;  Favin-binding family monooxygenase;  Flavin binding monooxygenase;  Flavin-binding monooxygenase;  Flavin-binding monooxygenase-like protein;  Flavin-binding monooxygenase-like subfamily;  Flavin-containing monooxygenase FMO;  Flavin-containing monooxygenase;  Flavin-dependent oxidoreductase, luciferase family (includes alkanesulfonate monooxygenase SsuD and methylene tetrahydromethanopterin reductase);  Flavoprotein monooxygenase;  FMNH2-dependent monooxygenase;  Monooxygenase FAD-binding protein;  Monooxygenase FAD-binding;  Monooxygenase flavin-binding family protein;  Monooxygenase protein;  Monooxygenase, FAD-binding;  Monooxygenase, flavin-binding family protein;  Monooxygenase, flavin-binding family;  Monooxygenase, partial;  Monooxygenase;  MULTISPECIES: alkane 1-monooxygenase;  MULTISPECIES: monooxygenase;  MULTISPECIES: Putative flavin-containing monooxygenase;  Nitrilotriacetate monooxygenase component A (NTA monooxygenase component A) (NTA-MO A);  Nitronate monooxygenase;  Probable monooxygenase;  Putative alkene monooxygenase reductase;  Putative flavin-binding monooxygenase, partial;  Putative monooxygenase, partial;  Putative monooxygenase;  Putative propane monooxygenase beta subunit;  Soluble di-iron monooxygenase alpha subunit, partial;  Xenobiotic compound monooxygenase, DszA family. | *Reyranella massiliensis*: 14/14  *Bradyrhizobium* sp. Leaf396: 11/40  *Tepidicaulis marinus*: 10/10  *Caulobacter* sp. K31: 8/8  *Rathayibacter tritici*: 6/6 | *Bradyrhizobium*: 80/245  *Leifsonia*: 14/85  *Sphingopyxis*: 14/22  *Mycobacterium*: 10/16  *Pseudomonas*: 7/19 |
| Oxygenase  (metabolism of aromatic compounds) | | 60 | | 121 | Benzoyl-CoA oxygenase component B;  Benzoyl-CoA oxygenase subunit B;  Benzoyl-CoA oxygenase, B subunit;  Benzoyl-CoA oxygenase, component B;  Benzoyl-CoA oxygenase/reductase, BoxA protein;  Benzoyl-CoA oxygenase;  MULTISPECIES: benzoyl-CoA oxygenase subunit B;  Ring-hydroxylating oxygenase subunit alpha. | *Haliangium ochraceum*: 5/5  *Pseudolabrys* sp. Root1462: 4/4  *Novosphingobium* sp. B-7: 3/3  *Reyranella massiliensis*: 3/3  *Bradyrhizobium* sp. Leaf396: 2/14 | *Bradyrhizobium*: 6/36  *Haliangium*: 5/5  *Pseudolabrys*: 4/4  *Afipia*: 3/8  *Novosphingobium*: 3/3 |
| Phytanoyl-CoA dioxygenase | | 67 | | 104 | MULTISPECIES: phytanoyl-CoA dioxygenase;  Phytanoyl-CoA dioxygenase;  Phytanoyl-CoA dioxygenase (PhyH);  Phytanoyl-CoA dioxygenase 1;  Phytanoyl-CoA dioxygenase family protein;  Phytanoyl-CoA dioxygenase, partial. | *Schlesneria paludicola* 4/4  *Sphingopyxis granuli*: 2/3  *Belnapia moabensis*: 2/2  *Planctomyces* sp. SH-PL14: 2/2  *Planctopirus limnophila*: 2/2 | *Sphingopyxis*: 9/17  *Schlesneria*: 4/4  *Belnapia*: 2/2  *Planctopirus*: 2/2  *Bradyrhizobium*: 1/16 |
| **OXIDATIVE METABOLISM OF INORGANIC SULFUR COMPOUNDS** | | | | | | | |
| Adenylylsulfate: phosphate adenylyltransferase | 1 | | 1 | | Adenylylsulfate:phosphate adenylyltransferase | *Thiobacillus denitrificans:* 1/14 | *Thiobacillus :* 1/14 |
| Adenylylsulfate reductase | 160 | | 783 | | Adenylylsulfate reductase alpha subunit, partial;  Adenylylsulfate reductase alpha subunit;  Adenylylsulfate reductase beta subunit, partial;  Adenylylsulfate reductase subunit alpha, adenylylsulfate reductase, subunit A;  Adenylylsulfate reductase subunit alpha;  Adenylylsulfate reductase subunit beta;  Adenylylsulfate reductase, beta subunit;  Adenylylsulfate reductase, membrane anchor;  Adenylylsulfate reductase, partial;  Adenylylsulfate reductase, subunit B;  Adenylylsulfate reductase;  Dissimilatory adenylylsulfate reductase subunit alpha precursor;  Putative 5\'-adenylylsulfate reductase, thioredoxin-independent;  Putative 5\'-adenylylsulfate reductase;  Putative adenylylsulfate reductase, subunit A. | *Sulfuricaulis* *limicola*: 27/ 217  *Thiobacillus* *denitrificans*: 27/95  *Sulfurifustis* *variabilis*: 11/61  *Candidatus Gallionella acididurans*: 8/12  *Thiobacillus thioparus*: 7/32 | *Thiobacillus*: 34/128  *Sulfuricaulis*: 27/217  *Sulfurifustis*: 11/ 61  *Gallionella*: 8/12  *Sedimenticola*: 6/13 |
| Flavocytochrome c sulfide dehydrogenase | 6 | | 6 | | Flavocytochrome C sulfide dehydrogenase | *Thiobacillus denitrificans*: 6/6 | *Thiobacillus*: 6/6 |
| Reverse-type dissimilatory sulfite reductase | 26 | | 71 | | Reverse dissimilatory sulfite reductase alpha subunit, partial;  Reverse dissimilatory sulfite reductase beta subunit, partial;  Reverse-type dissimilatory siroheme sulfite reductase subunit A, partial;  Reverse-type dissimilatory siroheme sulfite reductase subunit B, partial;  Reverse-type dissimilatory sulfite reductase (rDSR), alpha subunit (DsrA);  Reverse-type dissimilatory sulfite reductase (rDSR), beta subunit (DsrB). | *Thiobacillus thioparus*: 2/4 | *Thiobacillus*: 2/4 |
| SoxA | 84 | | 266 | | Sulfur oxidation c-type cytochrome SoxA;  Sulfur oxidation protein SoxA. | *Thiobacillus denitrificans:* 16/59  *Bradyrhizobium* sp. Leaf396: 10/20  *Sulfuricaulis* *limicola*: 9/73  *Thiothrix disciformis*: 7/7  *Limnobacter* sp. MED105: 6/49 | *Thiobacillus*: 19/62  *Bradyrhizobium:* 17/40  *Limnobacter:* 10/54  *Sulfuricaulis*: 9/ 73  *Thiothrix*: 7/7 |
| SoxB | 161 | | 448 | | SoxB, partial;  Sulfur oxidation protein SoxB;  Sulfur/thiosulfate oxidation protein SoxB;  Thiosulfohydrolase SoxB. | *Thiobacillus denitrificans*: 14/58  *Limnobacter* sp. CACIAM 66H1: 9/61  *Polaromonas* sp. EUR3 1.2.1: 7/48  *Thiobacillus thioparus*: 7/10  *Limnobacter* sp. MED105: 5/30 | *Thiobacillus*: 21/68  *Limnobacter*: 14/91  *Bradyrhizobium*: 12/48  *Methylobacterium*: 10/16  *Polaromonas*: 8/49 |
| SoxC | 3 | | 3.5 | | Sulfane dehydrogenase subunit SoxC;  Sulfite oxidase SoxC;  Sulfur dehydrogenase subunit SoxC. | *Cupriavidus alkaliphilus*: 1/1.5  *Bradyrhizobium yuanmingense*: 1/1  *Rhodoplanes* sp. Z2-YC6860: 1/1 | *Cupriavidus*: 1/1.5  *Bradyrhizobium*: 1/1  *Rhodoplanes*: 1/1 |
| SoxD | 1 | | 1 | | Sulfite dehydrogenase cytochrome subunit SoxD | *Variovorax* sp. WDL1: 1/1 | *Variovorax* : 1/1 |
| SoxX | 68 | | 201 | | Monoheme cytochrome SoxX;  Sulfur oxidation c-type cytochrome SoxX;  Sulfur oxidation protein SoxX;  Sulfur oxidation protein SoxX, partial. | *Thiobacillus denitrificans*: 23/109  *Limnobacter* sp. MED105: 5/5  *Limnobacter* sp. CACIAM 66H1: 3/43  *Thiomicrospira* sp. Kp2: 4/5  *Sulfuritalea hydrogenivorans*: 2/2 | *Thiobacillus*: 24/110  *Limnobacter*: 8/48  *Bradyrhizobium*: 8/8  *Thiomicrospira*: 5/6  *Sulfuritalea*: 2/2 |
| SoxY | 61 | | 104 | | Sulfur oxidation protein SoxY;  Thiosulfate oxidation carrier protein SoxY. | *Thiobacillus denitrificans*: 18/30  *Sulfuricaulis* *limicola*: 9/11  *Sulfurifustis* *variabilis*: 8/9  *Limnobacter* sp. MED105: 4/15  *Thiocapsa marina*: 2/2 | *Thiobacillus*: 18/40  *Bradyrhizobium*: 7/12  *Sulfuricaulis*: 9/11  *Sulfurifustis*: 8/9  *Limnobacter*: 4/15 |
| SoxZ | 57 | | 172 | | Sulfur oxidation protein SoxZ;  Thiosulfate oxidation carrier complex protein SoxZ. | *Thiobacillus dentirifcans:*20/35  *Limnobacter* sp. MED105: 2/23  *Sulfurifustic variabilis*: 7/27  *Sulfuricaulis* *limicola*: 5/45  *Variovorax* sp. WDL1: 2/3 | *Bradyrhizobium*: 8/12  *Thiobacillus*: 20/35  *Sulfurifustic*: 7/27  *Sulfuricaulis*: 5/45  *Mycobacterium*: 3/3 |
| Sulfate adenylyltransferase | 34 | | 210 | | Sulfate adenylyltransferase | *Thiobacillus dentirifcans:* 19/79  *Thiobacillus thioparus:* 4/33  *Sulfuricaulis* *limicola*: 7/94  *Sulfurifustic variabilis*: 4/4 | *Thiobacillus:* 23/112  *Sulfuricaulis*: 7/94  *Sulfurifustic:* 4/4 |
| Sulfide:quinone oxidoreductase | 9 | | 13 | | Sulfide:quinone oxidoreductase;  Sulfide:quinone oxidoreductase, mitochondrial precursor. | *Bradyrhizobium* sp. DOA9: 5/8  *Thiobacillus* *denitrificans*: 3/4  *Nitrospira* sp. OLB3: 1/1 | *Bradyrhizobium*: 5/8  *Thiobacillus*: 3/4  *Nitrospira*: 1/1 |
| Sulfite oxidase | 42 | | 91 | | Putative sulfite oxidase subunit YedY;  Putative sulfite oxidase subunit YedZ;  Putative sulfite oxidase;  Sulfite oxidase and related enzymes;  Sulfite oxidase SoxC;  Sulfite oxidase subunit YedY;  Sulfite oxidase subunit YedZ;  Sulfite oxidase;  Sulfite oxidase-like oxidoreductase. | *Rhodoplanes* sp. Z2-YC6860: 6/6  *Sulfuricaulis* *limicola*: 4/35  *Sulfurifustis variabilis*: 3/3  *Longilinea arvoryzae*: 2/2  *Paraburkholderia telluris*: 1/2 | *Rhodoplanes*: 6/6  *Sulfuricaulis*: 4/35  *Sulfurifustis*: 3/3  *Paraburkholderia*: 2/3  *Longilinea*: 2/2 |
| Sulfur relay protein | 57 | | 183 | | MULTISPECIES: sulfur relay protein DsrH;  Sulfur relay protein DsrC;  Sulfur relay protein DsrH;  Sulfur relay protein TusB/DsrH;  Sulfur relay protein TusB;  Sulfur relay protein TusE;  Sulfur relay protein, TusE/DsrC/DsvC family;  Sulfur relay, DsrE-like protein. | *Sulfuricaulis* *limicola*: 21/81  *Sulfurifustis* *variabilis*: 17/18  *Prolixibacter bellariivorans*: 1/1  *Candidatus Magnetoovum chiemensis*: 1/1 | *Sulfuricaulis*: 21/81  *Sulfurifustis*: 17/18  *Prolixibacter*: 1/1 |
| Sulfur transfer protein | 26 | | 93 | | MULTISPECIES: sulfur transfer protein;  Sulfur transfer protein SirA;  Sulfur transfer protein TusE;  Putative SirA-like sulfur transfer protein. | *Sulfurifustis* *variabilis*: 18/53  *Sulfuricaulis* *limicola*: 6/37  *Variibacter gotjawalensis*: 1/1 | *Sulfurifustis*: 18/53  *Sulfuricaulis*: 6/37  *Variibacter*: 1/1 |
