# Supplementary material for: The Oxidative Metabolism of Fossil Hydrocarbons and Sulfide Minerals by the Lithobiontic Microbial Community Inhabiting Deep Subterrestrial Kupferschiefer Black Shale
Source: Front Microbiol. 2018 May 15;9:972. doi: 10.3389/fmicb.2018.00972 (PMC5962744; doi:10.3389/fmicb.2018.00972)
Supplement: Supplementary file 5 [file Table_2.DOCX]

Table S2. Archaeal PEGs related to oxidative metabolism of hydrocarbons identified in the metagenome of LMC and the dominant species and genera from which they originated.

| **Protein name** | **Unique reads** | **All reads** | **Protein specific name** | **Species: unique reads/all reads** | **Genera: unique reads/all reads** |
| --- | --- | --- | --- | --- | --- |
| **OXIDATIVE METABOLISM OF HYDROCARBONS** | | | | | |
| Alcohol dehydrogenase | 31 | 33 | Alcohol dehydrogenase;  Iron-containing alcohol dehydrogenase;  NAD-dependent alcohol dehydrogenase;  Putative alcohol dehydrogenase protein;  Putative alcohol dehydrogenase zinc-binding domain protein;  Putative NAD-dependent alcohol dehydrogenase;  Zinc-dependent alcohol dehydrogenase;  Zn-dependent alcohol dehydrogenase. | *Candidatus Nitrososphaera gargensis*: 22/23  *Nitrososphaera viennensis*: 4/4  *Thaumarchaeota archaeon* MY2: 3/4  *Candidatus* *Nitrososphaera* *evergladensis*: 2/2 | *Nitrososphaera*: 28/29  *Thaumarchaeota archaeon*: 3/4 |
| Aldehyde dehydrogenase | 6 | 7.5 | Aldehyde dehydrogenase;  Aldehyde dehydrogenase family protein;  Aldehyde dehydrogenase middle subunit;  NAD-dependent aldehyde dehydrogenase. | *Candidatus Nitrosotenuis cloacae*: 2/2  *Candidatus* *Nitrososphaera* *evergladensis*: 1/1.5  *Aeropyrum camini*: 1/1 | *Candidatus Nitrosotenuis*: 2/2  *Nitrososphaera*: 1/1.5  *Aeropyrum*: 1/1 |
| Alkanal monooxygenase | 3 | 5 | Luciferase-like monooxygenase family protein | *Candidatus Nitrososphaera gargensis*: 3/5 | *Nitrososphaera*: 3/5 |
| Dioxygenase  (metabolism of aromaticcompounds) | 5 | 6 | Ferredoxin subunit of nitrite reductase and ring-hydroxylating dioxygenase;  Putative dioxygenase. | *Candidatus* *Nitrososphaera* *evergladensis*: 4/4  *Candidatus Nitrosotalea devanaterra*: 1/1  *Candidatus Methanoperedens nitroreducens*: 0/1 | *Nitrososphaera*: 4/4  *Nitrosotalea*: 1/1  *Methanoperedens*: 0/1 |
| Dioxygenase  (metabolism of aliphatic compounds) | 1 | 1 | 2-Nitropropane dioxygenase-like enzyme | *Candidatus Nitrososphaera evergladensis*: 1/1 | *Nitrososphaera*: 1/1 |
| Methane monooxygenase | 1 | 2 | Ammonia monooxygenase/methane monooxygenase, subunit C | *Candidatus Nitrososphaera gargensis*: 1/2 | *Nitrososphaera*: 1/2 |
