# Supplementary material for: The Oxidative Metabolism of Fossil Hydrocarbons and Sulfide Minerals by the Lithobiontic Microbial Community Inhabiting Deep Subterrestrial Kupferschiefer Black Shale
Source: Front Microbiol. 2018 May 15;9:972. doi: 10.3389/fmicb.2018.00972 (PMC5962744; doi:10.3389/fmicb.2018.00972)
Supplement: Supplementary file 6 [file Table_3.DOCX]

Table S3. Fungal PEGs related to oxidative metabolism of hydrocarbons identified in the metagenome of LMC and the dominant species and genera from which they originated.

| **Protein name** | **Unique reads** | **All reads** | **Protein specific name** | **Species: unique reads/all reads** | **Genera: unique reads/all reads** |
| --- | --- | --- | --- | --- | --- |
| **OXIDATIVE METABOLISM OF HYDROCARBONS** | | | | | |
| Alcohol dehydrogenase | 3 | 4 | Alcohol dehydrogenase;  NADP-dependent alcohol dehydrogenase, putative. | *Aspergillus ruber*: 1/1  *Gonapodya prolifera*: 1/1  *Candida maltosa*: 1/1 | *Aspergillus*: 1/2  *Gonapodya*: 1/1  *Candida*: 1/1 |
| Aldehyde dehydrogenase | 1 | 3 | NAD-dependent aldehyde dehydrogenase;  Putative aldehyde dehydrogenase ALDH;  Similar to aldehyde dehydrogenase. | *Leptosphaeria maculans*: 1/1  *Aspergillus calidoustus*: 0/1  *Fusarium oxysporum*: 0/1 | *Leptosphaeria*: 1/1  *Aspergillus*: 0/1  *Fusarium*: 0/1 |
| Aryl-alcohol dehydrogenase | 1 | 1.6 | Aryl-alcohol dehydrogenase AAD14;  Aryl-alcohol dehydrogenase Aad14, putative;  Aryl-alcohol dehydrogenase, putative;  Putative aryl-alcohol dehydrogenase Aad14. | *Cordyceps militaris*: 1/1  *Aspergillus nomius*: 0/0.5  *Aspergillus oryzae*: 0/0.1 | *Cordyceps*: 1/1  *Aspergillus*: 0/0.6 |
| Cytochrome P450 | 3 | 4 | Cytochrome P450 monooxygenase, partial;  Cytochrome P450 monooxygenase, putative;  n-Alkane-inducible cytochrome P450;  Putative cytochrome P450;  Similar to cytochrome P450. | *Aspergillus clavatus*: 1/1  *Aspergillus fischeri*: 1/1  *Aspergillus ruber*: 1/1  *Botrytis cinerea*: 0/1 | *Aspergillus*: 3/3  *Botrytis*: 0/1 |
