# Supplementary material for: The Oxidative Metabolism of Fossil Hydrocarbons and Sulfide Minerals by the Lithobiontic Microbial Community Inhabiting Deep Subterrestrial Kupferschiefer Black Shale
Source: Front Microbiol. 2018 May 15;9:972. doi: 10.3389/fmicb.2018.00972 (PMC5962744; doi:10.3389/fmicb.2018.00972)
Supplement: Supplementary file 7 [file Table_4.DOCX]

Table S4. Bacterial proteins related to oxidative metabolism of hydrocarbons and sulfide minerals detected in the metaproteome of LMC and their repeatability in three samples; score – ions score, matches – number of matched fragment ions, seq(sig) – number of significant distinct sequences; “–“ not detected.

| [**Accession**](http://mascot.mslab-ibb.pl/mascot/cgi/master_results_2.pl?file=..%2Fdata%2F20141125%2FF055976.dat;_ignoreionsscorebelow=0.05;_minpeplen=5;_prefertaxonomy=0;_proteinfamilyswitch=0;_sigthreshold=0.05;_sortunassigned=scoredown;pr.show=reportbuilder;qo.sort=acc;qo.sortdir=asc;report=0;reptype=family#tc:rf:reportbuilder) **no.** | [**Protein**](http://mascot.mslab-ibb.pl/mascot/cgi/master_results_2.pl?file=..%2Fdata%2F20141125%2FF055976.dat;_ignoreionsscorebelow=0.05;_minpeplen=5;_prefertaxonomy=0;_proteinfamilyswitch=0;_sigthreshold=0.05;_sortunassigned=scoredown;pr.show=reportbuilder;qo.sort=desc;qo.sortdir=asc;report=0;reptype=family#tc:rf:reportbuilder) **name (NCBI)** | **Sample 1** | | | **Sample 2** | | | **Sample 3** | | |
| --- | --- | --- | --- | --- | --- | --- | --- | --- | --- | --- |
|  |  | [Score](http://mascot.mslab-ibb.pl/mascot/cgi/master_results_2.pl?file=..%2Fdata%2F20141125%2FF055976.dat;_ignoreionsscorebelow=0.05;_minpeplen=5;_prefertaxonomy=0;_proteinfamilyswitch=0;_sigthreshold=0.05;_sortunassigned=scoredown;pr.show=reportbuilder;qo.sort=score;qo.sortdir=asc;report=0;reptype=family#tc:rf:reportbuilder) | [Matches](http://mascot.mslab-ibb.pl/mascot/cgi/master_results_2.pl?file=..%2Fdata%2F20141125%2FF055976.dat;_ignoreionsscorebelow=0.05;_minpeplen=5;_prefertaxonomy=0;_proteinfamilyswitch=0;_sigthreshold=0.05;_sortunassigned=scoredown;pr.show=reportbuilder;qo.sort=matches;qo.sortdir=asc;report=0;reptype=family#tc:rf:reportbuilder) | [Seq(sig)](http://mascot.mslab-ibb.pl/mascot/cgi/master_results_2.pl?file=..%2Fdata%2F20141125%2FF055976.dat;_ignoreionsscorebelow=0.05;_minpeplen=5;_prefertaxonomy=0;_proteinfamilyswitch=0;_sigthreshold=0.05;_sortunassigned=scoredown;pr.show=reportbuilder;qo.sort=sequences-sig;qo.sortdir=asc;report=0;reptype=family#tc:rf:reportbuilder) | [Score](http://mascot.mslab-ibb.pl/mascot/cgi/master_results_2.pl?file=..%2Fdata%2F20141125%2FF055976.dat;_ignoreionsscorebelow=0.05;_minpeplen=5;_prefertaxonomy=0;_proteinfamilyswitch=0;_sigthreshold=0.05;_sortunassigned=scoredown;pr.show=reportbuilder;qo.sort=score;qo.sortdir=asc;report=0;reptype=family#tc:rf:reportbuilder) | [Matches](http://mascot.mslab-ibb.pl/mascot/cgi/master_results_2.pl?file=..%2Fdata%2F20141125%2FF055976.dat;_ignoreionsscorebelow=0.05;_minpeplen=5;_prefertaxonomy=0;_proteinfamilyswitch=0;_sigthreshold=0.05;_sortunassigned=scoredown;pr.show=reportbuilder;qo.sort=matches;qo.sortdir=asc;report=0;reptype=family#tc:rf:reportbuilder) | [Seq(sig)](http://mascot.mslab-ibb.pl/mascot/cgi/master_results_2.pl?file=..%2Fdata%2F20141125%2FF055976.dat;_ignoreionsscorebelow=0.05;_minpeplen=5;_prefertaxonomy=0;_proteinfamilyswitch=0;_sigthreshold=0.05;_sortunassigned=scoredown;pr.show=reportbuilder;qo.sort=sequences-sig;qo.sortdir=asc;report=0;reptype=family#tc:rf:reportbuilder) | [Score](http://mascot.mslab-ibb.pl/mascot/cgi/master_results_2.pl?file=..%2Fdata%2F20141125%2FF055976.dat;_ignoreionsscorebelow=0.05;_minpeplen=5;_prefertaxonomy=0;_proteinfamilyswitch=0;_sigthreshold=0.05;_sortunassigned=scoredown;pr.show=reportbuilder;qo.sort=score;qo.sortdir=asc;report=0;reptype=family#tc:rf:reportbuilder) | [Matches](http://mascot.mslab-ibb.pl/mascot/cgi/master_results_2.pl?file=..%2Fdata%2F20141125%2FF055976.dat;_ignoreionsscorebelow=0.05;_minpeplen=5;_prefertaxonomy=0;_proteinfamilyswitch=0;_sigthreshold=0.05;_sortunassigned=scoredown;pr.show=reportbuilder;qo.sort=matches;qo.sortdir=asc;report=0;reptype=family#tc:rf:reportbuilder) | [Seq(sig)](http://mascot.mslab-ibb.pl/mascot/cgi/master_results_2.pl?file=..%2Fdata%2F20141125%2FF055976.dat;_ignoreionsscorebelow=0.05;_minpeplen=5;_prefertaxonomy=0;_proteinfamilyswitch=0;_sigthreshold=0.05;_sortunassigned=scoredown;pr.show=reportbuilder;qo.sort=sequences-sig;qo.sortdir=asc;report=0;reptype=family#tc:rf:reportbuilder) |
| **OXIDATIVE METABOLISM OF ORGANIC CARBON COMPOUNDS** | | | | | | | | | | |
| [gi\|544647509](http://mascot.mslab-ibb.pl/mascot/cgi/protein_view.pl?file=..%2Fdata%2F20141125%2FF055945.dat;_ignoreionsscorebelow=0.05;_minpeplen=5;_msresflags=3138;_msresflags2=10;_sigthreshold=0.05;ave_thresh=55;db_idx=1;hit=gi%7C544647509;px=1;report=0) | 2-Haloacid dehalogenase (*Bradyrhizobium* sp. DFCI-1) | 58 | 1 | 1 | 64 | 2 | 1 | 81 | 3 | 1 |
| gi\|490244293 | 2-Nitropropane dioxygenase (*Cardiobacterium hominis*) | 59 | 1 | 1 | 60 | 2 | 1 | 57 | 1 | 1 |
| [gi\|496990711](http://mascot.mslab-ibb.pl/mascot/cgi/protein_view.pl?file=..%2Fdata%2F20141125%2FF055945.dat;_ignoreionsscorebelow=0.05;_minpeplen=5;_msresflags=3138;_msresflags2=10;_sigthreshold=0.05;ave_thresh=55;db_idx=1;hit=gi%7C496990711;px=1;report=0) | 2-Nitropropane dioxygenase (*Oribacterium* sp. oral taxon 108) | – | – | – | – | – | – | 74 | 1 | 1 |
| [gi\|491126456](http://mascot.mslab-ibb.pl/mascot/cgi/protein_view.pl?file=..%2Fdata%2F20141125%2FF055963.dat;_ignoreionsscorebelow=0.05;_minpeplen=5;_msresflags=3138;_msresflags2=10;_sigthreshold=0.05;ave_thresh=54;db_idx=1;hit=gi%7C491126456;px=1;report=0) | Alcohol dehydrogenase (*Acinetobacter* *johnsonii*) | – | – | – | 148 | 3 | 3 | – | – | – |
| [gi\|499688088](http://mascot.mslab-ibb.pl/mascot/cgi/protein_view.pl?file=..%2Fdata%2F20141125%2FF055963.dat;_ignoreionsscorebelow=0.05;_minpeplen=5;_msresflags=3138;_msresflags2=10;_sigthreshold=0.05;ave_thresh=54;db_idx=1;hit=gi%7C499688088;px=1;report=0) | Alcohol dehydrogenase (*Desulfovibrio* *alaskensis*) | – | – | – | 67 | 3 | 1 | – | – | – |
| [gi\|517727322](http://mascot.mslab-ibb.pl/mascot/cgi/protein_view.pl?file=..%2Fdata%2F20141125%2FF055976.dat;_ignoreionsscorebelow=0.05;_minpeplen=5;_msresflags=3138;_msresflags2=10;_sigthreshold=0.05;ave_thresh=54;db_idx=1;hit=gi%7C517727322;px=1;report=0) | Alcohol dehydrogenase (*Rhizobium* sp. 2MFCol3.1) | 65 | 2 | 1 | – | – | – | – | – | – |
| [gi\|481050357](http://mascot.mslab-ibb.pl/mascot/cgi/protein_view.pl?file=..%2Fdata%2F20141125%2FF055976.dat;_ignoreionsscorebelow=0.05;_minpeplen=5;_msresflags=3138;_msresflags2=10;_sigthreshold=0.05;ave_thresh=54;db_idx=1;hit=gi%7C481050357;px=1;report=0) | Alcohol dehydrogenase (*Salmonella enterica*) | 58 | 4 | 1 | 57 | 3 | 1 | 54 | 1 | 1 |
| [gi\|663398328](http://mascot.mslab-ibb.pl/mascot/cgi/protein_view.pl?file=..%2Fdata%2F20141125%2FF055963.dat;_ignoreionsscorebelow=0.05;_minpeplen=5;_msresflags=3138;_msresflags2=10;_sigthreshold=0.05;ave_thresh=54;db_idx=1;hit=gi%7C663398328;px=1;report=0) | Alcohol dehydrogenase (*Streptomyces purpeofuscus*) | 82 | 11 | 1 | 78 | 12 | 1 | 81 | 10 | 1 |
| [gi\|663143141](http://mascot.mslab-ibb.pl/mascot/cgi/protein_view.pl?file=..%2Fdata%2F20141125%2FF055945.dat;_ignoreionsscorebelow=0.05;_minpeplen=5;_msresflags=3138;_msresflags2=10;_sigthreshold=0.05;ave_thresh=55;db_idx=1;hit=gi%7C663143141;px=1;report=0) | Alcohol dehydrogenase (*Streptomyces* sp. NRRL S-813) | – | – | – | – | – | – | 37 | 1 | 1 |
| [gi\|490789497](http://mascot.mslab-ibb.pl/mascot/cgi/protein_view.pl?file=..%2Fdata%2F20141125%2FF055963.dat;_ignoreionsscorebelow=0.05;_minpeplen=5;_msresflags=3138;_msresflags2=10;_sigthreshold=0.05;ave_thresh=54;db_idx=1;hit=gi%7C490789497;px=1;report=0) | Aldehyde dehydrogenase (*Acinetobacter bohemicus*) | – | – | – | 156 | 5 | 4 | – | – | – |
| [gi\|491126465](http://mascot.mslab-ibb.pl/mascot/cgi/protein_view.pl?file=..%2Fdata%2F20141125%2FF055963.dat;_ignoreionsscorebelow=0.05;_minpeplen=5;_msresflags=3138;_msresflags2=10;_sigthreshold=0.05;ave_thresh=54;db_idx=1;hit=gi%7C491126465;px=1;report=0) | Aldehyde dehydrogenase (*Acinetobacter johnsonii*) | 112 | 5 | 5 | 352 | 12 | 7 | 177 | 3 | 2 |
| [gi\|491118422](http://mascot.mslab-ibb.pl/mascot/cgi/protein_view.pl?file=..%2Fdata%2F20141125%2FF055976.dat;_ignoreionsscorebelow=0.05;_minpeplen=5;_msresflags=3138;_msresflags2=10;_sigthreshold=0.05;ave_thresh=54;db_idx=1;hit=gi%7C491118422;px=1;report=0) | Aldehyde dehydrogenase (*Acinetobacter towneri*) | 119 | 4 | 3 | 92 | 3 | 3 | 82 | 1 | 1 |
| [gi\|493682336](http://mascot.mslab-ibb.pl/mascot/cgi/protein_view.pl?file=..%2Fdata%2F20141125%2FF055976.dat;_ignoreionsscorebelow=0.05;_minpeplen=5;_msresflags=3138;_msresflags2=10;_sigthreshold=0.05;ave_thresh=54;db_idx=1;hit=gi%7C493682336;px=1;report=0) | Aldehyde dehydrogenase (*Microcoleus vaginatus*) | 59 | 1 | 1 | – | – | – | – | – | – |
| [gi\|500262322](http://mascot.mslab-ibb.pl/mascot/cgi/protein_view.pl?file=..%2Fdata%2F20141125%2FF055963.dat;_ignoreionsscorebelow=0.05;_minpeplen=5;_msresflags=3138;_msresflags2=10;_sigthreshold=0.05;ave_thresh=54;db_idx=1;hit=gi%7C500262322;px=1;report=0) | Aldehyde dehydrogenase (*Pseudomonas stutzeri*) | – | – | – | 89 | 2 | 2 | – | – | – |
| [gi\|489377479](http://mascot.mslab-ibb.pl/mascot/cgi/protein_view.pl?file=..%2Fdata%2F20141125%2FF055976.dat;_ignoreionsscorebelow=0.05;_minpeplen=5;_msresflags=3138;_msresflags2=10;_sigthreshold=0.05;ave_thresh=54;db_idx=1;hit=gi%7C489377479;px=1;report=0) | Aldehyde dehydrogenase, partial (*Pseudomonas stutzeri*) | 83 | 1 | 1 | – | – | – | 84 | 1 | 1 |
| [gi\|495154682](http://mascot.mslab-ibb.pl/mascot/cgi/protein_view.pl?file=..%2Fdata%2F20141125%2FF055945.dat;_ignoreionsscorebelow=0.05;_minpeplen=5;_msresflags=3138;_msresflags2=10;_sigthreshold=0.05;ave_thresh=55;db_idx=1;hit=gi%7C495154682;px=1;report=0) | Alkylhydroperoxidase (*Ochrobactrum* sp. CDB2) | – | – | – | – | – | – | 59 | 1 | 1 |
| [gi\|9965291](http://mascot.mslab-ibb.pl/mascot/cgi/protein_view.pl?file=..%2Fdata%2F20141125%2FF055945.dat;_ignoreionsscorebelow=0.05;_minpeplen=5;_msresflags=3138;_msresflags2=10;_sigthreshold=0.05;ave_thresh=55;db_idx=1;hit=gi%7C9965291;px=1;report=0) | Cyclohexanol dehydrogenase (*Acinetobacter* sp. SE19) | 163 | 2 | 1 | 110 | 1 | 1 | 249 | 5 | 3 |
| [gi\|490775052](http://mascot.mslab-ibb.pl/mascot/cgi/protein_view.pl?file=..%2Fdata%2F20141125%2FF055963.dat;_ignoreionsscorebelow=0.05;_minpeplen=5;_msresflags=3138;_msresflags2=10;_sigthreshold=0.05;ave_thresh=54;db_idx=1;hit=gi%7C490775052;px=1;report=0) | Dienelactone hydrolase (*Acinetobacter haemolyticus*) | – | – | – | 91 | 5 | 3 | – | – | – |
| [gi\|517722695](http://mascot.mslab-ibb.pl/mascot/cgi/protein_view.pl?file=..%2Fdata%2F20141125%2FF055945.dat;_ignoreionsscorebelow=0.05;_minpeplen=5;_msresflags=3138;_msresflags2=10;_sigthreshold=0.05;ave_thresh=55;db_idx=1;hit=gi%7C517722695;px=1;report=0) | FAD-binding monooxygenase (*Streptomyces* sp. CNT302) | – | – | – | – | – | – | 65 | 1 | 1 |
| [gi\|306921967](http://mascot.mslab-ibb.pl/mascot/cgi/protein_view.pl?file=..%2Fdata%2F20141125%2FF055963.dat;_ignoreionsscorebelow=0.05;_minpeplen=5;_msresflags=3138;_msresflags2=10;_sigthreshold=0.05;ave_thresh=54;db_idx=1;hit=gi%7C306921967;px=1;report=0) | Particulate methane monooxygenase B-subunit (*Methylovulum miyakonense* HT12) | – | – | – | 62 | 1 | 1 | – | – | – |
| [gi\|489392338](http://mascot.mslab-ibb.pl/mascot/cgi/protein_view.pl?file=..%2Fdata%2F20141125%2FF055945.dat;_ignoreionsscorebelow=0.05;_minpeplen=5;_msresflags=3138;_msresflags2=10;_sigthreshold=0.05;ave_thresh=55;db_idx=1;hit=gi%7C489392338;px=1;report=0) | Quinoprotein alcohol dehydrogenase (*Pseudomonas stutzeri*) | – | – | – | 169 | 7 | 6 | 88 | 4 | 3 |
| **OXIDATIVE METABOLISM OF INORGANIC SULFUR COMPOUNDS** | | | | | | | | | | |
| [gi\|499630342](http://mascot.mslab-ibb.pl/mascot/cgi/protein_view.pl?file=..%2Fdata%2F20141125%2FF055963.dat;_ignoreionsscorebelow=0.05;_minpeplen=5;_msresflags=3138;_msresflags2=10;_sigthreshold=0.05;ave_thresh=54;db_idx=1;hit=gi%7C499630342;px=1;report=0) | Cytochrome SoxA (*Thiobacillus denitrificans*) | – | – | – | 44 | 2 | 1 | – | – | – |
| [gi\|517333069](http://mascot.mslab-ibb.pl/mascot/cgi/protein_view.pl?file=..%2Fdata%2F20141125%2FF055976.dat;_ignoreionsscorebelow=0.05;_minpeplen=5;_msresflags=3138;_msresflags2=10;_sigthreshold=0.05;ave_thresh=54;db_idx=1;hit=gi%7C517333069;px=1;report=0) | Cytochrome SoxA (*Thiobacillus thioparus*) | 74 | 2 | 1 | 45 | 1 | 1 | 58 | 1 | 1 |
| [gi\|499632259](http://mascot.mslab-ibb.pl/mascot/cgi/protein_view.pl?file=..%2Fdata%2F20141125%2FF055963.dat;_ignoreionsscorebelow=0.05;_minpeplen=5;_msresflags=3138;_msresflags2=10;_sigthreshold=0.05;ave_thresh=54;db_idx=1;hit=gi%7C499632259;px=1;report=0) | DsrH protein (*Thiobacillus denitrificans*) | – | – | – | 71 | 2 | 1 | – | – | – |
