# Supplementary material for: The Oxidative Metabolism of Fossil Hydrocarbons and Sulfide Minerals by the Lithobiontic Microbial Community Inhabiting Deep Subterrestrial Kupferschiefer Black Shale
Source: Front Microbiol. 2018 May 15;9:972. doi: 10.3389/fmicb.2018.00972 (PMC5962744; doi:10.3389/fmicb.2018.00972)
Supplement: Supplementary file 8 [file Table_5.DOCX]

Table S5. Archaeal proteins related to oxidative metabolism of hydrocarbons detected in the metaproteome of LMC and their repeatability in three samples; score – ions score, matches – number of matched fragment ions, seq(sig) – number of significant distinct sequences; “–“ not detected.

| [**Accession**](http://mascot.mslab-ibb.pl/mascot/cgi/master_results_2.pl?file=..%2Fdata%2F20141125%2FF055976.dat;_ignoreionsscorebelow=0.05;_minpeplen=5;_prefertaxonomy=0;_proteinfamilyswitch=0;_sigthreshold=0.05;_sortunassigned=scoredown;pr.show=reportbuilder;qo.sort=acc;qo.sortdir=asc;report=0;reptype=family#tc:rf:reportbuilder) **no.** | [**Protein**](http://mascot.mslab-ibb.pl/mascot/cgi/master_results_2.pl?file=..%2Fdata%2F20141125%2FF055976.dat;_ignoreionsscorebelow=0.05;_minpeplen=5;_prefertaxonomy=0;_proteinfamilyswitch=0;_sigthreshold=0.05;_sortunassigned=scoredown;pr.show=reportbuilder;qo.sort=desc;qo.sortdir=asc;report=0;reptype=family#tc:rf:reportbuilder) **name (NCBI)** | **Sample 1** | | | **Sample 2** | | | **Sample 3** | | |
| --- | --- | --- | --- | --- | --- | --- | --- | --- | --- | --- |
|  |  | [Score](http://mascot.mslab-ibb.pl/mascot/cgi/master_results_2.pl?file=..%2Fdata%2F20141125%2FF055976.dat;_ignoreionsscorebelow=0.05;_minpeplen=5;_prefertaxonomy=0;_proteinfamilyswitch=0;_sigthreshold=0.05;_sortunassigned=scoredown;pr.show=reportbuilder;qo.sort=score;qo.sortdir=asc;report=0;reptype=family#tc:rf:reportbuilder) | [Matches](http://mascot.mslab-ibb.pl/mascot/cgi/master_results_2.pl?file=..%2Fdata%2F20141125%2FF055976.dat;_ignoreionsscorebelow=0.05;_minpeplen=5;_prefertaxonomy=0;_proteinfamilyswitch=0;_sigthreshold=0.05;_sortunassigned=scoredown;pr.show=reportbuilder;qo.sort=matches;qo.sortdir=asc;report=0;reptype=family#tc:rf:reportbuilder) | [Seq(sig)](http://mascot.mslab-ibb.pl/mascot/cgi/master_results_2.pl?file=..%2Fdata%2F20141125%2FF055976.dat;_ignoreionsscorebelow=0.05;_minpeplen=5;_prefertaxonomy=0;_proteinfamilyswitch=0;_sigthreshold=0.05;_sortunassigned=scoredown;pr.show=reportbuilder;qo.sort=sequences-sig;qo.sortdir=asc;report=0;reptype=family#tc:rf:reportbuilder) | [Score](http://mascot.mslab-ibb.pl/mascot/cgi/master_results_2.pl?file=..%2Fdata%2F20141125%2FF055976.dat;_ignoreionsscorebelow=0.05;_minpeplen=5;_prefertaxonomy=0;_proteinfamilyswitch=0;_sigthreshold=0.05;_sortunassigned=scoredown;pr.show=reportbuilder;qo.sort=score;qo.sortdir=asc;report=0;reptype=family#tc:rf:reportbuilder) | [Matches](http://mascot.mslab-ibb.pl/mascot/cgi/master_results_2.pl?file=..%2Fdata%2F20141125%2FF055976.dat;_ignoreionsscorebelow=0.05;_minpeplen=5;_prefertaxonomy=0;_proteinfamilyswitch=0;_sigthreshold=0.05;_sortunassigned=scoredown;pr.show=reportbuilder;qo.sort=matches;qo.sortdir=asc;report=0;reptype=family#tc:rf:reportbuilder) | [Seq(sig)](http://mascot.mslab-ibb.pl/mascot/cgi/master_results_2.pl?file=..%2Fdata%2F20141125%2FF055976.dat;_ignoreionsscorebelow=0.05;_minpeplen=5;_prefertaxonomy=0;_proteinfamilyswitch=0;_sigthreshold=0.05;_sortunassigned=scoredown;pr.show=reportbuilder;qo.sort=sequences-sig;qo.sortdir=asc;report=0;reptype=family#tc:rf:reportbuilder) | [Score](http://mascot.mslab-ibb.pl/mascot/cgi/master_results_2.pl?file=..%2Fdata%2F20141125%2FF055976.dat;_ignoreionsscorebelow=0.05;_minpeplen=5;_prefertaxonomy=0;_proteinfamilyswitch=0;_sigthreshold=0.05;_sortunassigned=scoredown;pr.show=reportbuilder;qo.sort=score;qo.sortdir=asc;report=0;reptype=family#tc:rf:reportbuilder) | [Matches](http://mascot.mslab-ibb.pl/mascot/cgi/master_results_2.pl?file=..%2Fdata%2F20141125%2FF055976.dat;_ignoreionsscorebelow=0.05;_minpeplen=5;_prefertaxonomy=0;_proteinfamilyswitch=0;_sigthreshold=0.05;_sortunassigned=scoredown;pr.show=reportbuilder;qo.sort=matches;qo.sortdir=asc;report=0;reptype=family#tc:rf:reportbuilder) | [Seq(sig)](http://mascot.mslab-ibb.pl/mascot/cgi/master_results_2.pl?file=..%2Fdata%2F20141125%2FF055976.dat;_ignoreionsscorebelow=0.05;_minpeplen=5;_prefertaxonomy=0;_proteinfamilyswitch=0;_sigthreshold=0.05;_sortunassigned=scoredown;pr.show=reportbuilder;qo.sort=sequences-sig;qo.sortdir=asc;report=0;reptype=family#tc:rf:reportbuilder) |
| **OXIDATIVE METABOLISM OF HYDROCARBONS** | | | | | | | | | | |
| [gi\|493722543](http://mascot.mslab-ibb.pl/mascot/cgi/protein_view.pl?file=..%2Fdata%2F20160315%2FF084975.dat;_msresflags=3138;_msresflags2=10;ave_thresh=37;db_idx=1;hit=gi%7C493722543;px=1) | Aldehyde dehydrogenase (*Halobiforma nitratireducens*) | 30 | 3 | 1 | – | – | – | – | – | – |
| [gi\|495661027](http://mascot.mslab-ibb.pl/mascot/cgi/protein_view.pl?file=..%2Fdata%2F20160315%2FF084977.dat;_msresflags=3138;_msresflags2=10;ave_thresh=38;db_idx=1;hit=gi%7C495661027;px=1) | Aldehyde dehydrogenase (*Halosarcina pallida*) | – | – | – | – | – | – | 45 | 1 | 1 |
| [gi\|497674975](http://mascot.mslab-ibb.pl/mascot/cgi/protein_view.pl?file=..%2Fdata%2F20160315%2FF084977.dat;_msresflags=3138;_msresflags2=10;ave_thresh=38;db_idx=1;hit=gi%7C497674975;px=1) | Aldehyde oxidase (*Sulfolobus solfataricus*) | – | – | – | – | – | – | 38 | 3 | 1 |
| [gi\|496168735](http://mascot.mslab-ibb.pl/mascot/cgi/protein_view.pl?file=..%2Fdata%2F20160315%2FF084975.dat;_msresflags=3138;_msresflags2=10;ave_thresh=37;db_idx=1;hit=gi%7C496168735;px=1) | Alkyl hydroperoxide reductase/ thiol specific antioxidant/ Mal allergen (*Haloterrigena salina*) | 37 | 2 | 1 | – | – | – | – | – | – |
| [gi\|495254055](http://mascot.mslab-ibb.pl/mascot/cgi/protein_view.pl?file=..%2Fdata%2F20160315%2FF084975.dat;_msresflags=3138;_msresflags2=10;ave_thresh=37;db_idx=1;hit=gi%7C495254055;px=1) | Carboxymuconolactone decarboxylase (*Haladaptatus paucihalophilus*) | 48 | 1 | 1 | – | – | – | 44 | 1 | 1 |
| [gi\|493940867](http://mascot.mslab-ibb.pl/mascot/cgi/protein_view.pl?file=..%2Fdata%2F20160315%2FF084975.dat;_msresflags=3138;_msresflags2=10;ave_thresh=37;db_idx=1;hit=gi%7C493940867;px=1) | Cytochrome P450 (*Halosimplex carlsbadense*) | 55 | 2 | 1 | 41 | 2 | 1 | 56 | 2 | 1 |
| [gi\|499345574](http://mascot.mslab-ibb.pl/mascot/cgi/protein_view.pl?file=..%2Fdata%2F20160315%2FF084975.dat;_msresflags=3138;_msresflags2=10;ave_thresh=37;db_idx=1;hit=gi%7C499345574;px=1) | Dioxygenase (*Methanosarcina mazei*) | 41 | 2 | 1 | – | – | – | – | – | – |
| [gi\|495715363](http://mascot.mslab-ibb.pl/mascot/cgi/protein_view.pl?file=..%2Fdata%2F20160315%2FF084977.dat;_msresflags=3138;_msresflags2=10;ave_thresh=38;db_idx=1;hit=gi%7C495715363;px=1) | FAD-dependent monooxygenase (*Halorubrum californiense*) | – | – | – | – | – | – | 30 | 1 | 1 |
| [gi\|490155430](http://mascot.mslab-ibb.pl/mascot/cgi/protein_view.pl?file=..%2Fdata%2F20160315%2FF084975.dat;_msresflags=3138;_msresflags2=10;ave_thresh=37;db_idx=1;hit=gi%7C490155430;px=1) | Haloacid dehalogenase (*Halococcus morrhuae*) | 47 | 1 | 1 | 50 | 2 | 1 | 54 | 2 | 1 |
| [gi\|502709889](http://mascot.mslab-ibb.pl/mascot/cgi/protein_view.pl?file=..%2Fdata%2F20160315%2FF084975.dat;_msresflags=3138;_msresflags2=10;ave_thresh=37;db_idx=1;hit=gi%7C502709889;px=1) | Isoquinoline 1-oxidoreductase (*Haloterrigena turkmenica*) | 27 | 1 | 1 | – | – | – | 27 | 1 | 1 |
| [gi\|496169855](http://mascot.mslab-ibb.pl/mascot/cgi/protein_view.pl?file=..%2Fdata%2F20160315%2FF084976.dat;_msresflags=3138;_msresflags2=10;ave_thresh=38;db_idx=1;hit=gi%7C496169855;px=1) | Lignostilbene-alpha,beta-dioxygenase (*Haloterrigena salina*) | – | – | – | 39 | 2 | 1 | 54 | 1 | 1 |
| [gi\|558583858](http://mascot.mslab-ibb.pl/mascot/cgi/protein_view.pl?file=..%2Fdata%2F20160315%2FF084977.dat;_msresflags=3138;_msresflags2=10;ave_thresh=38;db_idx=1;hit=gi%7C558583858;px=1) | Lignostilbene-alpha,beta-dioxygenase related enzyme (uncultured archaeon A07HR67) | – | – | – | – | – | – | 44 | 7 | 1 |
| [gi\|490157586](http://mascot.mslab-ibb.pl/mascot/cgi/protein_view.pl?file=..%2Fdata%2F20160315%2FF084976.dat;_msresflags=3138;_msresflags2=10;ave_thresh=38;db_idx=1;hit=gi%7C490157586;px=1) | Small terminal subunit of phenylpropionate dioxygenase (*Haloferax mediterranei*) | – | – | – | 47 | 2 | 1 | 47 | 4 | 1 |
