# Supplementary material for: The Oxidative Metabolism of Fossil Hydrocarbons and Sulfide Minerals by the Lithobiontic Microbial Community Inhabiting Deep Subterrestrial Kupferschiefer Black Shale
Source: Front Microbiol. 2018 May 15;9:972. doi: 10.3389/fmicb.2018.00972 (PMC5962744; doi:10.3389/fmicb.2018.00972)
Supplement: Supplementary file 9 [file Table_6.DOCX]

Table S6. Fungal proteins related to oxidative metabolism of hydrocarbons detected in the metaproteome of LMC and their repeatability in three samples; score – ions score, matches – number of matched fragment ions, seq(sig) – number of significant distinct sequences; “–“ not detected.

| [**Accession**](http://mascot.mslab-ibb.pl/mascot/cgi/master_results_2.pl?file=..%2Fdata%2F20160315%2FF084978.dat;pr.show=reportbuilder;qo.sort=acc;qo.sortdir=asc#tc:rf:reportbuilder) **no.** | [**Protein**](http://mascot.mslab-ibb.pl/mascot/cgi/master_results_2.pl?file=..%2Fdata%2F20141125%2FF055976.dat;_ignoreionsscorebelow=0.05;_minpeplen=5;_prefertaxonomy=0;_proteinfamilyswitch=0;_sigthreshold=0.05;_sortunassigned=scoredown;pr.show=reportbuilder;qo.sort=desc;qo.sortdir=asc;report=0;reptype=family#tc:rf:reportbuilder) **name (NCBI)** | **Sample 1** | | | **Sample 2** | | | **Sample 3** | | |
| --- | --- | --- | --- | --- | --- | --- | --- | --- | --- | --- |
|  |  | [Score](http://mascot.mslab-ibb.pl/mascot/cgi/master_results_2.pl?file=..%2Fdata%2F20160315%2FF084978.dat;pr.show=reportbuilder;qo.sort=score;qo.sortdir=asc#tc:rf:reportbuilder) | [Matches](http://mascot.mslab-ibb.pl/mascot/cgi/master_results_2.pl?file=..%2Fdata%2F20160315%2FF084978.dat;pr.show=reportbuilder;qo.sort=matches;qo.sortdir=asc#tc:rf:reportbuilder) | [Seq(sig)](http://mascot.mslab-ibb.pl/mascot/cgi/master_results_2.pl?file=..%2Fdata%2F20141125%2FF055976.dat;_ignoreionsscorebelow=0.05;_minpeplen=5;_prefertaxonomy=0;_proteinfamilyswitch=0;_sigthreshold=0.05;_sortunassigned=scoredown;pr.show=reportbuilder;qo.sort=sequences-sig;qo.sortdir=asc;report=0;reptype=family#tc:rf:reportbuilder) | [Score](http://mascot.mslab-ibb.pl/mascot/cgi/master_results_2.pl?file=..%2Fdata%2F20160315%2FF084978.dat;pr.show=reportbuilder;qo.sort=score;qo.sortdir=asc#tc:rf:reportbuilder) | [Matches](http://mascot.mslab-ibb.pl/mascot/cgi/master_results_2.pl?file=..%2Fdata%2F20160315%2FF084978.dat;pr.show=reportbuilder;qo.sort=matches;qo.sortdir=asc#tc:rf:reportbuilder) | [Seq(sig)](http://mascot.mslab-ibb.pl/mascot/cgi/master_results_2.pl?file=..%2Fdata%2F20141125%2FF055976.dat;_ignoreionsscorebelow=0.05;_minpeplen=5;_prefertaxonomy=0;_proteinfamilyswitch=0;_sigthreshold=0.05;_sortunassigned=scoredown;pr.show=reportbuilder;qo.sort=sequences-sig;qo.sortdir=asc;report=0;reptype=family#tc:rf:reportbuilder) | [Score](http://mascot.mslab-ibb.pl/mascot/cgi/master_results_2.pl?file=..%2Fdata%2F20160315%2FF084978.dat;pr.show=reportbuilder;qo.sort=score;qo.sortdir=asc#tc:rf:reportbuilder) | [Matches](http://mascot.mslab-ibb.pl/mascot/cgi/master_results_2.pl?file=..%2Fdata%2F20160315%2FF084978.dat;pr.show=reportbuilder;qo.sort=matches;qo.sortdir=asc#tc:rf:reportbuilder) | [Seq(sig)](http://mascot.mslab-ibb.pl/mascot/cgi/master_results_2.pl?file=..%2Fdata%2F20141125%2FF055976.dat;_ignoreionsscorebelow=0.05;_minpeplen=5;_prefertaxonomy=0;_proteinfamilyswitch=0;_sigthreshold=0.05;_sortunassigned=scoredown;pr.show=reportbuilder;qo.sort=sequences-sig;qo.sortdir=asc;report=0;reptype=family#tc:rf:reportbuilder) |
| **OXIDATIVE METABOLISM OF HYDROCARBONS** | | | | | | | | | | |
| [gi\|310789550](http://mascot.mslab-ibb.pl/mascot/cgi/protein_view.pl?file=..%2Fdata%2F20160315%2FF084978.dat;_msresflags=3138;_msresflags2=10;ave_thresh=46;db_idx=1;hit=gi%7C310789550;px=1) | Alcohol dehydrogenase GroES-like domain-containing protein (*Colletotrichum graminicola* M1.001) | 52 | 1 | 1 | – | – | – | – | – | – |
| [gi\|701779583](http://mascot.mslab-ibb.pl/mascot/cgi/protein_view.pl?file=..%2Fdata%2F20160315%2FF084978.dat;_msresflags=3138;_msresflags2=10;ave_thresh=46;db_idx=1;hit=gi%7C701779583;px=1) | Aldehyde dehydrogenase B (*Beauveria bassiana* D1-5) | 57 | 1 | 1 | – | – | – | – | – | – |
| [gi\|562971244](http://mascot.mslab-ibb.pl/mascot/cgi/protein_view.pl?file=..%2Fdata%2F20160315%2FF084978.dat;_msresflags=3138;_msresflags2=10;ave_thresh=46;db_idx=1;hit=gi%7C562971244;px=1) | Aryl-alcohol dehydrogenase (*Ogataea parapolymorpha* DL-1) | 52 | 1 | 1 | 46 | 2 | 1 | 44 | 3 | 1 |
| [gi\|296804017](http://mascot.mslab-ibb.pl/mascot/cgi/protein_view.pl?file=..%2Fdata%2F20160315%2FF084980.dat;_msresflags=3138;_msresflags2=10;ave_thresh=46;db_idx=1;hit=gi%7C296804017;px=1) | Cytochrome P450 (*Arthroderma otae* CBS 113480) | – | – | – | – | – | – | 52 | 1 | 1 |
| [gi\|662517686](http://mascot.mslab-ibb.pl/mascot/cgi/protein_view.pl?file=..%2Fdata%2F20160315%2FF084980.dat;_msresflags=3138;_msresflags2=10;ave_thresh=46;db_idx=1;hit=gi%7C662517686;px=1) | Cytochrome P450 (*Aureobasidium pullulans* var. namibiae CBS 147.97) | – | – | – | – | – | – | 46 | 1 | 1 |
| [gi\|628852908](http://mascot.mslab-ibb.pl/mascot/cgi/protein_view.pl?file=..%2Fdata%2F20160315%2FF084979.dat;_msresflags=3138;_msresflags2=10;ave_thresh=46;db_idx=1;hit=gi%7C628852908;px=1) | Cytochrome P450 (*Coniophora puteana* RWD-64-598 SS2) | – | – | – | 75 | 2 | 1 | – | – | – |
| [gi\|407924436](http://mascot.mslab-ibb.pl/mascot/cgi/protein_view.pl?file=..%2Fdata%2F20160315%2FF084979.dat;_msresflags=3138;_msresflags2=10;ave_thresh=46;db_idx=1;hit=gi%7C407924436;px=1) | Cytochrome P450 (*Macrophomina phaseolina* MS6) | – | – | – | 31 | 1 | 1 | – | – | – |
| [gi\|354961641](http://mascot.mslab-ibb.pl/mascot/cgi/protein_view.pl?file=..%2Fdata%2F20160315%2FF084980.dat;_msresflags=3138;_msresflags2=10;ave_thresh=46;db_idx=1;hit=gi%7C354961641;px=1) | Cytochrome P450 (*Phanerochaete chrysosporium*) | – | – | – | – | – | – | 50 | 1 | 1 |
| [gi\|599117208](http://mascot.mslab-ibb.pl/mascot/cgi/protein_view.pl?file=..%2Fdata%2F20160315%2FF084980.dat;_msresflags=3138;_msresflags2=10;ave_thresh=46;db_idx=1;hit=gi%7C599117208;px=1) | Cytochrome P450 (*Punctularia strigosozonata* HHB-11173 SS5) | – | – | – | – | – | – | 46 | 2 | 1 |
| [gi\|401883262](http://mascot.mslab-ibb.pl/mascot/cgi/protein_view.pl?file=..%2Fdata%2F20160315%2FF084978.dat;_msresflags=3138;_msresflags2=10;ave_thresh=46;db_idx=1;hit=gi%7C401883262;px=1) | Cytochrome P450 (*Trichosporon asahii* var. asahii CBS 2479) | 51 | 2 | 1 | – | – | – | 54 | 2 | 1 |
| [gi\|242772031](http://mascot.mslab-ibb.pl/mascot/cgi/protein_view.pl?file=..%2Fdata%2F20160315%2FF084979.dat;_msresflags=3138;_msresflags2=10;ave_thresh=46;db_idx=1;hit=gi%7C242772031;px=1) | Flavin-binding monooxygenase-like protein (*Talaromyces stipitatus* ATCC 10500) | – | – | – | 98 | 3 | 1 | 63 | 1 | 1 |
| [gi\|734657820](http://mascot.mslab-ibb.pl/mascot/cgi/protein_view.pl?file=..%2Fdata%2F20160315%2FF084978.dat;_msresflags=3138;_msresflags2=10;ave_thresh=46;db_idx=1;hit=gi%7C734657820;px=1) | Glucose-methanol-choline oxidoreductase (*Metarhizium album* ARSEF 1941) | 51 | 3 | 1 | – | – | – | – | – | – |
| [gi\|402218539](http://mascot.mslab-ibb.pl/mascot/cgi/protein_view.pl?file=..%2Fdata%2F20160315%2FF084980.dat;_msresflags=3138;_msresflags2=10;ave_thresh=46;db_idx=1;hit=gi%7C402218539;px=1) | NADPH-dependent alcohol dehydrogenase (*Dacryopinax* sp. DJM-731 SS1) | – | – | – | – | – | – | 48 | 2 | 1 |
| [gi\|599151466](http://mascot.mslab-ibb.pl/mascot/cgi/protein_view.pl?file=..%2Fdata%2F20160315%2FF084980.dat;_msresflags=3138;_msresflags2=10;ave_thresh=46;db_idx=1;hit=gi%7C599151466;px=1) | Putative cytochrome P450 (*Aspergillus ruber* CBS 135680) | – | – | – | – | – | – | 45 | 1 | 1 |
| [gi\|629669795](http://mascot.mslab-ibb.pl/mascot/cgi/protein_view.pl?file=..%2Fdata%2F20160315%2FF084978.dat;_msresflags=3138;_msresflags2=10;ave_thresh=46;db_idx=1;hit=gi%7C629669795;px=1) | Putative cytochrome p450 protein (*Eutypa lata* UCREL1) | 56 | 1 | 1 | – | – | – | – | – | – |
