# Supplementary material for: The Oxidative Metabolism of Fossil Hydrocarbons and Sulfide Minerals by the Lithobiontic Microbial Community Inhabiting Deep Subterrestrial Kupferschiefer Black Shale
Source: Front Microbiol. 2018 May 15;9:972. doi: 10.3389/fmicb.2018.00972 (PMC5962744; doi:10.3389/fmicb.2018.00972)
Supplement: Supplementary file 10 [file Table_7.DOCX]

Table S7. Geochemical characteristics of the studied weathered black shale and the unweathered black shale. The pyrolitic characteristics of kerogen is presented in Fig. 8. The detailed results of extractable organic matter composition are presented in Fig. 9 and in Supporting Information Fig. S2 and Table S8.

| **Geochemical characteristics** | **Weathered black shale** | **Unweathered black shale** |
| --- | --- | --- |
| **CHARACTERISTICS OF KEROGEN** | | |
| Free hydrocarbons (mg hydrocarbons / g rock) | 0.04 | 0.953 |
| Hydrocarbon potential (mg hydrocarbons / g rock) | 0.30 | 27.41 |
| Cracking temperature (°C) | 459 | 430 |
| Hydrogen index (mg hydrocarbons /g TOC) | 25 | 303 |
| Oxygen index (mg CO_2_ /g TOC) | 205 | 8.33 |
| Total organic carbon (wt%) | 1.19 | 9.04 |
| Residual carbon (wt%) | 1.09 | 6.65 |
| Pyrolyzed carbon (wt%) | 0.10 | 2.38 |
| **COMPOSITION OF EXTRACTABLE ORGANIC MATTER (sum of peak area (%))** | | |
| Aromatic hydrocarbons and derivatives | 23.46 | 13.60 |
| Aliphatic hydrocarbons and derivatives | 6.83 | 49.62 |
| Alcohols | 3.23 | 0.46 |
| Aldehydes | 0.13 | 0.38 |
| Ketones | 5.00 | 0.54 |
| Carboxylic acids | 26.24 | 4.57 |
| Esters | 7.69 | 0.92 |
| Other oxidized organic compounds | 3.83 | 1.24 |
| Other organic compounds | 13.05 | 6.92 |
| **SULFUR SPECIATION (wt%)** | | |
| S_total_ | 2.03 | 3.35 |
| S_organic_ | 1.75 | 2.40 |
| S_pyritic_ | 0.20 | 0.50 |
| S_sulfidic_ | <0.01 | 0.39 |
| S_sulfate_ | 0.08 | 0.06 |
