# Supplementary material for: The Oxidative Metabolism of Fossil Hydrocarbons and Sulfide Minerals by the Lithobiontic Microbial Community Inhabiting Deep Subterrestrial Kupferschiefer Black Shale
Source: Front Microbiol. 2018 May 15;9:972. doi: 10.3389/fmicb.2018.00972 (PMC5962744; doi:10.3389/fmicb.2018.00972)
Supplement: Supplementary file 11 [file Table_8.DOCX]

Table S8. Organic compounds detected in triplicate dichloromethane/methanol extracts of the studied black shale; Rt. – retention time, Prob. – probability, Area – peak area, nd – not detected, gray area – compounds identified with probability ≥ 80%. Total ion current chromatograms are presented in Fig. S2.

| **Rt. (min)** | **Sample 1** | | | **Sample 2** | | | **Sample 3** | | |
| --- | --- | --- | --- | --- | --- | --- | --- | --- | --- |
|  | **Compound name** | **Prob.**  **(%)** | **Area (%)** | **Compound name** | **Prob.**  **(%)** | **Area (%)** | **Compound name** | **Prob.**  **(%)** | **Area (%)** |
| 4.554 | nd | – | – | nd | – | – | 1-Oxyl-2,2,5,5-tetramethyl-3-(2-(4-azidobenzoyl)ethenyl)pyrroline | 25 | 0.48 |
| 4.639 | nd | – | – | nd | – | – | 2,4,6-Cycloheptatrien-1-one | 78 | 5.34 |
| 4.979 | nd | – | – | nd | – | – | Octamethylcyclotetrasiloxane | 58 | 0.13 |
| 5.786 | Trimethyl-[4-trimethylsilyloxy-2,3-bis(trimethylsilylsulfanyl)butoxy]silane | 36 | 0.37 | Silicate anion tetramer | 32 | 0.52 | Dodecamethylpentasiloxane | 95 | 11.6 |
| 6.313 | nd | – | – | nd | – | – | nd | – | – |
| 6.950 | 4-Oxopentanoic acid | 47 | 0.22 | nd | – | – | 2-[(Ethylsulfanyl)methyl]furan | 38 | 0.50 |
| 8.377 | nd | – | – | nd | – | – | Decamethyltetrasiloxane | 53 | 0.46 |
| 8.572 | Nonanoic acid | 83 | 1.19 | 3-Aminopropanoic acid | 50 | 0.62 | nd | – | – |
| 9.898 | nd | – | – | nd | – | – | 6-Methyl-2,4,7-tris[(trimethylsilyl)oxy]pteridine | 12 | 0.13 |
| 10.136 | Butan-2-ol | 50 | 0.65 | 3-Aminopropanoic acid | 38 | 0.32 | nd | – | – |
| 11.113 | nd | – | – | nd | – | – | Decamethyltetrasiloxane | 38 | 0.23 |
| 11.758 | nd | – | – | nd | – | – | Dodecamethylcyclohexasiloxane | 86 | 0.23 |
| 13.066 | Undec-10-ynoic acid | 59 | 0.71 | Dodecanoic acid | 38 | 0.83 | nd | – | – |
| 13.185 | nd | – | – | nd | – | – | Dodecanoic acid | 91 | 0.40 |
| 13.296 | nd | – | – | nd | – | – | Hexadecamethylheptasiloxane | 47 | 0.12 |
| 14.001 | 1,2-Dihydrophenanthrene | 59 | 0.10 | nd | – | – | nd | – | – |
| 14.341 | 1-(2-Hydroxyphenyl)propan-1-one | 53 | 0.17 | nd | – | – | nd | – | – |
| 14.434 | 2,3,4,5,6-Pentafluorobenzaldehyde | 38 | 0.19 | nd | – | – | 6-Methyl-N-(5-methyl-4,5-dihydro-1,3-thiazol-2-yl)-2-pyridinamine | 55 | 0.21 |
| 14.451 | nd | – | – | nd | – | – | 4-(2,4,4-Trimethylpentan-2-yl)phenol | 46 | 0.20 |
| 14.545 | nd | – | – | nd | – | – | nd | – | – |
| 14.604 | Tetradecan-1-ol | 78 | 0.19 | 1-Triethylsilyloxydodecane | 9 | 0.10 | nd | – | – |
| 14.621 | nd | – | – | nd | – | – | 4-(3-Hydroxy-2,6,6-trimethyl-1-cyclohexen-1-yl)-3-penten-2-one | 46 | 0.10 |
| 14.672 | Dibenzo[b,d]thiophene | 91 | 0.73 | Dibenzo[b,d]thiophene | 90 | 0.55 | nd | – | – |
| 14.715 | nd | – | – | nd | – | – | Dimethyl-prop-2-enyl-tetradecan-2-yloxysilane | 53 | 0.19 |
| 15.037 | Phenanthrene | 87 | 1.44 | Phenanthrene | 91 | 1.51 | nd | – | – |
| 15.063 | nd | – | – | nd | – | – | 4-(2,4,4-Trimethylpentan-2-yl)phenol | 78 | 0.16 |
| 15.539 | 2,3-Dimethylbenzaldehyde | 47 | 0.11 | nd | – | – | nd | – | – |
| 15.590 | 2-Phenyl-2,3-dihydroindene | 80 | 0.11 | nd | – | – | nd | – | – |
| 15.649 | 4,5-Dimethoxy-2-(2-propenyl)phenol | 78 | 0.29 | 4,5-Dimethoxy-2-(2-propenyl)phenol | 83 | 0.22 | nd | – | – |
| 15.725 | Tetradecanoic acid | 83 | 0.76 | Tetradecanoic acid | 72 | 1.30 | nd | – | – |
| 15.836 | 2,3-Dimethyl-9H-fluorene | 78 | 0.11 | nd | – | – | Tetradecanoic acid | 99 | 1.27 |
| 16.006 | 3-Methyldibenzo[b,d]thiophene | 90 | 2.10 | Benzene-1,2-dicarboxylic acid, bis(2-methylpropyl) ester | 72 | 6.13 | nd | – | – |
| 16.142 | nd | – | – | nd | – | – | Benzene-1,2-dicarboxylic acid, bis(2-methylpropyl) ester | 52 | 0.57 |
| 16.261 | 4-Methyldibenzo[b,d]thiophene | 91 | 0.84 | 4-Methyldibenzo[b,d]thiophene | 90 | 0.64 | nd | – | – |
| 16.337 | Nonadecane | 72 | 0.12 | 6-Ethyl-2-methyldecane | 83 | 0.90 | nd | – | – |
| 16.388 | 7-Benzhydrylidenebicyclo[2.2.1]hept-5-en-2-one | 64 | 0.10 | nd | – | – | nd | – | – |
| 16.439 | nd | – | – | nd | – | – | Tetradecane | 76 | 0.31 |
| 16.524 | 1a,9b-Dihydro-1H-cyclopropa[l]phenanthrene | 93 | 1.40 | 1a,9b-Dihydro-1H-cyclopropa[l]phenanthrene | 70 | 0.31 | nd | – | – |
| 16.533 | nd | – | – | nd | – | – | Hexadecanenitrile | 91 | 0.27 |
| 16.550 | nd | – | – | 3,4,6-Trimethylazulene-1-carbaldehyde | 83 | 0.17 | nd | – | – |
| 16.609 | 1-Methylanthracene | 93 | 1.64 | 2-Methylanthracene | 93 | 0.46 | nd | – | – |
| 16.668 | nd | – | – | nd | – | – | 2-Methylphenanthrene | 96 | 0.32 |
| 16.677 | Dodecanoic acid, methyl ester | 78 | 0.27 | Tridecanoic acid, methyl ester | 78 | 0.58 | nd | – | – |
| 16.753 | nd | – | – | nd | – | – | 1-Methylanthracene | 93 | 0.62 |
| 16.847 | 2-Methylphenanthrene | 93 | 2.25 | 2-Methylanthracene | 96 | 1.12 | nd | – | – |
| 16.915 | 2-Methylanthracene | 94 | 1.58 | 1-Methylanthracene | 96 | 0.54 | nd | – | – |
| 16.966 | 1H-Indole-2-carboxylic acid, 1-(trimethylsilyl)-5-[(trimethylsilyl)oxy]-,trimethylsilyl ester | 72 | 0.47 | Pentanoic acid | 59 | 0.59 | nd | – | – |
| 16.974 | nd | – | – | nd | – | – | 2-Methylanthracene | 96 | 0.57 |
| 17.059 | Phenylacetic acid | 9 | 0.22 | nd | – | – | Pentadecanoic acid | 99 | 1.01 |
| 17.119 | Hexadecan-1-ol | 64 | 0.30 | Hexadecan-1-ol | 72 | 0.32 | nd | – | – |
| 17.153 | nd | – | – | nd | – | – | Decan-2-ol | 49 | 0.24 |
| 17.212 | Benzene-1,3-dicarboxylic acid, dibutyl ester | 53 | 0.22 | Benzene-1,2-dicarboxylic acid, dibutyl ester | 91 | 4.59 | nd | – | – |
| 17.221 | nd | – | – | nd | – | – | Hexadecan-1-ol | 90 | 0.64 |
| 17.272 | 2,8-Dimethyldibenzo[b,d]thiophene | 93 | 0.29 | 2,8-Dimethyldibenzo[b,d]thiophene | 94 | 0.26 | nd | – | – |
| 17.314 | nd | – | – | nd |  |  | Benzene-1,2-dicarboxylic acid, dibutyl ester | 90 | 0.70 |
| 17.467 | 2-Phenylnaphthalene | 93 | 2.91 | 2-Methyl-2-phenylbutanedioic acid, ethyl ester | 91 | 2.74 | nd | – | – |
| 17.552 | (E)-But-2-enedioic acid, 2-methyl-4-methoxybutyl octyl ester | 42 | 0.44 | Tetradecane | 91 | 1.26 | nd | – | – |
| 17.594 | nd | – | – | nd | – | – | 2-Phenylnaphthalene | 95 | 0.97 |
| 17.654 | nd | – | – | nd | – | – | 3,7-Dimethylnonane | 52 | 0.65 |
| 17.781 | 3,7-Dimethyldibenzo[b,d]thiophene | 35 | 0.18 | 2,8-Dimethyldibenzo[b,d]thiophene | 53 | 0.11 | nd | – | – |
| 17.849 | (2E)-3-Phenylprop-2-en-1-ol | 50 | 0.89 | Propan-2-ol | 27 | 0.85 | nd | – | – |
| 17.900 | N'-[(E)-1-(4-Ethylphenyl)ethylidene]-2-pyridinecarbohydrazide | 49 | 0.32 | 2-(4-Hydroxyphenyl)-2-(4-cyanomethyleneoxyphenyl)propane | 43 | 0.28 | nd | – | – |
| 17.943 | 2-Benzylnaphthalene | 64 | 0.33 | 2-Benzylnaphthalene | 83 | 0.20 | nd | – | – |
| 17.960 | nd | – | – | nd | – | – | 4,6-Dimethyldibenzo[b,d]thiophene | 59 | 1.55 |
| 18.011 | 2,7-Dimethylphenanthrene | 46 | 0.44 | 3,9-Dimethylimidazo[4,5-f]quinolin-2-amine | 46 | 0.36 | nd | – | – |
| 18.070 | 1,1'-(1,2-Ethynediyl)bis(4-methylbenzene) | 91 | 0.15 | nd | – | – | nd | – | – |
| 18.138 | nd | – | – | nd | – | – | 2,7-Dimethylphenanthrene | 89 | 0.42 |
| 18.155 | Hexadecanoic acid | 91 | 4.64 | Tetradecanoic acid | 93 | 7.68 | nd | – | – |
| 18.232 | 2,5-Dimethylphenanthrene | 98 | 1.25 | 2,7-Dimethylanthracene | 87 | 0.88 | nd | – | – |
| 18.249 | nd | – | – | nd | – | – | Hexadecanoic acid | 99 | 14.0 |
| 18.317 | 2,5-Dimethylphenanthrene | 94 | 0.62 | 2,7-Dimethylanthracene | 72 | 0.42 | nd | – | – |
| 18.342 | nd | – | – | nd | – | – | 2,7-Dimethylanthracene | 96 | 1.06 |
| 18.393 | 2,7-Dimethylphenanthrene | 91 | 0.30 | 2,7-Dimethylphenanthrene | 80 | 0.22 | nd | – | – |
| 18.461 | 1,4-Dimethylanthracene | 87 | 0.35 | 1,4-Dimethylanthracene | 49 | 0.29 | nd | – | – |
| 18.512 | (2E,6E)-2,6-Bis(2-methylpropylidene)cyclohexan-1-one | 90 | 0.29 | 2-Methyl-3-phenyl-1H-indene | 59 | 0.20 | nd | – | – |
| 18.597 | Pyrene | 91 | 0.70 | Pyrene | 81 | 0.61 | nd | – | – |
| 18.682 | 2,7-Dimethylphenanthrene | 87 | 0.12 | nd | – | – | nd | – | – |
| 18.724 | nd | – | – | Heptadecane | 91 | 1.16 | Fluoranthene | 97 | 0.91 |
| 18.775 | (Z)-2,3-Dihydro-1H-cylonona[def]biphenylene | 78 | 0.51 | 2-Benzylnaphthalene | 86 | 0.30 | nd | – | – |
| 18.826 | nd | – | – | nd | – | – | Heneicosane | 91 | 0.49 |
| 18.877 | (Z)-2,3-Dihydro-1H-cylonona[def]biphenylene | 81 | 0.84 | 11H-Indolo[3,2-c]quinoline | 64 | 0.64 | nd | – | – |
| 18.903 | nd | – | – | nd | – | – | (Z)-2,3-Dihydro-1H-cylonona[def]biphenylene | 74 | 0.47 |
| 18.988 | 6-Methyl-8-phenylbenzocyclohepten-7-one | 86 | 0.45 | 2-Benzylnaphthalene | 80 | 0.32 | nd | – | – |
| 19.005 | nd | – | – | nd | – | – | 2-Benzylnaphthalene | 90 | 0.75 |
| 19.056 | Cis-4b,5,9b,10-Tetrahydro-5-methylindeno[1,2-b]indole | 16 | 0.12 | Heneicosanoic acid, methyl ester | 40 | 0.17 | nd | – | – |
| 19.132 | 2-Benzylnaphthalene | 59 | 0.33 | 2-Benzylnaphthalene | 59 | 0.39 | nd | – | – |
| 19.226 | Pyrene | 87 | 0.72 | Pyrene | 94 | 0.74 | nd | – | – |
| 19.243 | nd | – | – | nd | – | – | 6,7,8,9-Tetrahydro-1H-pyrrolo[3,4-c]quinoline-1,3,4(2H,5H)-trione | 64 | 0.56 |
| 19.277 | Indeno[2,1-b]chromene | 60 | 0.66 | 1H-Phenanthro[9,10-c]pyrazole | 64 | 0.46 | nd | – | – |
| 19.378 | nd |  |  | nd | – | – | Heptadecanoic acid | 89 | 1.36 |
| 19.404 | Octadecan-1-ol | 90 | 0.72 | Octadecan-1-ol | 87 | 1.75 | nd | – | – |
| 19.497 | Indeno[2,1-b]chromene | 62 | 1.34 | 1H-Phenanthro[9,10-c]pyrazole | 59 | 0.85 | nd | – | – |
| 19.506 | nd | – | – | nd | – | – | Octadecan-1-ol | 91 | 1.99 |
| 19.616 | nd | – | – | nd | – | – | Indeno[2,1-b]chromene | 90 | 0.96 |
| 19.625 | 5-Methyl-6,7,8,9-tetrahydroisothiazolo[5,4-c]isoquinolin-1(2H)-one | 64 | 0.22 | 4-Acetyl-4,5-dichlorocyclohexene-3,6-dione | 64 | 0.29 | nd | – | – |
| 19.727 | 2-Ethoxyethyl trimethylsilyl ether | 37 | 0.24 | N,N-Dimethylthioacetamide | 43 | 0.21 | nd | – | – |
| 19.812 | 6-Acetyl-2,2-dimethyl-7-chromanol | 35 | 0.12 | nd | – | – | nd | – | – |
| 19.854 | 12H-naphthacen-5-one | 58 | 0.27 | Pentadecane | 80 | 1.36 | nd | – | – |
| 19.948 | nd | – | – | (Z)-1,3,4,5-Tetrahydro-3,3,3',4'-tetramethyl-5-oxo-2,2'-pyrromethene | 58 | 0.18 | nd | – | – |
| 19.956 | nd | – | – | nd | – | – | Pentadecane | 91 | 0.81 |
| 19.982 | 2-Methylfluoranthene | 87 | 0.66 | 11H-Benzo[b]fluorene | 86 | 0.24 | nd | – | – |
| 20.084 | (Z)-Octadec-9-enoic acid | 97 | 0.85 | Trimethylsilyl (9Z)-9-octadecenoate | 52 | 0.86 | nd | – | – |
| 20.101 | nd | – | – | nd |  |  | 11H-Benzo[b]fluorene | 86 | 0.64 |
| 20.160 | (E)-Octadec-11-enoic acid | 74 | 0.21 | 9-Hexadecenal | 44 | 0.29 | nd | – | – |
| 20.186 | nd | – | – | nd | – | – | (Z)-Octadec-9-enoic acid | 95 | 0.90 |
| 20.372 | Octadecanoic acid | 94 | 7.35 | Octadecanoic acid | 98 | 7.57 | nd | – | – |
| 20.466 | nd | – | – | nd | – | – | Octadecanoic acid | 99 | 18.2 |
| 20.517 | 7H-Benzo[c]fluorene | 91 | 0.53 | 11H-Benzo[b]fluorene | 72 | 0.43 | nd | – | – |
| 20.602 | 3-Methylpyrene | 91 | 0.42 | 4-Methylpyrene | 60 | 0.24 | nd | – | – |
| 20.627 | nd | – | – | nd | – | – | 11H-Benzo[b]fluorene | 76 | 0.69 |
| 20.704 | 2-Methylphenanthro[9,10-b]furan | 90 | 0.75 | 2-Methylphenanthro[9,10-b]furan | 90 | 0.67 | 9H-Tribenzo[a,c,e)cycloheptene | 46 | 0.67 |
| 20.797 | nd | – | – | nd | – | – | 2-Methylphenanthro[9,10-b]furan | 90 | 0.96 |
| 20.857 | 3-Methylpyrene | 95 | 0.87 | 4-Methylpyrene | 64 | 0.64 | nd | – | – |
| 20.933 | 3-Methylpyrene | 97 | 0.77 | Pentadecane | 87 | 1.71 | nd | – | – |
| 20.959 | nd | – | – | nd | – | – | 1-Methylpyrene | 96 | 0.62 |
| 21.027 | nd | – | – | nd | – | – | Tricosane | 83 | 1.19 |
| 21.171 |  | – | – | 7,12-Dihydro-2-methylbenzo[a]anthracene | 53 | 0.35 | nd | – | – |
| 21.256 | 2-Acetyl-5,8-dimethoxytetralin | 80 | 0.17 | nd | – | – | nd | – | – |
| 21.281 | nd | – | – | nd | – | – | 4-Phenyldibenzofuran | 53 | 0.69 |
| 21.299 | nd | – | – | Butanoic acid, 2-methyl-, 1-methyl ethyl ester | 27 | 1.03 | nd | – | – |
| 21.383 | 9-Phenyl-9H-fluorene | 64 | 0.11 | nd | – | – | nd | – | – |
| 21.511 |  | – | – | Eicosan-1-ol | 25 | 0.18 | nd | – | – |
| 21.545 | 6-Chloro-1-phenyl-1,3-dihydro-2H-benzimidazol-2-one | 43 | 0.61 | 1-Phenyldibenzofuran | 64 | 0.30 | nd | – | – |
| 21.579 | (3aS,6aR,9aR,9bS)-3,6,9-Trimethylidene-3a,4,5,6a,7,8,9a,9b-octahydroazuleno[4,5-b]furan-2-one | 72 | 0.28 | nd | – | – | nd | – | – |
| 21.647 | 2-(4-Methoxyphenyl)-N,N,2-trimethyl-1-pyrrolamine | 64 | 0.64 | 2,2,5-trimethyl-2'(H)-5',6'-dihydropyrano[3',4'-g]indan-1-one | 58 | 0.28 | nd | – | – |
| 21.655 | nd | – | – | nd | – | – | 10-Methyl-11H-benzo[a]fluoren-11-one | 59 | 0.53 |
| 21.749 | 4-Phenyldibenzofuran | 59 | 0.11 | nd | – | – | nd | – | – |
| 21.766 | nd | – | – | nd | – | – | 6-Acetyl-1,4-dimethoxynaphthalene | 53 | 0.53 |
| 21.842 | 2,2,5-Trimethyl-2'(H)-5',6'-dihydropyrano[3',4'-g]indan-1-one | 74 | 0.54 | 3,4-Dimethoxy-5-methyl-2,2'-bipyridine | 86 | 0.26 | nd | – | – |
| 21.919 | 7H-Benzo[de]anthracen-7-one | 93 | 0.49 | nd | – | – | nd | – | – |
| 21.936 | nd | – | – | nd | – | – | 1,2-Diphenylbenzene | 64 | 0.61 |
| 21.953 | nd | – | – | Tetracosane | 93 | 1.78 | nd | – | – |
| 22.012 | (1R,4aS)-1,4a-Dimethyl-7-propan-2-yl-2,3,4,9,10,10a-hexahydrophenanthrene-1-carboxylic acid (Dehydroabietic acid) | 60 | 1.16 | nd | – | – | nd | – | – |
| 22.063 | nd | – | – | nd | – | – | Tetracosane | 96 | 0.78 |
| 22.114 | nd | – | – | nd | – | – | 4,5-Dihydro-3H-benzo[cd]pyren-5-ol | 38 | 0.78 |
| 22.131 | Benzo[b]naphtho[2,3-d]thiophene | 97 | 1.68 | Benzo[b]naphtho[1,2-d]thiophene | 95 | 1.46 | nd | – | – |
| 22.233 | nd | – | – | nd | – | – | Benzo[b]naphtho[2,3-d]thiophene | 97 | 1.22 |
| 22.403 | 2,3,4,4a,10,10a-Hexahydro-10a-hydroxy-1H-thioxanthen-1-one | 56 | 0.67 | 1,3,6,8-Tetramethylanthracene | 41 | 0.40 | nd | – | – |
| 22.471 | 1,4-Diphenylbenzene | 58 | 0.16 | 2-(1-Methyl-1H-indol-2-yl)quinolin | 25 | 0.10 | nd | – | – |
| 22.649 | nd | – | – | nd | – | – | nd | – | – |
| 22.649 | 1,2-Diphenylcyclopent-2-en-1-one | 47 | 0.19 | Benzo[b]naphtho[1,2-d]thiophene | 27 | 0.16 | nd | – | – |
| 22.853 | Benzo[b]anthracene | 81 | 0.48 | Benzo[b]anthracene | 95 | 0.51 | nd | – | – |
| 22.946 | Triphenylene | 96 | 2.43 | Benzo[a]anthracene | 96 | 2.82 | nd | – | – |
| 22.964 | nd | – | – | nd | – | – | Triphenylene | 92 | 0.27 |
| 23.048 | nd | – | – | nd | – | – | Triphenylene | 86 | 3.34 |
| 23.133 | 5H-Benzocycloheptene-1,2-dicarboxylic acid, 6,7,8,9-tetrahydro-3,4-dimethyl-, dimethyl ester | 53 | 0.15 | 5,12-Naphthacenequinone | 38 | 0.10 | nd | – | – |
| 23.201 | 9-Benzylidene-9H-fluorene | 78 | 0.52 | 4,5-Dihydrobenzo[def]chrysene | 45 | 0.50 | nd | – | – |
| 23.278 | nd | – | – | 3-Mercapto-2-methyl-4,5-dihydrofuran | 27 | 0.37 | nd | – | – |
| 23.303 | nd | – | – | nd | – | – | 9-Phenylanthracene | 95 | 0.35 |
| 23.354 | 9-Benzylidene-9H-fluorene | 50 | 0.40 | 3-Methylbenzo[b]naphtho[2,1-d]thiophene | 43 | 0.25 |  |  |  |
| 23.465 | nd | – | – | nd | – | – | 4a,8-Dimethyl-3-methylenedecahydroazuleno[6,5-b]furan-2,5-dione | 70 | 0.13 |
| 23.473 | Docosan-1-ol | 72 | 0.28 | nd | – | – | nd | – | – |
| 23.490 | nd | – | – | Benzene-1,2-dicarboxylic acid | 91 | 7.39 | nd | – | – |
| 23.533 | 2-Diphenylmethylcarbonylthiazole | 53 | 0.48 | nd | – | – | nd | – | – |
| 23.592 | nd | – | – | nd | – | – | Benzene-1,2-dicarboxylic acid, mono(2-ethylhexyl) ester | 91 | 5.67 |
| 23.618 | nd | – | – | 10b-Methoxy-6,10b-dihydroindolo[2,3-b][1,4]-benzodiazepin-12(11H)-one | 64 | 0.19 | nd | – | – |
| 23.652 | 7,7,11,11-Tetramethyl-7H,11H-benz[1,8]indolizino[2,3,4,5,6-defg]acridine | 64 | 0.34 | nd | – | – | nd | – | – |
| 23.720 | 2-Methylbenzo[b]naphtho[2,1-d]thiophene | 81 | 0.14 | 10-Methylbenzo[b]naphtho[1,2-d]thiophene | 83 | 0.23 | nd | – | – |
| 23.940 | 6-(6',7'-Dimethoxy-3',4'-dihydro-1'-isoquinolyl)-2-thioxo-5,6,7,8-tetrahydroquinazolin-4(3H)-one | 78 | 0.10 | 6-(6',7'-Dimethoxy-3',4'-dihydro-1'-isoquinolyl)-2-thioxo-5,6,7,8-tetrahydroquinazolin-4(3H)-one | 59 | 1.78 | nd | – | – |
| 23.991 | 2-Methylchrysene | 58 | 0.11 | nd | – | – | nd | – | – |
| 24.008 | nd | – | – | nd | – | – | Hexadecane | 95 | 0.85 |
| 24.093 | nd | – | – | 1-Methylchrysene | 81 | 0.54 | nd | – | – |
| 24.204 | nd | – | – | nd | – | – | 7-Methylbenzo[a]anthracene | 95 | 0.47 |
| 24.221 | 6-(Phenylthio)-9-ethylpurine | 46 | 0.75 | 1-Methylchrysene | 38 | 0.23 | nd | – | – |
| 24.314 | 10-Methyl-1,2-benzo[a]anthracene | 50 | 0.48 | nd | – | – | nd | – | – |
| 24.331 | nd | – | – | 5-Methylchrysene | 46 | 0.16 | (1r*,4as*,10s*,10ar*)-10-Hydroxy-7-methoxy-1-methyl-3,4,4a,9,10,10ahexahydrophenanthren-2(1H)-one | 53 | 0.48 |
| 24.382 | 2,2'-Binaphthalene | 58 | 0.34 | 2,2'-Binaphthalene | 68 | 0.18 | nd | – | – |
| 24.450 | nd | – | – | Methyl 13-amino-12-methoxypodocarpa-8,11,13-trien-19-oate | 46 | 0.31 | nd | – | – |
| 24.467 | 3-(4-Tert-butylphenyl)-3,6,6-trimethoxycyclohexa-1,4-dien | 95 | 0.82 | nd | – | – | nd | – | – |
| 24.569 | 4,5-Dibromo-3(2H)-pyridazinone | 50 | 0.44 | 1,1'-Binaphthalene | 47 | 0.17 | nd | – | – |
| 24.680 | 2-(4-Methoxyphenyl)-4-propyl-1,9b-dihydro-5-oxa-3,3a-diazacyclopenta[a]naphtalene | 93 | 0.20 | nd | – | – | nd | – | – |
| 24.747 | 2-Phenylphenanthrene | 60 | 0.48 | 2,2'-Binaphthalene | 58 | 0.24 | nd | – | – |
| 24.815 | 3-Ethyl-5-(2-ethylbutyl)octadecane | 59 | 0.26 | Heptacosane | 81 | 0.57 | nd | – | – |
| 24.858 | nd | – | – | nd | – | – | 2,2'-Binaphthalene | 50 | 0.29 |
| 24.892 | 9,9'-Spirobi(9H-fluorene) | 46 | 0.18 | nd | – | – | nd |  |  |
| 24.926 | nd | – | – | nd | – | – | 1-Iodotridecane | 90 | 0.77 |
| 24.934 | 13-Dimethylamino-8H-dibenzo[a,g]quinolizin-8-one | 53 | 0.28 | nd | – | – | nd | – | – |
| 25.045 | (2,3-Diphenylcyclopropen-1-yl)benzene | 70 | 0.64 | 1-(2-Naphthylmethyl)naphthalene | 43 | 0.14 | nd | – | – |
| 25.198 | nd | – | – | 2,3,5-Trichloro-6-methoxybenzoic acid | 30 | 0.25 | nd | – | – |
| 25.223 | 2-(2,4-Dimethylphenyl)sulfonyl-1,3,5-trimethylbenzene | 46 | 0.68 | nd | – | – | nd | – | – |
| 25.274 | 3-(4,5-Dimethoxy-2-vinylphenyl)-5,7,8-trimethoxy-2-methyl-isoquinolin-1(2H)-one | 86 | 0.36 | 15-Exo-acetoxy-3-acetyl-3,4,4a-endo,5,6,10b-hexahydro-6,10b-ethano-dibenzo(a,k)phenanthridine | 64 | 0.36 | nd | – | – |
| 25.317 | nd | – | – | nd | – | – | 3,9-Dimethylbenzo[a]anthracene | 25 | 0.34 |
| 25.342 | 3,8-Azo-4,7-methanocyclobuta[b]naphthalene, 2a,3,3a,4,7,7a,8,8a-octahydro-3,8-diphenyl-, (2a.alpha.,3.beta.,3a.alpha.,4.beta.,7.beta.,7a.alpha.,8.beta.,8a.alpha.)- | 43 | 0.35 | nd | – | – | nd | – | – |
| 25.368 | 3-Phenylpropionthionic acid, 2-[2-t-butyloxycarbonyl-amino-3-phenylpropionamido]-, propyl (ester) | 38 | 0.17 | (Z)-2,3-Dihydro-1H-cylonona[def]biphenylene | 43 | 0.38 | nd | – | – |
| 25.402 | 9,10-Dihydroxy-9,10-([1',6']-tricyclo[3.1.0.0(2,6)]hexano)anthracene | 43 | 0.41 | nd | – | – | nd | – | – |
| 25.521 | 1,4-Dimethylbenzo[c]phenanthrene | 58 | 0.48 | nd | – | – | nd | – | – |
| 25.614 | nd | – | – | Hexadecanamide | 53 | 0.56 | nd | – | – |
| 25.648 | (Z)-9-Octadecenamide | 72 | 7.13 | nd | – | – | nd | – | – |
| 25.691 | nd | – | – | 2,3-Diacetyl-6-methyl-4,5-diphenyl-o-diacylbenzole | 78 | 2.21 | nd | – | – |
| 25.801 | nd | – | – | Undecyl 1-thiohexopyranoside | 25 | 0.10 | nd | – | – |
| 25.818 | nd | – | – | nd | – | – | Octacosane | 94 | 0.95 |
| 25.920 | Perylene | 90 | 0.72 | Benzo[a]pyrene | 93 | 0.88 | nd | – | – |
| 25.971 | Isobutanol | 43 | 1.70 | nd | – | – | nd | – | – |
| 26.013 | nd | – | – | nd | – | – | Benzo[e]pyrene | 96 | 0.39 |
| 26.030 | nd | – | – | (6E,10E,14E,18E)-2,6,10,15,19,23-Hexamethyltetracosa-2,6,10,14,18,22-hexaene | 97 | 1.30 | nd | – | – |
| 26.047 | (6E,10E,14E,18E)-2,6,10,15,19,23-Hexamethyltetracosa-2,6,10,14,18,22-hexaene | 86 | 0.91 | nd | – | – | nd | – | – |
| 26.107 | Tetracosanoic acid | 25 | 0.68 | 1,2-Dibenzoyldibenzo[e,g]pyrrolo[1,2-a]pyridine | 53 | 0.10 | nd | – | – |
| 26.141 | nd | – | – | 1-(2-Dibenzothienyl)-2-(3-thienyl)ethene | 64 | 0.11 | nd | – | – |
| 26.158 | nd | – | – | nd | – | – | (6E,10E,14E,18E)-2,6,10,15,19,23-Hexamethyltetracosa-2,6,10,14,18,22-hexaene | 98 | 0.27 |
| 26.209 | 2,2'-Dibromo-1,1'-binaphthalene | 59 | 0.57 | Benzo[b]fluoranthene | 72 | 0.22 | nd | – | – |
| 26.387 | Dinaphtho[1,2-b:1',2'-d]furan | 81 | 0.86 | 4,5-Dihydrobenzo[a]pyrene-4,5-epoxide | 50 | 0.29 | nd | – | – |
| 26.515 | nd | – | – | Spiro[9H-fluorene-9,9'-tricyclo[3.3.1.02,8]nona[3,6]diene] | 59 | 0.24 | nd | – | – |
| 26.532 | Dinaphtho[1,2-b:1',2'-d]furan | 81 | 0.50 | nd | – | – | nd | – | – |
| 26.676 | nd | – | – | Benzo[a]pyrene | 95 | 1.32 | nd | – | – |
| 26.693 | Perylene | 81 | 1.34 | nd | – | – | nd | – | – |
| 26.812 | nd | – | – | nd | – | – | Docosane | 94 | 0.90 |
| 26.863 | Benzo[k]fluoranthene | 76 | 0.68 | Perylene | 74 | 0.33 | nd | – | – |
| 26.931 | Dibenzo[a,f]dibenzo[2,3:4,5]pentaleno[1,6-cd]pentalene,4b,8b,12b,16b-tetrahydro-, stereoisomer (9CI) | 53 | 0.32 | 2-(3-Bromo-benzyl)-2H-phthalazin-1-one | 35 | 0.20 | nd | – | – |
| 27.092 | Dinaphtho[1,2-b:1',2'-d]furan | 46 | 1.28 | 7,9-Diamino-5,6-dihydronaphtho[2',1',4,5]thieno[2,3-d]pyrimidine | 72 | 0.34 | nd | – | – |
| 27.203 | N-[(8E)-2-Methoxy-4a,6a-dimethyl-9-methyleneoctadecahydro-8H-naphtho[2',1':4,5]indeno[1,2-b]furan-8-ylidene]cyclohexanamine | 78 | 1.25 | nd | – | – | nd | – | – |
| 27.245 | nd | – | – | 4,5-Dihydro-3H-dinaphtho[2,1-c:1,2 -E]azepine | 64 | 0.77 | nd | – | – |
| 27.390 | 2-(4-Methoxyphenyl)-4-propyl-1,9b-dihydro-5-oxa-3,3a-diazacyclopenta[a]naphtalene | 53 | 0.58 | (2,3,4-Trichloro-2,4-cyclopentadienylidene)-(O-ethoxyphenyl)methane | 10 | 0.25 | nd | – | – |
| 27.508 | 2,6-Diphenylimidazo[1,2-b][1,2,4]triazin-3(4H)-one | 59 | 0.20 | nd | – | – | nd | – | – |
| 27.610 | 3-Hydroxy-6-methyl-2-(4-methylphenyl)-4H-chromen-4-one | 53 | 0.37 | 4-(N-(4-Ethylphenyl)amino)-5,6-dimethyl-7H-pyrrolo[2.3-d]pyrimidine | 78 | 0.24 | nd | – | – |
| 27.763 | 8H-Indeno[2,1-b]phenanthrene | 98 | 1.55 | 11H-Indeno[2,1-a]phenanthrene | 64 | 0.76 | nd | – | – |
| 27.814 | nd | – | – | Icosane | 50 | 0.37 | nd | – | – |
| 27.967 | nd | – | – | nd | – | – | Icosane | 96 | 0.33 |
| 27.984 | 1,2,3,3a,3b,4,5,6,7,7a,9,10,11,12-Tetradecahydrobenzo[b]fluoranthene | 72 | 0.34 | 1-Phenylbenzo(1,2-b-4,3-b)dithiophene | 59 | 0.27 | nd | – | – |
| 28.069 | 3,11,17-Tris[(trimethylsilyl)oxy]androst-5-ene | 62 | 0.31 | nd | – | – | nd | – | – |
| 28.078 | nd | – | – | 6-Methyl-5,5a,6,11,11a,12-hexahydroquinoxalino[2,3-b]quinoxaline | 27 | 0.20 | nd | – | – |
| 28.171 | 5-Bromo-2-methoxy-N-[2-(2-methyl-1H-indol-3-yl)ethyl]benzenesulfonamide | 27 | 0.13 | nd | – | – | nd | – | – |
| 28.265 | nd | – | – | 5,6,7,8,9,10-Hexahydrobenzo[ghi]perylene | 38 | 0.16 | nd | – | – |
| 28.273 | Carbamothioic acid, dimethyl-, s-[1-(tetrahydro-5-oxo-3-furanyl)-2-octenyl] ester, [r*,s*-(E)]- | 22 | 0.26 | nd | – | – | nd | – | – |
| 28.451 | Methylenedi-1,2-naphthalenediyl (2E,2'E)bis(3-phenylacrylate) | 17 | 0.13 | nd | – | – | nd | – | – |
| 28.545 | nd | – | – | 10-Methylbenzo[a]pyrene | 32 | 0.33 | nd | – | – |
| 28.562 | 11-Hydroxy-7,8,9,10-tetrahydrobenzo[b]xanthene-12-one | 38 | 0.39 | nd | – | – | nd | – | – |
| 28.664 | 6-Methoxy[1]benzothieno[2,3-c]naphthyridin-6(5H)-one | 45 | 0.24 | 3-Methylperylene | 58 | 0.19 | nd | – | – |
| 28.766 | 1-[2-(4-chlorophenoxy)ethyl]-2-(2,2-dimethylpropyl)-1H-1,3-benzimidazole | 37 | 0.13 | nd | – | – | nd | – | – |
| 28.893 | 1-(1,3-Dithian-2-yl)octan-3-yloxy-triethylsilane | 9 | 0.12 | nd | – | – | nd | – | – |
| 29.148 | 2-(4-Nitrophenyl)-3-methoxycarbonylmethyl-1,3-benzodiazine-8-one | 37 | 0.13 | nd | – | – | nd | – | – |
| 29.327 | nd | – | – | 1-(3'-Aminobenzyl)-7-methoxyisoquinolin-8-ol | 64 | 0.10 | nd | – | – |
| 29.343 | nd | – | – | nd | – | – | Trichloro(dodecyl)silane | 93 | 0.32 |
| 29.496 | nd | – | – | 2,6-Dimethoxy-10-methyl-1-anthracenecarbaldehyde | 53 | 0.16 | nd | – | – |
| 29.513 | 1-Phenyl-2-propyn-1-ol | 40 | 0.10 | nd | – | – | nd | – | – |
| 29.547 | 2-Chloro-n-[1-({[2-(2-methyl-1H-indol-3-yl)ethyl]amino}carbonyl)-2-phenylethenyl]benzamide | 53 | 0.16 | 5,6,7,8-Tetrahydro-3-quinolinecarb onitrile | 50 | 0.16 | nd | – | – |
| 29.955 | nd | – | – | 4-Formyl-2-(phenylmethyl)-5-phenylthiophene | 59 | 0.10 | nd | – | – |
| 30.337 | Cholesterol | 95 | 0.42 | Cholesterol | 89 | 0.30 | nd | – | – |
