# Supplementary material for: The Oxidative Metabolism of Fossil Hydrocarbons and Sulfide Minerals by the Lithobiontic Microbial Community Inhabiting Deep Subterrestrial Kupferschiefer Black Shale
Source: Front Microbiol. 2018 May 15;9:972. doi: 10.3389/fmicb.2018.00972 (PMC5962744; doi:10.3389/fmicb.2018.00972)
Supplement: Supplementary file 3 [file Presentation_1.ZIP › R19_filter-trim_BSc60_sup207_krona.html]

Javascript must be enabled to view this page.

members
magnitude
magnitudeUnassigned
count
unassigned
taxon
rank

020\_filtered-trimmed-deduplicated\_prinseq\_1\_BSc60\_sup001-207\_readN-taxId

2640564
node0.members.0.js
566048

2
2054146
superkingdom
39822
node1.members.0.js

203691
2558
88
phylum
node2.members.0.js

203692
2470
class
61
node3.members.0.js

319
family
node4.members.0.js
170
1648

1329
node5.members.0.js
171
genus

node6.members.0.js
order
19
761
136

137
742
65
family
node7.members.0.js

genus
157
266
node8.members.0.js

genus
146
node9.members.0.js
411

27329
200795
node10.members.0.js
511
phylum

7
class
node11.members.0.js
388447
595

240
order
768667

768668
family
240

240
genus
768669

240
node15.members.0.js
species
1382306

388448
order
348

388449
family
348

348
genus
363276

348
node19.members.0.js
363277
species

189775
1438
class
229
node20.members.0.js

subclass
85000
929

85001
order
929

929
255728
suborder

node24.members.0.js
family
6
929
85002

526
2056
genus

node26.members.0.js
526
2057
species

1206748
genus
397

397
node28.members.0.js
species
1206749

280
order
189776

189777
family
280

499
genus
280

species
500
node32.members.0.js
280

species
1617415
node33.members.0.js
3685

species
166587
node34.members.0.js
221

node35.members.0.js
class
225
3392
292625

375
node36.members.0.js
1703386
species

order
292629
2539

node38.members.0.js
family
400
2539
292628

349
475965
genus

node40.members.0.js
349
species
229921

genus
475961
320

species
360412
node42.members.0.js
320

475966
genus
210

210
node44.members.0.js
species
229920

334
genus
475960

node46.members.0.js
334
species
360411

244
1077886
genus

244
node48.members.0.js
species
869279

15
genus
node49.members.0.js
233189
682

167964
species
308
node50.members.0.js

359
node51.members.0.js
229919
species

253
node52.members.0.js
species
1703387

species
1617416
node53.members.0.js
607

class
475962
1596

order
475963
1596

1596
family
475964

1596
233191
genus

1596
node58.members.0.js
species
133453

1371
301297
node59.members.0.js
class
760

221
node60.members.0.js
genus
670486

1703396
species
390
node61.members.0.js

763
class
1382928

1382929
order
763

family
1382930
763

362
genus
node65.members.0.js
1382931
763

node66.members.0.js
401
872965
species

461
node67.members.0.js
species
1617414

504
genus
186475

186479
species
504
node69.members.0.js

node70.members.0.js
10557
species
1640513

node71.members.0.js
class
2
1628
32061

279
order
189772

189773
family
279

64
genus
node74.members.0.js
279

32064
1347
8
order
node75.members.0.js

node76.members.0.js
suborder
178
779
1508594

601
family
1106

node78.members.0.js
601
1107
genus

560
1508595
suborder

560
family
1508635

node81.members.0.js
65
genus
560
120961

node82.members.0.js
248
357808
species

species
120962
node83.members.0.js
247

species
1499966
node84.members.0.js
312

1802339
phylum
1716

93171
1716
161
genus
node86.members.0.js

1429439
species
node87.members.0.js
760

node88.members.0.js
795
1429438
species

node89.members.0.js
2
phylum
3566
1297

class
14
node90.members.0.js
188787
3564

node91.members.0.js
120
order
1064
118964

944
family
183710

944
node93.members.0.js
1298
genus

2486
68933
order

node95.members.0.js
family
265
2486
188786

65551
1604
genus
903
node96.members.0.js

node97.members.0.js
361
52023
species

node98.members.0.js
340
species
52022

node99.members.0.js
617
genus
270

565
phylum
node100.members.0.js
1117
12391

order
1890424
2117

998
family
1890438

genus
47251
998
node103.members.0.js

1890426
family
829

genus
1129
node105.members.0.js
829

family
1890436
290

1152
genus
290
node107.members.0.js

subclass
122
node108.members.0.js
1301283
4497

1574
1118
node109.members.0.js
order
1179

395
family
1890449

1125
395
genus
50
node111.members.0.js

species
1126
345
node112.members.0.js

order
2034
node113.members.0.js
1150
2801

418
1892249
family

43988
genus
418
node115.members.0.js

family
1892254
349

node117.members.0.js
349
genus
1158

class
307596
319

307595
order
319

319
1890422
family

33071
genus
node121.members.0.js
319

1161
4421
64
order
node122.members.0.js

70
family
node123.members.0.js
1185
336

1186
genus
266
node124.members.0.js

family
1892263
275

genus
1190
275
node126.members.0.js

1182
family
640

node128.members.0.js
272
genus
640
1203

node129.members.0.js
368
species
1245922

119859
1735
30
family
node130.members.0.js

1314
genus
482629

482630
species
node132.members.0.js
1314

node133.members.0.js
391
genus
111782

node134.members.0.js
490
family
1189

321
family
node135.members.0.js
1162
881

genus
1163
245
node136.members.0.js

genus
1177
node137.members.0.js
315

52604
order
472
node138.members.0.js

674
node139.members.0.js
1619079
species

species
1619075
node140.members.0.js
521

302481
201174
node141.members.0.js
phylum
392

94
class
node142.members.0.js
84992
1227

1133
84993
node143.members.0.js
order
3

307
1798913
family

genus
41949
307

node146.members.0.js
307
41950
species

823
84994
node147.members.0.js
254
family

node148.members.0.js
346
682522
genus

genus
53634
node149.members.0.js
223

84995
class
759

84996
order
462

462
family
84997

node153.members.0.js
462
42255
genus

297
order
1154584

family
1154585
297

297
genus
1154586

node157.members.0.js
297
1799656
species

node158.members.0.js
class
9911
298463
1760

order
292
node159.members.0.js
85013
1493

family
74712
1201

1201
node161.members.0.js
genus
1854

order
85010
5358

node163.members.0.js
family
1388
5358
2070

1133
genus
node164.members.0.js
1813
1384

330084
species
node165.members.0.js
251

node166.members.0.js
903
genus
1677
1847

node167.members.0.js
226
54010
species

269
node168.members.0.js
2074
species

node169.members.0.js
279
species
240495

417
node170.members.0.js
genus
1851

genus
2071
251
node171.members.0.js

genus
1835
241
node172.members.0.js

802
order
85004

31953
802
37
family
node174.members.0.js

genus
1678
765
node175.members.0.js

order
1643682
1265

node177.members.0.js
family
445
1265
85030

genus
114
node178.members.0.js
38501
429

138336
species
node179.members.0.js
315

88138
391
genus
174
node180.members.0.js

477641
species
217
node181.members.0.js

17875
85007
node182.members.0.js
order
608

2650
1653
node183.members.0.js
family
14

node184.members.0.js
genus
1598
2041
1716

1727
species
226
node185.members.0.js

node186.members.0.js
217
1718
species

95617
species
node187.members.0.js
595

node188.members.0.js
50
family
4742
85025

genus
2461
node189.members.0.js
1827
3220

260936
species
node190.members.0.js
426

1828
species
node191.members.0.js
333

1472
node192.members.0.js
1817
genus

node193.members.0.js
family
35
7468
1762

node194.members.0.js
4795
genus
7433
1763

node195.members.0.js
228
species
1768

species group
234
node196.members.0.js
120793
483

249
node197.members.0.js
species
1764

1068
77643
node198.members.0.js
species group
173

species
1773
node199.members.0.js
895

node200.members.0.js
63
species group
623
670516

species subgroup
670506
560

node202.members.0.js
560
36809
species

species
1789
node203.members.0.js
236

275
85029
family

37914
genus
275
node205.members.0.js

family
85026
2132

node207.members.0.js
genus
1370
2132
2053

329
node208.members.0.js
species
175628

433
node209.members.0.js
species
1241906

node210.members.0.js
493
1504317
species

species
312284
1787
node211.members.0.js

1643684
order
464

85031
family
464

87
genus
node214.members.0.js
53460
464

species
53461
377
node215.members.0.js

2048
85012
node216.members.0.js
order
10

291
family
node217.members.0.js
2004
1038

2000
genus
211
node218.members.0.js

node219.members.0.js
312
83681
genus

genus
2005
224
node220.members.0.js

node221.members.0.js
91
family
365
2012

node222.members.0.js
274
genus
1988

635
83676
node223.members.0.js
family
128

genus
2013
507
node224.members.0.js

374
85014
order

374
node226.members.0.js
family
85034

85006
235348
order
3538
node227.members.0.js

node228.members.0.js
379
family
2923
85016

1395
genus
node229.members.0.js
1707
1671

276
node230.members.0.js
species
1304865

458839
873
genus
121
node231.members.0.js

species
1386098
752
node232.members.0.js

node233.members.0.js
200
family
895
85020

463
genus
node234.members.0.js
43668
695

species
661979
232
node235.members.0.js

17564
1268
node236.members.0.js
family
1878

7522
genus
node237.members.0.js
1663
11539

1736342
species
node238.members.0.js
207

269454
species
676
node239.members.0.js

species
494419
438
node240.members.0.js

species
1736401
210
node241.members.0.js

species
1835723
node242.members.0.js
563

node243.members.0.js
696
species
479106

species
271431
node244.members.0.js
208

210
node245.members.0.js
290399
species

node246.members.0.js
541
1267973
species

268
node247.members.0.js
species
1736395

620
1269
node248.members.0.js
genus
98

species
1270
node249.members.0.js
522

1510
57493
node250.members.0.js
genus
1081

136273
species
429
node251.members.0.js

402
node252.members.0.js
genus
57494

631
596707
node253.members.0.js
144
genus

species
37927
node254.members.0.js
487

1742993
984
genus
412
node255.members.0.js

572
node256.members.0.js
species
361575

3380
85021
node257.members.0.js
family
509

296
node258.members.0.js
136099
genus

542
53355
node259.members.0.js
256
genus

286
node260.members.0.js
species
1619947

node261.members.0.js
genus
432
858
99479

node262.members.0.js
212
99480
species

214
node263.members.0.js
species
330834

genus
265
node264.members.0.js
53357
715

450
node265.members.0.js
1386088
species

node266.members.0.js
240
265976
genus

220
node267.members.0.js
genus
367298

node268.members.0.js
family
350
876
85017

node269.members.0.js
247
43676
genus

node270.members.0.js
279
157920
genus

337
family
145360

60919
genus
337
node272.members.0.js

145357
family
459
node273.members.0.js

950
85019
family

699
genus
node275.members.0.js
1696
950

1033736
species
node276.members.0.js
251

85023
202296
family
12541
node277.members.0.js

96492
1093
genus
809
node278.members.0.js

species
1736282
node279.members.0.js
284

1550
node280.members.0.js
genus
33886

2034
1711
genus
1257
node281.members.0.js

species
1736311
node282.members.0.js
226

species
95614
node283.members.0.js
228

genus
684
node284.members.0.js
110934
989

110935
species
node285.members.0.js
305

277
genus
881616

species
1500280
277
node287.members.0.js

node288.members.0.js
genus
8660
33464
33882

1736514
species
346
node289.members.0.js

2062
node290.members.0.js
400772
species

node291.members.0.js
458
species
1736323

475
node292.members.0.js
species
69370

node293.members.0.js
304
273677
species

species
1449058
node294.members.0.js
509

1692239
species
node295.members.0.js
212

1298
node296.members.0.js
1451261
species

node297.members.0.js
1885
species
84292

node298.members.0.js
1304
1714373
species

886
node299.members.0.js
species
712383

species
159896
315
node300.members.0.js

node301.members.0.js
222
species
1736478

1720
node302.members.0.js
species
1587523

976
node303.members.0.js
1177594
species

species
1778770
418
node304.members.0.js

species
1775956
293
node305.members.0.js

species
451764
node306.members.0.js
395

1348253
species
444
node307.members.0.js

236
node308.members.0.js
species
273678

species
1263625
280
node309.members.0.js

1736570
species
node310.members.0.js
221

269
node311.members.0.js
species
904291

node312.members.0.js
2210
36807
species

582680
species
node313.members.0.js
539

643
node314.members.0.js
species
1479651

species
82380
node315.members.0.js
334

species
671622
386
node316.members.0.js

162426
species
node317.members.0.js
691

species
450380
node318.members.0.js
1788

1770208
species
node319.members.0.js
229

2049
node320.members.0.js
species
1736553

species
2033
407
node321.members.0.js

439
genus
1195526

444608
species
439
node323.members.0.js

node324.members.0.js
116
genus
662
190323

1827323
species
312
node325.members.0.js

234
node326.members.0.js
species
1736333

node327.members.0.js
genus
175
3195
69578

node328.members.0.js
855
species
670052

710
node329.members.0.js
species
1529318

1001240
species
node330.members.0.js
1455

226
genus
518733

node332.members.0.js
226
species
412690

484
genus
256818

256821
species
node334.members.0.js
484

species
1655574
301
node335.members.0.js

322
427753
genus

node337.members.0.js
322
species
427754

node338.members.0.js
247
genus
960
1573

species
28447
node339.members.0.js
713

469
genus
235888

1112215
species
node341.members.0.js
469

13761
337004
genus

species
279828
node343.members.0.js
13761

genus
501022
1673

node345.members.0.js
1673
501023
species

447237
754
29
genus
node346.members.0.js

species
1736329
209
node347.members.0.js

species
1795630
node348.members.0.js
516

5392
110932
node349.members.0.js
genus
1080

1575
species
node350.members.0.js
1028

node351.members.0.js
662
1736426
species

1736337
species
node352.members.0.js
347

species
1736525
270
node353.members.0.js

856
node354.members.0.js
191389
species

species
1736496
node355.members.0.js
321

828
node356.members.0.js
144185
species

1705353
genus
313

species
708131
313
node358.members.0.js

286
529883
genus

529884
species
node360.members.0.js
286

genus
255204
node361.members.0.js
236

33877
7187
934
genus
node362.members.0.js

643
node363.members.0.js
293890
species

304
node364.members.0.js
species
1736467

1896
node365.members.0.js
species
1736390

node366.members.0.js
310
species
1735688

node367.members.0.js
544
species
453304

510
node368.members.0.js
species
1736601

630
node369.members.0.js
species
279572

species
1775951
node370.members.0.js
1416

249
genus
node371.members.0.js
46352
1293

node372.members.0.js
831
species
341722

213
node373.members.0.js
1346502
species

2249
55968
node374.members.0.js
genus
1074

491918
species
node375.members.0.js
264

node376.members.0.js
382
species
1292023

node377.members.0.js
292
664638
species

237
node378.members.0.js
species
1461260

node379.members.0.js
76351
genus
110469
355929

15952
node380.members.0.js
1736417
species

node381.members.0.js
18166
1736516
species

node382.members.0.js
family
201
434
85018

genus
211469
233

233
node384.members.0.js
111564
species

1042322
1696
family
8
node385.members.0.js

577469
979
459
genus
node386.members.0.js

520
node387.members.0.js
species
431489

709
node388.members.0.js
1217415
genus

85011
order
8625

8625
2062
node390.members.0.js
107
family

node391.members.0.js
220
2063
genus

8014
1883
node392.members.0.js
genus
7723

291
node393.members.0.js
species
591158

284
node394.members.0.js
228398
genus

809
order
1217098

809
family
1217100

281472
809
35
genus
node397.members.0.js

419479
species
node398.members.0.js
316

species
281473
node399.members.0.js
229

229
node400.members.0.js
702383
species

order
85008
2487

28056
2487
family
759
node402.members.0.js

764
node403.members.0.js
1873
genus

1865
genus
node404.members.0.js
594

node405.members.0.js
370
genus
168694

85009
6937
order
16
node406.members.0.js

node407.members.0.js
family
338
4100
85015

genus
348581
472

348582
species
472
node409.members.0.js

node410.members.0.js
1552
genus
2209
1839

species
196162
node411.members.0.js
439

species
1736409
218
node412.members.0.js

399
node413.members.0.js
2040
genus

node414.members.0.js
82
genus
356
182639

node415.members.0.js
274
460086
species

326
86795
node416.members.0.js
genus
117

209
node417.members.0.js
397278
species

node418.members.0.js
414
family
2821
31957

genus
493
node419.members.0.js
1743
1886

741
node420.members.0.js
species
1744

652
node421.members.0.js
species
1747

node422.members.0.js
172
genus
521
72763

species
1427523
node423.members.0.js
349

order
2037
1749

2049
1749
272
family
node425.members.0.js

node426.members.0.js
965
genus
1477
1654

280
node427.members.0.js
1325934
species

1660
species
node428.members.0.js
232

279
order
622452

83778
family
279
node430.members.0.js

359
node431.members.0.js
1650658
species

class
1497346
1266

588673
1266
order
269
node433.members.0.js

347
family
320583

genus
2
node435.members.0.js
191494
347

species
191495
node436.members.0.js
345

220
361606
family

genus
361607
node438.members.0.js
220

430
family
320599

207599
430
214
genus
node440.members.0.js

species
1298857
node441.members.0.js
216

node442.members.0.js
118
class
374
84998

node443.members.0.js
256
84999
order

281
262406
genus

281
node445.members.0.js
166501
species

372
508458
phylum

372
649775
class

649776
372
87
order
node448.members.0.js

649777
family
node449.members.0.js
285

1522316
species
274
node450.members.0.js

67812
phylum
node451.members.0.js
442

39068
976
node452.members.0.js
1415
phylum

class
63
node453.members.0.js
117743
3989

3926
200644
node454.members.0.js
order
376

2316
family
node455.members.0.js
49546
3550

genus
59732
node456.members.0.js
337

genus
237
897
node457.members.0.js

117747
1885
class
1
node458.members.0.js

1884
200666
node459.members.0.js
60
order

84566
1824
family
377
node460.members.0.js

238
node461.members.0.js
genus
423349

genus
28453
node462.members.0.js
243

genus
84567
node463.members.0.js
966

733
order
1100069

733
563843
node465.members.0.js
family
148

1779382
species
node466.members.0.js
286

1
genus
node467.members.0.js
29548
299

29549
species
node468.members.0.js
298

6755
768503
node469.members.0.js
class
2

node470.members.0.js
375
order
6753
768507

2692
89373
node471.members.0.js
1333
family

417
node472.members.0.js
genus
105

genus
107
546
node473.members.0.js

396
node474.members.0.js
genus
120831

node475.members.0.js
691
family
1186
563798

246875
genus
node476.members.0.js
495

200667
1387
family
705
node477.members.0.js

440
genus
396811

species
881893
440
node479.members.0.js

242
node480.members.0.js
genus
290180

296
family
node481.members.0.js
1853232
1113

genus
323449
344
node482.members.0.js

89966
genus
473
node483.members.0.js

1853228
class
18127

order
16
node485.members.0.js
1853229
18127

588
89374
node486.members.0.js
291
family

297
2349
genus

node488.members.0.js
297
2350
species

563835
17523
1085
family
node489.members.0.js

genus
398041
391

391
node491.members.0.js
species
1492898

2577
genus
1612198

species
1076522
node493.members.0.js
1300

species
925409
1277
node494.members.0.js

node495.members.0.js
genus
5651
7861
504481

979
node496.members.0.js
426421
species

node497.members.0.js
1231
1267211
species

genus
460073
767

node499.members.0.js
767
398037
species

296051
genus
315

species
249
node501.members.0.js
315

649460
genus
817

species
477680
node503.members.0.js
817

genus
88
node504.members.0.js
1004301
1958

node505.members.0.js
344
1812911
species

355
node506.members.0.js
1349421
species

species
1463156
node507.members.0.js
889

species
549295
node508.members.0.js
282

species
1691979
560
node509.members.0.js

379899
genus
378
node510.members.0.js

495
genus
354354

species
354356
node512.members.0.js
495

79328
genus
node513.members.0.js
319

3826
node514.members.0.js
1619897
species

2338
class
200643

node516.members.0.js
915
order
2338
171549

605
815
node517.members.0.js
family
3

816
genus
602
node518.members.0.js

family
171551
node519.members.0.js
466

171552
352
34
family
node520.members.0.js

genus
838
318
node521.members.0.js

555
genus
1763546

species
1763547
node523.members.0.js
555

1855361
phylum
node524.members.0.js
267

25570
203682
node525.members.0.js
385
phylum

class
412
node526.members.0.js
666505
700

node527.members.0.js
288
1703411
species

203683
24485
10
class
node528.members.0.js

order
322
node529.members.0.js
112
21740

4644
family
1914233

genus
1153
node531.members.0.js
113
2974

node532.members.0.js
1099
114
species

362
node533.members.0.js
species
1210884

species
1630693
360
node534.members.0.js

1670
genus
600332

1670
node536.members.0.js
360055
species

node537.members.0.js
family
513
14292
126

species
1799653
node538.members.0.js
473

2539
656899
genus

360056
species
node540.members.0.js
2539

118
2329
genus
10
node541.members.0.js

node542.members.0.js
256
species
1799657

node543.members.0.js
968
1636152
species

1632864
species
1095
node544.members.0.js

123
1788
11
genus
node545.members.0.js

species
125
node546.members.0.js
1190

node547.members.0.js
587
1632865
species

genus
265487
1088

species
124
node549.members.0.js
1088

1593
265488
node550.members.0.js
924
genus

species
1265734
431
node551.members.0.js

node552.members.0.js
238
1263869
species

node553.members.0.js
1590
1799658
species

genus
1649490
495

node555.members.0.js
495
119
species

1085
1649453
genus

species
122
node557.members.0.js
1085

1649480
genus
799

species
120
node559.members.0.js
799

1763524
2482
10
family
node560.members.0.js

node561.members.0.js
genus
2
574
127

node562.members.0.js
572
128
species

genus
466152
1898

1898
node564.members.0.js
species
466153

order
1127829
2735

2735
1127830
node566.members.0.js
family
101

1442
380240
node567.members.0.js
53
genus

795830
species
961
node568.members.0.js

species
380242
428
node569.members.0.js

genus
236756
243

node571.members.0.js
243
237368
species

571
360731
genus

node573.members.0.js
571
247490
species

378
380738
genus

378
node575.members.0.js
species
174633

1853220
phylum
229

class
1853221
229

229
order
1853223

family
1813606
229
node579.members.0.js

657
1752708
phylum

657
node581.members.0.js
1640509
species

node582.members.0.js
498
1379697
phylum

564
node583.members.0.js
species
1619078

node584.members.0.js
742
1619086
species

1649
1752716
phylum

1649
node586.members.0.js
species
1640508

species
1499967
node587.members.0.js
365

species
77133
8722
node588.members.0.js

373
phylum
node589.members.0.js
142182
20486

20113
class
219685

node591.members.0.js
15
order
20113
219686

node592.members.0.js
family
309
20098
219687

genus
1706036
8137

species
861299
node594.members.0.js
8137

11652
173479
node595.members.0.js
660
genus

1703356
species
679
node596.members.0.js

1379270
species
node597.members.0.js
3723

node598.members.0.js
630
species
1703359

node599.members.0.js
845
species
1703355

173480
species
3717
node600.members.0.js

1703357
species
node601.members.0.js
1133

species
1703358
node602.members.0.js
265

220
node603.members.0.js
phylum
1752720

672
phylum
1134404

10
class
node605.members.0.js
795747
672

795748
662
196
order
node606.members.0.js

family
1334117
466

genus
1134403
466

species
1134405
466
node609.members.0.js

node610.members.0.js
phylum
574
3470
74201

1657
203494
class

1657
48461
node612.members.0.js
226
order

node613.members.0.js
218
family
706
203557

node614.members.0.js
488
genus
2735

725
134621
family

725
genus
1032526

node617.members.0.js
725
1032527
species

57
class
node618.members.0.js
414999
694

415000
order
637

134623
637
family
409
node620.members.0.js

228
genus
178440

species
107709
node622.members.0.js
228

134549
545
118
class
node623.members.0.js

427
1836787
order

family
1836792
427

427
genus
295577

species
191863
node627.members.0.js
427

333
phylum
node628.members.0.js
1090
1394

species
1617413
node629.members.0.js
275

class
191410
786

order
191411
786

786
191412
node632.members.0.js
family
364

1091
genus
node633.members.0.js
422

node634.members.0.js
phylum
622
7680
57723

272
533205
class

order
574975
272

family
574976
272
node637.members.0.js

class
332159
1415

node639.members.0.js
order
2
1415
332160

988
family
332161

988
332162
node641.members.0.js
genus
2

332163
species
node642.members.0.js
986

425
genus
911113

360054
species
node644.members.0.js
425

1660251
species
node645.members.0.js
299

1562566
789
3
class
node646.members.0.js

309
genus
458032

458033
species
node648.members.0.js
309

genus
1562565
477

node650.members.0.js
477
454194
species

1813736
species
1752
node651.members.0.js

204432
class
2531

2531
204433
node653.members.0.js
29
order

family
1327
node654.members.0.js
204434
2502

658061
genus
311

311
node656.members.0.js
species
658062

species
1267535
node657.members.0.js
416

genus
33973
237
node658.members.0.js

genus
388463
211

211
node660.members.0.js
species
570835

512
1293497
phylum

512
1293498
class

1293499
order
512

node664.members.0.js
1
family
512
407032

35800
511
272
genus
node665.members.0.js

species
35801
239
node666.members.0.js

221216
phylum
node667.members.0.js
389

552
200783
phylum

class
98
node669.members.0.js
187857
552

454
node670.members.0.js
32069
order

node671.members.0.js
269
phylum
1752722

node672.members.0.js
807
species
1619076

phylum
67810
221
node673.members.0.js

234
node674.members.0.js
phylum
1752729

357
200918
phylum

188708
class
node676.members.0.js
357

635
node677.members.0.js
species
1799370

phylum
200940
354

67799
class
354

354
188710
order

188711
family
node681.members.0.js
354

species
1734400
node682.members.0.js
292

node683.members.0.js
422
1128421
species

640293
1221
5
phylum
node684.members.0.js

547
node685.members.0.js
species
1640516

669
genus
1170227

669
node687.members.0.js
671143
species

1034
67819
node688.members.0.js
phylum
708

species
1640515
node689.members.0.js
326

327
1752732
node690.members.0.js
110
phylum

node691.members.0.js
217
species
1618997

node692.members.0.js
18707
phylum
1456129
1224

node693.members.0.js
5891
class
374330
28216

3412
327159
node694.members.0.js
533
genus

species
1453999
node695.members.0.js
221

species
1454002
node696.members.0.js
252

1454001
species
376
node697.members.0.js

331
node698.members.0.js
species
1454003

species
1454004
275
node699.members.0.js

466
node700.members.0.js
species
327160

230
node701.members.0.js
1454000
species

species
1860102
node702.members.0.js
465

1457154
species
node703.members.0.js
263

order
1442155
1061

1442156
family
1061

1061
416212
node706.members.0.js
698
genus

species
1789004
node707.members.0.js
363

species
1703391
2642
node708.members.0.js

206350
4553
order
151
node709.members.0.js

family
400
node710.members.0.js
32011
4402

221
node711.members.0.js
1679002
genus

177
genus
node712.members.0.js
404
1304

405
species
node713.members.0.js
247

node714.members.0.js
880
1848039
species

359407
1286
genus
515
node715.members.0.js

359408
species
333
node716.members.0.js

node717.members.0.js
438
1055487
species

genus
81682
584
node718.members.0.js

16
genus
607
node719.members.0.js

206389
order
12213

75787
12213
family
617
node721.members.0.js

360
551759
genus

node723.members.0.js
360
species
551760

73029
877
genus
38
node724.members.0.js

259537
species
266
node725.members.0.js

302
node726.members.0.js
281362
species

271
node727.members.0.js
73030
species

827
genus
1914449

species
1565605
827
node729.members.0.js

291
146935
genus

291
node731.members.0.js
146938
species

genus
392735
346

392736
species
node733.members.0.js
346

1054211
genus
904

904
node735.members.0.js
748811
species

1526
378210
node736.members.0.js
209
genus

633
node737.members.0.js
species
1346290

338
node738.members.0.js
species
378211

346
node739.members.0.js
species
1119528

466
146936
genus

418702
species
node741.members.0.js
466

83766
genus
615

1860101
species
615
node743.members.0.js

1691980
species
349
node744.members.0.js

414
146937
node745.members.0.js
genus
1

146939
species
node746.members.0.js
413

12960
2382
genus
633
node747.members.0.js

1637998
species
node748.members.0.js
212

198107
species
node749.members.0.js
394

species
92003
525
node750.members.0.js

node751.members.0.js
618
748247
species

33057
2239
genus
1664
node752.members.0.js

76113
species
285
node753.members.0.js

164400
species
node754.members.0.js
290

206351
5155
70
order
node755.members.0.js

3567
1499392
node756.members.0.js
2044
family

650341
genus
240

356662
species
240
node758.members.0.js

535
genus
node759.members.0.js
572

568394
458
genus
241
node760.members.0.js

species
990370
node761.members.0.js
217

genus
551208
253
node762.members.0.js

family
830
node763.members.0.js
481
1518

482
688
genus
439
node764.members.0.js

species
487
249
node765.members.0.js

node766.members.0.js
209
species
1523421

species
1690485
322
node767.members.0.js

32003
4651
1
order
node768.members.0.js

node769.members.0.js
141
family
4650
206379

genus
230
node770.members.0.js
35798
2066

411
node771.members.0.js
species
1288494

1231
species
554
node772.members.0.js

species
35799
449
node773.members.0.js

58133
species
422
node774.members.0.js

node775.members.0.js
387
genus
2443
914

261292
species
node776.members.0.js
416

292
node777.members.0.js
species
44575

node778.members.0.js
241
153948
species

species
915
312
node779.members.0.js

296
node780.members.0.js
species
916

species
44574
node781.members.0.js
499

80840
238350
3579
order
node782.members.0.js

312
genus
node783.members.0.js
212743
604

node784.members.0.js
292
1736433
species

333
88
node785.members.0.js
genus
55

278
node786.members.0.js
species
34029

node787.members.0.js
139
genus
415
36862

node788.members.0.js
276
1547922
species

864051
species
364
node789.members.0.js

119060
190077
2068
family
node790.members.0.js

2064
106589
node791.members.0.js
1531
genus

68895
species
308
node792.members.0.js

node793.members.0.js
225
106590
species

genus
93217
node794.members.0.js
989

node795.members.0.js
793
genus
1519
48736

228
node796.members.0.js
species
329

species
305
node797.members.0.js
498

genus
44013
294
node798.members.0.js

131079
174133
44177
genus
node799.members.0.js

species
391597
node800.members.0.js
80867

species
1813033
node801.members.0.js
49089

344
240411
genus

356302
species
344
node803.members.0.js

1827195
genus
746
node804.members.0.js

node805.members.0.js
2795
genus
6125
32008

525
species group
node806.members.0.js
111527
1152

species
28450
627
node807.members.0.js

node808.members.0.js
species group
1025
1700
87882

node809.members.0.js
236
species
292

node810.members.0.js
207
87883
species

232
node811.members.0.js
species
60552

478
node812.members.0.js
species
101571

genus
1822464
node813.members.0.js
1795

1469502
species
node814.members.0.js
321

family
1069
node815.members.0.js
506
6512

303
290425
node816.members.0.js
81
genus

222
node817.members.0.js
310575
species

281
507
node818.members.0.js
60
genus

species
511
221
node819.members.0.js

genus
203697
229

species
203699
229
node821.members.0.js

genus
312063
335

335
node823.members.0.js
species
364039

1139
node824.members.0.js
genus
222

node825.members.0.js
genus
1273
2825
517

node826.members.0.js
260
94624
species

node827.members.0.js
667
species
520

518
species
node828.members.0.js
625

305976
genus
node829.members.0.js
331

node830.members.0.js
684
genus
1444
32012

species
339866
node831.members.0.js
243

554131
species
223
node832.members.0.js

node833.members.0.js
294
1158292
species

species
413882
node834.members.0.js
365

genus
391952
247

node836.members.0.js
247
species
391953

984
family
node837.members.0.js
75682
11288

genus
1479
node838.members.0.js
963
2390

709839
species
node839.members.0.js
433

1465823
species
478
node840.members.0.js

genus
198
node841.members.0.js
303379
416

node842.members.0.js
218
204773
species

481
genus
1344552

node844.members.0.js
481
1736487
species

149
genus
node845.members.0.js
202907
642

158899
species
268
node846.members.0.js

279058
species
225
node847.members.0.js

426
node848.members.0.js
genus
75654

genus
1192
node849.members.0.js
29580
1719

310
node850.members.0.js
species
1706231

375286
species
node851.members.0.js
217

149698
3981
genus
1951
node852.members.0.js

species
47229
599
node853.members.0.js

node854.members.0.js
235
species
1736272

node855.members.0.js
682
544911
species

node856.members.0.js
301
species
286638

1549812
species
node857.members.0.js
213

species
937450
node858.members.0.js
249

394
92793
node859.members.0.js
genus
155

node860.members.0.js
239
1538295
species

node861.members.0.js
100
family
312
995019

genus
40544
212
node862.members.0.js

316612
1500
893
genus
node863.members.0.js

1686064
species
node864.members.0.js
350

node865.members.0.js
257
1500261
species

114248
389
genus
124
node866.members.0.js

307486
species
node867.members.0.js
265

80864
19882
family
2784
node868.members.0.js

1059
47420
node869.members.0.js
270
genus

species
795665
node870.members.0.js
283

node871.members.0.js
250
species
1736490

node872.members.0.js
256
1437444
species

node873.members.0.js
360
species
1298913

node874.members.0.js
genus
129
464
28065

192843
species
node875.members.0.js
335

2370
12916
node876.members.0.js
2037
genus

47920
species
node877.members.0.js
333

genus
80865
node878.members.0.js
339

genus
196013
node879.members.0.js
253

324
232523
genus

species
80880
324
node881.members.0.js

495
node882.members.0.js
665874
genus

34072
3300
1289
genus
node883.members.0.js

node884.members.0.js
215
species
1736541

608
node885.members.0.js
207745
species

node886.members.0.js
1188
34073
species

287
genus
763439

species
1450524
node888.members.0.js
287

428
genus
node889.members.0.js
52972
2161

804
node890.members.0.js
species
1305734

216465
species
node891.members.0.js
219

500
node892.members.0.js
species
296591

species
1144318
210
node893.members.0.js

1332
281915
node894.members.0.js
genus
221

449
node895.members.0.js
667019
species

1714344
species
node896.members.0.js
662

219181
312
58
genus
node897.members.0.js

species
219182
node898.members.0.js
254

genus
565
node899.members.0.js
283
853

288
node900.members.0.js
285
species

659
335058
node901.members.0.js
genus
450

node902.members.0.js
209
1736464
species

1355
174951
node903.members.0.js
genus
37

395
node904.members.0.js
species
1736365

node905.members.0.js
923
94132
species

747294
genus
418
node906.members.0.js

201096
506
genus
284
node907.members.0.js

species
179636
node908.members.0.js
222

364316
genus
251
node909.members.0.js

28067
genus
node910.members.0.js
324

order
713636
3739

90627
3739
128
family
node912.members.0.js

genus
6
node913.members.0.js
96
1971

653
node914.members.0.js
370405
species

node915.members.0.js
1312
1796491
species

314343
779
1
genus
node916.members.0.js

species
63745
778
node917.members.0.js

1443590
861
76
genus
node918.members.0.js

node919.members.0.js
315
1188319
species

1485544
species
node920.members.0.js
470

species
1799662
1706
node921.members.0.js

1703389
species
node922.members.0.js
771

node923.members.0.js
217
1523428
species

order
1559979
4675

25
family
node925.members.0.js
1559980
4675

4650
935200
node926.members.0.js
514
genus

1803
node927.members.0.js
species
649841

node928.members.0.js
2333
species
1632857

species
1703390
node929.members.0.js
1975

82788
119069
order

206349
82788
1
family
node931.members.0.js

node932.members.0.js
7379
genus
82467
919

species
36861
node933.members.0.js
56689

species
931
18399
node934.members.0.js

203470
genus
320
node935.members.0.js

node936.members.0.js
14209
class
766915
1236

108451
1692040
order

108451
1692041
node938.members.0.js
508
family

92014
genus
1744881

1620215
species
92014
node940.members.0.js

15929
1692042
genus

species
1675686
node942.members.0.js
15929

86
genus
node943.members.0.js
349742
1109

node944.members.0.js
371
1543721
species

species
191960
652
node945.members.0.js

node946.members.0.js
795
species
1703403

441
node947.members.0.js
species
1703404

136
order
node948.members.0.js
72273
11269

node949.members.0.js
family
182
4085
135617

node950.members.0.js
95
genus
905
1021

1022
species
267
node951.members.0.js

species
422289
node952.members.0.js
312

node953.members.0.js
231
319938
species

609
90372
genus

species
1003181
609
node955.members.0.js

1589
1030
node956.members.0.js
257
genus

224
node957.members.0.js
125614
species

1031
species
node958.members.0.js
534

574
node959.members.0.js
species
525917

node960.members.0.js
327
44937
genus

473
40751
genus

40754
species
node962.members.0.js
473

135616
2284
329
family
node963.members.0.js

244
genus
node964.members.0.js
34067
458

1329899
species
node965.members.0.js
214

node966.members.0.js
754
genus
1002
40222

species
754476
248
node967.members.0.js

933
genus
node968.members.0.js
495

1703416
species
node969.members.0.js
4764

1703406
species
node970.members.0.js
1465

species
54398
node971.members.0.js
276

543646
72274
node972.members.0.js
order
571

family
1350
node973.members.0.js
135621
536681

534650
286
node974.members.0.js
135651
genus

1729723
species
node975.members.0.js
236

species
101564
268
node976.members.0.js

node977.members.0.js
760
291995
species

900
node978.members.0.js
440512
species

1487922
species
node979.members.0.js
224

species group
991
node980.members.0.js
136841
14868

996
node981.members.0.js
300
species

node982.members.0.js
460
species
53412

447
node983.members.0.js
43263
species

species
287
10871
node984.members.0.js

species subgroup
30
node985.members.0.js
1232139
1103

330
species
node986.members.0.js
1073

node987.members.0.js
3445
species
1439940

node988.members.0.js
433
553151
species

136843
2688
643
species group
node989.members.0.js

2045
node990.members.0.js
species
294

157784
species
269
node991.members.0.js

940
node992.members.0.js
1499686
species

1586417
species
node993.members.0.js
353

136845
2159
377
species group
node994.members.0.js

239
node995.members.0.js
species
47880

node996.members.0.js
1543
species
303

633150
species
node997.members.0.js
256

1203578
species
263
node998.members.0.js

5111
node999.members.0.js
species
1629711

node1000.members.0.js
217
species
686578

136849
5688
1826
species group
node1001.members.0.js

262
node1002.members.0.js
50340
species

546
node1003.members.0.js
species
251701

251695
species subgroup
1902

317
species
1902
node1005.members.0.js

species subgroup
246
node1006.members.0.js
251698
1152

29438
species
384
node1007.members.0.js

node1008.members.0.js
522
47877
species

218
node1009.members.0.js
species
554344

node1010.members.0.js
species group
91
477
136842

386
node1011.members.0.js
species
587753

356741
136846
node1012.members.0.js
species group
107822

14004
node1013.members.0.js
1619235
species

1400
node1014.members.0.js
74829
species

271420
species
23379
node1015.members.0.js

27094
species subgroup
node1016.members.0.js
578833
210136

node1017.members.0.js
11846
species
203192

171196
node1018.members.0.js
316
species

136844
species group
223

species
43306
node1020.members.0.js
223

species
574962
300
node1021.members.0.js

940
node1022.members.0.js
1729726
species

species
150396
node1023.members.0.js
1022

351
681
4
subfamily
node1024.members.0.js

genus
81
node1025.members.0.js
352
677

353
species
node1026.members.0.js
297

node1027.members.0.js
299
species
354

468
6394
299
family
node1028.members.0.js

469
5552
3583
genus
node1029.members.0.js

1619
909768
node1030.members.0.js
species group
844

470
species
775
node1031.members.0.js

species
40214
node1032.members.0.js
350

229
node1033.members.0.js
genus
475

genus
497
314
node1034.members.0.js

512
node1035.members.0.js
species
1248727

299
492233
genus

species
381306
node1037.members.0.js
299

order
59
node1038.members.0.js
1706369
3977

family
1706373
390

genus
48073
390
node1040.members.0.js

296
node1041.members.0.js
1706374
family

node1042.members.0.js
463
family
742
1706375

630749
genus
node1043.members.0.js
279

1715
1706371
node1044.members.0.js
family
468

615
node1045.members.0.js
genus
10

359337
genus
245
node1046.members.0.js

node1047.members.0.js
162
genus
387
2425

node1048.members.0.js
225
2426
species

1706372
family
775
node1049.members.0.js

234
node1050.members.0.js
83406
species

634
order
node1051.members.0.js
135622
6430

node1052.members.0.js
family
190
514
267889

324
node1053.members.0.js
genus
28228

792
267888
node1054.members.0.js
30
family

node1055.members.0.js
762
53246
genus

node1056.members.0.js
43
family
394
267893

351
node1057.members.0.js
135575
genus

family
1149
node1058.members.0.js
72275
3350

2201
node1059.members.0.js
2742
genus

267890
family
746

746
node1061.members.0.js
genus
22

135624
951
83
order
node1062.members.0.js

868
84642
node1063.members.0.js
family
178

genus
642
690
node1064.members.0.js

9544
135614
node1065.members.0.js
order
138

591
family
node1066.members.0.js
1775411
2501

227
886360
genus

227
node1068.members.0.js
species
540746

genus
1544982
219

1475481
species
219
node1070.members.0.js

220
node1071.members.0.js
genus
242605

node1072.members.0.js
851
genus
75309

393
node1073.members.0.js
231454
genus

32033
6905
family
714
node1074.members.0.js

node1075.members.0.js
1224
genus
1624
338

400
643453
node1076.members.0.js
species group
65

335
node1077.members.0.js
species
346

node1078.members.0.js
776
genus
490567

1752
node1079.members.0.js
genus
68

83618
genus
node1080.members.0.js
649

40323
938
581
genus
node1081.members.0.js

node1082.members.0.js
357
40324
species

genus
83614
452
node1083.members.0.js

488
genus
1608298

species
1076588
node1085.members.0.js
488

1799661
species
node1086.members.0.js
230

275
742030
order

family
742031
275

180541
genus
275
node1089.members.0.js

node1090.members.0.js
301
species
2340

135625
order
615

712
615
family
394
node1092.members.0.js

node1093.members.0.js
221
genus
724

order
8
node1094.members.0.js
135623
3253

node1095.members.0.js
386
family
3245
641

1207
genus
node1096.members.0.js
662
2302

species
666
node1097.members.0.js
461

node1098.members.0.js
128
species group
634
717610

species
670
251
node1099.members.0.js

255
node1100.members.0.js
species
663

node1101.members.0.js
557
657
genus

135618
8477
27
order
node1102.members.0.js

552
family
node1103.members.0.js
403
7721

73778
765
genus
5
node1104.members.0.js

node1105.members.0.js
760
species
73780

node1106.members.0.js
5
genus
608
160800

node1107.members.0.js
265
species
136992

338
node1108.members.0.js
105972
species

species
1495050
node1109.members.0.js
544

223
genus
1295378

223
node1111.members.0.js
species
1295382

1241
429
node1112.members.0.js
513
genus

node1113.members.0.js
443
173365
species

415
species
node1114.members.0.js
285

node1115.members.0.js
genus
466
699
39773

39775
species
node1116.members.0.js
233

genus
1410680
324

species
1410681
324
node1118.members.0.js

416
2005
genus
825
node1119.members.0.js

505
node1120.members.0.js
421
species

node1121.members.0.js
247
980561
species

702114
species
428
node1122.members.0.js

465
413
genus

414
species
node1124.members.0.js
465

genus
1221209
295

438855
species
node1126.members.0.js
295

729
1486721
node1127.members.0.js
8
family

356
244364
genus

244365
species
node1129.members.0.js
356

365
node1130.members.0.js
1798802
species

91347
order
7299

6327
543
node1132.members.0.js
3422
family

node1133.members.0.js
54
genus
456
547

402
354276
node1134.members.0.js
species group
98

550
species
node1135.members.0.js
304

787
570
node1136.members.0.js
genus
203

584
node1137.members.0.js
573
species

1115
561
node1138.members.0.js
130
genus

985
node1139.members.0.js
562
species

590
547
genus
9
node1140.members.0.js

node1141.members.0.js
538
species
28901

658
1903411
family

genus
613
312
node1143.members.0.js

node1144.members.0.js
346
genus
629

314
family
1903409

53335
genus
node1146.members.0.js
314

species
1703405
543
node1147.members.0.js

1356
1400857
node1148.members.0.js
family
25

1400859
genus
763

species
1400860
763
node1150.members.0.js

568
genus
221279

568
node1152.members.0.js
1400862
species

1703402
species
node1153.members.0.js
937

18538
135613
node1154.members.0.js
353
order

1007
family
255526

node1156.members.0.js
146
genus
1007
109262

927
species
281
node1157.members.0.js

1860122
species
580
node1158.members.0.js

1046
8342
family
377
node1159.members.0.js

156885
genus
366

366
node1161.members.0.js
species
80679

1813600
genus
1924

1924
node1163.members.0.js
1748243
species

node1164.members.0.js
333
genus
67575

node1165.members.0.js
genus
6
675
85079

1385625
species
316
node1166.members.0.js

1431688
species
353
node1167.members.0.js

3
genus
node1168.members.0.js
13724
378

73141
species
375
node1169.members.0.js

node1170.members.0.js
298
genus
85076

482
genus
53452

node1172.members.0.js
482
61598
species

309
genus
61593

309
node1174.members.0.js
species
631362

node1175.members.0.js
genus
83
787
1056

339
node1176.members.0.js
species
610332

node1177.members.0.js
365
species
244573

node1178.members.0.js
genus
191
1618
1227

258
node1179.members.0.js
species
473531

species
1229
node1180.members.0.js
354

815
node1181.members.0.js
species
133539

genus
85072
348

348
node1183.members.0.js
1049
species

447
57488
node1184.members.0.js
23
genus

1249627
species
node1185.members.0.js
213

211
node1186.members.0.js
210408
species

404
1738654
family

1738655
genus
404

404
node1189.members.0.js
1548547
species

72276
8432
465
family
node1190.members.0.js

133193
genus
321

species
351052
321
node1192.members.0.js

node1193.members.0.js
genus
65
796
1051

node1194.members.0.js
418
species
519989

313
node1195.members.0.js
species
1442136

275
154346
genus

node1197.members.0.js
275
154347
species

106633
4836
genus
1978
node1198.members.0.js

species
108010
node1199.members.0.js
223

species
1033854
1409
node1200.members.0.js

node1201.members.0.js
936
species
243063

node1202.members.0.js
290
186931
species

genus
85108
node1203.members.0.js
252

1765964
genus
826

826
node1205.members.0.js
160660
species

310
2368
node1206.members.0.js
3
genus

2369
species
node1207.members.0.js
307

genus
35796
351

351
node1209.members.0.js
species
35797

order
10
node1210.members.0.js
118969
9727

8499
444
node1211.members.0.js
family
663

7836
445
node1212.members.0.js
genus
4812

29423
species
255
node1213.members.0.js

589
node1214.members.0.js
species
28082

species
446
node1215.members.0.js
1458

species
66969
node1216.members.0.js
722

118968
1218
family
338
node1217.members.0.js

568
node1218.members.0.js
1590043
species

776
genus
312
node1219.members.0.js

8245
135619
node1220.members.0.js
order
124

node1221.members.0.js
family
1391
2808
135620

node1222.members.0.js
354
genus
267849

node1223.members.0.js
390
genus
28253

genus
48075
673
node1224.members.0.js

family
144
node1225.members.0.js
224379
858

node1226.members.0.js
373
158481
genus

341
node1227.members.0.js
genus
305899

284
191033
family

node1229.members.0.js
284
genus
141450

node1230.members.0.js
family
527
2665
28256

2745
genus
node1231.members.0.js
2138

255527
family
node1232.members.0.js
305

196
family
node1233.members.0.js
224372
1201

1005
node1234.members.0.js
59753
genus

node1235.members.0.js
211
order
3023
1775403

node1236.members.0.js
family
68
2812
568386

genus
469322
363

species
465721
node1238.members.0.js
363

568
genus
node1239.members.0.js
413435
838

413479
species
node1240.members.0.js
270

324
genus
1274363

node1242.members.0.js
324
species
616992

243627
genus
412

species
243629
node1244.members.0.js
412

807
64001
node1245.members.0.js
genus
13

418856
species
node1246.members.0.js
412

382
node1247.members.0.js
species
64002

subphylum
7
node1248.members.0.js
68525
35121

837
29547
node1249.members.0.js
138
class

699
213849
node1250.members.0.js
order
19

355
node1251.members.0.js
family
72294

node1252.members.0.js
118
family
325
72293

genus
209
207
node1253.members.0.js

node1254.members.0.js
class
1479
34277
28221

species
1640514
388
node1255.members.0.js

genus
1551504
1248

1248
node1257.members.0.js
673862
species

node1258.members.0.js
2
order
1780
213462

213465
family
249

29526
genus
node1260.members.0.js
249

1276
213468
node1261.members.0.js
397
family

277
2357
genus

277
node1263.members.0.js
species
2358

genus
182623
602
node1264.members.0.js

513556
family
253

253
513557
genus

node1267.members.0.js
253
328301
species

node1268.members.0.js
14
order
2779
213115

224380
family
290

node1270.members.0.js
290
genus
66848

213116
268
family
47
node1271.members.0.js

node1272.members.0.js
221
genus
898

194924
1795
154
family
node1273.members.0.js

genus
872
1641
node1274.members.0.js

family
213117
node1275.members.0.js
412

node1276.members.0.js
7799
1797818
species

4426
213118
node1277.members.0.js
264
order

3019
213119
node1278.members.0.js
family
1328

node1279.members.0.js
333
genus
1303912

node1280.members.0.js
309
2299
genus

215
418098
genus

node1282.members.0.js
215
species
418099

node1283.members.0.js
118
genus
457
2295

species
201089
339
node1284.members.0.js

377
node1285.members.0.js
genus
896

1143
213121
node1286.members.0.js
537
family

node1287.members.0.js
606
893
genus

422
213481
node1288.members.0.js
order
137

285
213483
family

285
node1290.members.0.js
genus
958

244
node1291.members.0.js
species
88274

species
262489
node1292.members.0.js
273

node1293.members.0.js
order
53
4901
69541

node1294.members.0.js
family
10
3290
213422

2676
28231
node1295.members.0.js
genus
591

225
node1296.members.0.js
species
313985

247
node1297.members.0.js
1266765
species

351604
species
node1298.members.0.js
368

node1299.members.0.js
267
28232
species

node1300.members.0.js
285
443143
species

node1301.members.0.js
212
1203471
species

345632
species
224
node1302.members.0.js

node1303.members.0.js
257
species
35554

271087
genus
209

209
node1305.members.0.js
225196
species

node1306.members.0.js
395
genus
392332

family
10
node1307.members.0.js
213421
1558

node1308.members.0.js
382
genus
592
18

species
407188
node1309.members.0.js
210

890
956
genus
155
node1310.members.0.js

node1311.members.0.js
272
species
1232410

species
1603606
node1312.members.0.js
265

264
node1313.members.0.js
1823759
species

node1314.members.0.js
328
1703399
species

325
order
453227

family
453228
node1316.members.0.js
325

7588
29
node1317.members.0.js
order
432

node1318.members.0.js
148
suborder
1531
224462

family
224464
1383

genus
162027
1383

species
80816
1383
node1321.members.0.js

215
215802
genus

215803
species
node1323.members.0.js
215

suborder
17
node1324.members.0.js
80812
2246

226
family
1524216

genus
1524217
226

species
1391654
node1327.members.0.js
226

14
family
node1328.members.0.js
49
1455

genus
39643
1035

species
56
1035
node1330.members.0.js

406
node1331.members.0.js
50
genus

548
family
1055686

548
1055688
genus

node1334.members.0.js
548
species
927083

3164
80811
node1335.members.0.js
suborder
229

family
196
node1336.members.0.js
31
834

416
genus
node1337.members.0.js
32
638

species
33
node1338.members.0.js
222

769
family
1524215

769
161492
node1340.members.0.js
genus
467

302
node1341.members.0.js
404589
species

node1342.members.0.js
family
117
1332
39

240
40
genus

species
41
node1344.members.0.js
240

297
224455
genus

node1346.members.0.js
297
394096
species

218
genus
47

48
species
218
node1348.members.0.js

42
460
genus
5
node1349.members.0.js

83451
species
220
node1350.members.0.js

235
node1351.members.0.js
species
43

genus
1125862
297

species
1035120
297
node1353.members.0.js

species
1665206
node1354.members.0.js
392

389
580370
node1355.members.0.js
143
class

246
order
580371

246
580372
family

246
377315
genus

246
node1359.members.0.js
314344
species

1807140
class
15051

15051
225057
node1361.members.0.js
order
5559

1377
node1362.members.0.js
species
1703385

1390
family
225058

genus
489
node1364.members.0.js
119977
1390

node1365.members.0.js
248
species
920

245
node1366.members.0.js
species
33059

408
node1367.members.0.js
species
160808

node1368.members.0.js
3093
species
1703384

1358
225059
family

genus
119979
1358

node1371.members.0.js
1358
929
species

2274
node1372.members.0.js
1703383
species

245224
28211
node1373.members.0.js
6046
class

204441
18093
order
188
node1374.members.0.js

1380394
species
857
node1375.members.0.js

41295
11564
family
1203
node1376.members.0.js

node1377.members.0.js
615
genus
1671
191

species
34010
node1378.members.0.js
366

193
species
241
node1379.members.0.js

192
species
449
node1380.members.0.js

822
1121829
node1381.members.0.js
genus
268

species
1121830
280
node1382.members.0.js

1121832
species
node1383.members.0.js
274

genus
85274
747

1087
species
747
node1385.members.0.js

genus
85265
node1386.members.0.js
216

genus
171673
640

171674
species
node1388.members.0.js
640

1081
474
genus
162
node1389.members.0.js

node1390.members.0.js
312
34018
species

253
414051
genus

253
node1392.members.0.js
species
859058

genus
390876
283

node1394.members.0.js
283
390877
species

303
1182780
genus

303
node1396.members.0.js
species
1288970

171436
genus
315

315
node1398.members.0.js
species
171437

423
1146845
genus

species
1380391
node1400.members.0.js
423

node1401.members.0.js
608
genus
168934

659693
766
41
genus
node1402.members.0.js

node1403.members.0.js
348
526216
species

node1404.members.0.js
377
580166
species

209
526215
genus

node1406.members.0.js
209
species
455360

836
204447
node1407.members.0.js
genus
69

367
node1408.members.0.js
species
913326

species
393310
node1409.members.0.js
400

1382
13134
node1410.members.0.js
856
genus

55518
species
node1411.members.0.js
311

215
node1412.members.0.js
1437059
species

genus
1612157
413

1084
species
node1414.members.0.js
413

family
1236
node1415.members.0.js
433
2613

400
node1416.members.0.js
434
genus

genus
125216
node1417.members.0.js
397

522
genus
node1418.members.0.js
343

genus
365532
node1419.members.0.js
237

2586
genus
445219

species
445220
2586
node1421.members.0.js

285
genus
457933

species
457934
285
node1423.members.0.js

150696
356
node1424.members.0.js
order
2847

41294
82010
2499
family
node1425.members.0.js

genus
1395974
295

295
node1427.members.0.js
species
1792031

genus
468
node1428.members.0.js
1073
2331

1076
species
1863
node1429.members.0.js

node1430.members.0.js
1044
85413
genus

942
genus
node1431.members.0.js
1033
2432

species
56946
305
node1432.members.0.js

300
node1433.members.0.js
species
211460

species
1035
node1434.members.0.js
258

species
1429916
node1435.members.0.js
329

species
1736436
298
node1436.members.0.js

37002
genus
node1437.members.0.js
374
71412

species
1768760
node1438.members.0.js
310

node1439.members.0.js
20110
1736363
species

node1440.members.0.js
2855
375
species

217
node1441.members.0.js
318996
species

266
node1442.members.0.js
species
1298867

node1443.members.0.js
335
1497613
species

1761772
species
node1444.members.0.js
281

43992
species
node1445.members.0.js
425

682
node1446.members.0.js
108015
species

376
species
339
node1447.members.0.js

node1448.members.0.js
269
1274631
species

node1449.members.0.js
270
species
551947

1355477
species
969
node1450.members.0.js

538
node1451.members.0.js
288000
species

1230476
species
node1452.members.0.js
207

254
node1453.members.0.js
196490
species

species
29448
973
node1454.members.0.js

species
113395
282
node1455.members.0.js

species
189753
264
node1456.members.0.js

1380355
species
node1457.members.0.js
305

species
1518501
418
node1458.members.0.js

1755647
species
node1459.members.0.js
207

node1460.members.0.js
2081
1768753
species

665
node1461.members.0.js
1126627
species

1040989
species
node1462.members.0.js
341

node1463.members.0.js
234
species
335659

1128178
species
313
node1464.members.0.js

genus
40136
367

40137
species
node1466.members.0.js
367

genus
1649510
717

species
1333996
node1468.members.0.js
717

81
genus
node1469.members.0.js
911
913

213
node1470.members.0.js
913
species

node1471.members.0.js
383
species
912

236
node1472.members.0.js
species
314253

node1473.members.0.js
family
430
1923
335928

genus
556257
824

1736466
species
824
node1475.members.0.js

228
node1476.members.0.js
6
genus

279
441
genus
151
node1477.members.0.js

node1478.members.0.js
290
species
280

node1479.members.0.js
family
964
5471
82115

2887
379
node1480.members.0.js
2352
genus

species
384
535
node1481.members.0.js

106591
genus
node1482.members.0.js
389

28105
752
genus
507
node1483.members.0.js

species
380
node1484.members.0.js
245

genus
357
479
node1485.members.0.js

1484898
genus
702

node1487.members.0.js
702
1384459
species

1277
119043
node1488.members.0.js
517
family

genus
1742977
266

266
node1490.members.0.js
1333998
species

281
256616
genus

281
node1492.members.0.js
species
256618

1358438
genus
213

213
node1494.members.0.js
species
1358440

472
118882
node1495.members.0.js
family
247

node1496.members.0.js
225
528
genus

688
family
node1497.members.0.js
45401
7821

238
node1498.members.0.js
genus
1068

46913
genus
1033
node1499.members.0.js

427
119044
genus

427
node1501.members.0.js
species
1608628

1796
29407
genus

674703
species
1796
node1503.members.0.js

node1504.members.0.js
618
genus
3639
81

species
121290
1463
node1505.members.0.js

species
48292
node1506.members.0.js
750

293
node1507.members.0.js
species
53399

species
1427356
299
node1508.members.0.js

1163419
species
node1509.members.0.js
216

45404
1012
396
family
node1510.members.0.js

genus
28209
node1511.members.0.js
368

genus
184923
248
node1512.members.0.js

1862950
species
390
node1513.members.0.js

node1514.members.0.js
family
536
1469
31993

345
node1515.members.0.js
genus
425

node1516.members.0.js
588
133
genus

119045
19705
121
family
node1517.members.0.js

1094
186650
node1518.members.0.js
723
genus

371
node1519.members.0.js
species
1033741

407
18490
13279
genus
node1520.members.0.js

216
node1521.members.0.js
species
270351

1736246
species
node1522.members.0.js
1377

2576
578822
species group

species
408
node1524.members.0.js
2576

node1525.members.0.js
226
species
925818

816
node1526.members.0.js
species
223967

24331
69277
node1527.members.0.js
614
family

node1528.members.0.js
genus
2765
22700
68287

species
381
234
node1529.members.0.js

node1530.members.0.js
281
species
475067

1574409
species
node1531.members.0.js
19420

305
node1532.members.0.js
genus
274591

node1533.members.0.js
257
genus
449972

245876
genus
455
node1534.members.0.js

401
family
node1535.members.0.js
255475
952

genus
414371
551
node1536.members.0.js

1411123
species
node1537.members.0.js
314

node1538.members.0.js
420
species
331869

204458
order
5077

node1540.members.0.js
301
family
5077
76892

76890
genus
node1541.members.0.js
339

genus
134
node1542.members.0.js
20
740

386
node1543.members.0.js
284016
species

1736591
species
node1544.members.0.js
220

species
1759059
node1545.members.0.js
289

41275
2339
2122
genus
node1546.members.0.js

1736443
species
217
node1547.members.0.js

genus
75
1069
node1548.members.0.js

node1549.members.0.js
44
order
10290
204455

2211
69657
node1550.members.0.js
family
1068

1143
85
node1551.members.0.js
genus
909

74319
species
node1552.members.0.js
234

node1553.members.0.js
family
5204
8035
31989

402
node1554.members.0.js
genus
478070

1060
genus
node1555.members.0.js
458

node1556.members.0.js
480
genus
265

381
node1557.members.0.js
genus
74030

536
34008
node1558.members.0.js
88
genus

species
1187851
node1559.members.0.js
448

235
node1560.members.0.js
295418
genus

genus
97050
node1561.members.0.js
339

597358
order
1288

1288
family
597359

1288
454159
node1564.members.0.js
genus
530

454162
species
531
node1565.members.0.js

species
1549748
227
node1566.members.0.js

node1567.members.0.js
366
species
1734406

genus
489140
208

species
404900
208
node1569.members.0.js

662
node1570.members.0.js
766
order

230
node1571.members.0.js
species
1492281

node1572.members.0.js
order
2099
51211
204457

335929
11884
family
1251
node1573.members.0.js

361177
3564
genus
721
node1574.members.0.js

species
1736584
node1575.members.0.js
370

node1576.members.0.js
969
species
292222

543877
species
node1577.members.0.js
540

620
node1578.members.0.js
species
692370

species
361183
node1579.members.0.js
344

2947
genus
node1580.members.0.js
1041
5856

node1581.members.0.js
382
species
39960

node1582.members.0.js
652
species
1822240

node1583.members.0.js
302
species
291985

499656
species
node1584.members.0.js
475

1860125
species
node1585.members.0.js
771

161528
species
327
node1586.members.0.js

genus
1111
node1587.members.0.js
1213

41297
36603
family
2908
node1588.members.0.js

node1589.members.0.js
2600
genus
2940
165695

node1590.members.0.js
340
13690
species

node1591.members.0.js
genus
5656
9224
13687

node1592.members.0.js
259
397260
species

species
160791
node1593.members.0.js
313

species
392409
node1594.members.0.js
520

species
745310
node1595.members.0.js
396

node1596.members.0.js
515
species
407019

node1597.members.0.js
365
529705
species

1200
node1598.members.0.js
species
1380389

495
node1599.members.0.js
genus
72173

genus
7174
node1600.members.0.js
165697
17103

node1601.members.0.js
281
species
267128

216
node1602.members.0.js
species
33052

319
node1603.members.0.js
species
262667

437
node1604.members.0.js
1759073
species

117207
species
node1605.members.0.js
898

node1606.members.0.js
329
species
292913

species
1515612
node1607.members.0.js
579

1502850
species
node1608.members.0.js
378

1120705
species
node1609.members.0.js
4771

280
node1610.members.0.js
1736474
species

523
node1611.members.0.js
1759072
species

918
node1612.members.0.js
33050
species

165696
3365
genus
3129
node1613.members.0.js

node1614.members.0.js
236
702113
species

node1615.members.0.js
genus
185
568
150203

383
node1616.members.0.js
1419876
species

node1617.members.0.js
281
species
1734408

species
1734407
344
node1618.members.0.js

637
order
255473

637
255474
family

208215
637
13
genus
node1621.members.0.js

391
node1622.members.0.js
1247963
species

species
208216
node1623.members.0.js
233

node1624.members.0.js
196
phylum
5461
204428

node1625.members.0.js
12
class
5265
204429

node1626.members.0.js
620
order
5253
51291

412447
625
5
family
node1627.members.0.js

genus
483422
251

species
483423
node1629.members.0.js
251

genus
340070
369

369
node1631.members.0.js
species
340071

809
786
family
10
node1632.members.0.js

393
genus
1851152

node1634.members.0.js
393
species
1444712

810
genus
node1635.members.0.js
383

292
family
92714

292
71666
genus

species
71667
node1638.members.0.js
292

53
family
node1639.members.0.js
92713
1696

83551
genus
501

83552
species
501
node1641.members.0.js

node1642.members.0.js
385
112987
genus

1314958
species
node1643.members.0.js
329

428
node1644.members.0.js
282132
genus

family
92712
1234

34093
genus
1234

species
83561
node1647.members.0.js
1234

1239
21240
987
phylum
node1648.members.0.js

909932
818
303
class
node1649.members.0.js

node1650.members.0.js
order
1
515
909929

1843490
family
284
node1651.members.0.js

family
1843491
node1652.members.0.js
230

10468
91061
node1653.members.0.js
51
class

1385
7694
862
order
node1654.members.0.js

node1655.members.0.js
941
family
3061
186817

genus
129337
322
node1656.members.0.js

genus
1386
node1657.members.0.js
1798

90964
330
49
family
node1658.members.0.js

node1659.members.0.js
281
1279
genus

family
186818
274
node1660.members.0.js

186822
2595
family
437
node1661.members.0.js

node1662.members.0.js
1876
44249
genus

55080
genus
node1663.members.0.js
282

node1664.members.0.js
family
202
572
186823

29330
genus
node1665.members.0.js
370

node1666.members.0.js
417
order
2723
186826

1300
1856
family
25
node1667.members.0.js

1831
1301
node1668.members.0.js
258
genus

1313
species
node1669.members.0.js
1265

671232
308
4
species group
node1670.members.0.js

304
node1671.members.0.js
species
1328

450
33958
node1672.members.0.js
14
family

node1673.members.0.js
436
1578
genus

186801
8967
212
class
node1674.members.0.js

157
order
node1675.members.0.js
68295
1108

711
186814
node1676.members.0.js
family
488

44260
genus
223
node1677.members.0.js

family
543371
240
node1678.members.0.js

order
1239
node1679.members.0.js
186802
7398

family
186803
node1680.members.0.js
862

539000
376
family
154
node1681.members.0.js

node1682.members.0.js
222
genus
73918

186806
family
node1683.members.0.js
244

31979
1549
395
family
node1684.members.0.js

genus
1485
1154
node1685.members.0.js

1942
186807
node1686.members.0.js
1102
family

node1687.members.0.js
246
79206
genus

genus
1562
node1688.members.0.js
594

475
family
node1689.members.0.js
541000
731

256
node1690.members.0.js
1508657
genus

family
186804
node1691.members.0.js
211

244
node1692.members.0.js
68298
family

53433
order
node1693.members.0.js
249

439
phylum
200930

68337
class
439

191393
order
439

node1697.members.0.js
439
family
191394

227
phylum
node1698.members.0.js
40117
57704

203693
57228
class
36
node1699.members.0.js

55998
189778
order

family
13
node1701.members.0.js
189779
55998

995729
genus
1882

species
1609970
node1703.members.0.js
1882

179
666
416
genus
node1704.members.0.js

261386
species group
node1705.members.0.js
250

node1706.members.0.js
382
genus
28261

genus
194
node1707.members.0.js
40118
421

29290
species
227
node1708.members.0.js

52634
1234
node1709.members.0.js
4142
genus

330214
species
node1710.members.0.js
3117

node1711.members.0.js
3536
species
1799664

42253
species
node1712.members.0.js
25880

node1713.members.0.js
5181
species
1742973

1742972
species
3799
node1714.members.0.js

1715989
species
node1715.members.0.js
4170

species
1617410
2809
node1716.members.0.js

343
node1717.members.0.js
species
1704023

species
1704025
270
node1718.members.0.js

node1719.members.0.js
292
species
1704024

289
node1720.members.0.js
species
1704026

249
node1721.members.0.js
species
1748249

species
198431
node1722.members.0.js
228

2157
9865
superkingdom
842
node1723.members.0.js

28890
2934
phylum
680
node1724.members.0.js

1400
224756
node1725.members.0.js
123
class

node1726.members.0.js
363
order
2191

node1727.members.0.js
order
160
914
94695

1392996
family
226

226
node1729.members.0.js
genus
1392997

156
family
node1730.members.0.js
2206
528

genus
2207
372
node1731.members.0.js

183967
291
class
83
node1732.members.0.js

2301
order
node1733.members.0.js
208

node1734.members.0.js
class
348
563
183963

node1735.members.0.js
215
2235
order

6089
651137
node1736.members.0.js
phylum
871

5218
1643678
class

5218
order
1033996

5218
1033997
node1739.members.0.js
69
family

node1740.members.0.js
317
genus
5149
497726

399
node1741.members.0.js
1034015
species

node1742.members.0.js
327
species
1459637

node1743.members.0.js
4106
species
497727

9488
2759
node1744.members.0.js
superkingdom
1308

33208
2820
kingdom
433
node1745.members.0.js

node1746.members.0.js
240
phylum
630
6656

390
superclass
6960

class
50557
390

node1749.members.0.js
subclass
72
390
33340

318
node1750.members.0.js
infraclass
33392

7711
1404
phylum
49
node1751.members.0.js

node1752.members.0.js
212
subphylum
1355
89593

835
40674
node1753.members.0.js
class
82

382
node1754.members.0.js
314145
superorder

371
node1755.members.0.js
superorder
314146

308
superclass
7898

308
class
186623

41665
308
5
subclass
node1758.members.0.js

303
node1759.members.0.js
infraclass
32443

353
6231
node1760.members.0.js
phylum
63

290
119089
node1761.members.0.js
44
class

6236
246
order
33
node1762.members.0.js

213
55879
superfamily

213
6243
family

55885
subfamily
213

6237
genus
node1766.members.0.js
213

node1767.members.0.js
7
kingdom
2288
33090

phylum
455
node1768.members.0.js
35493
2002

node1769.members.0.js
order
2
647
3524

645
1804623
node1770.members.0.js
family
130

1804621
subfamily
515

515
3554
genus

node1773.members.0.js
515
species
161934

4447
308
class
15
node1774.members.0.js

node1775.members.0.js
subclass
45
293
4734

248
38820
node1776.members.0.js
15
order

node1777.members.0.js
233
4479
family

subclass
71275
node1778.members.0.js
592

279
node1779.members.0.js
3041
phylum

node1780.members.0.js
246
kingdom
3072
4751

8
subkingdom
node1781.members.0.js
451864
2826

node1782.members.0.js
145
phylum
2276
4890

2131
147538
node1783.members.0.js
153
subphylum

1048
147545
node1784.members.0.js
class
137

451871
911
129
subclass
node1785.members.0.js

node1786.members.0.js
order
66
782
5042

716
1131492
node1787.members.0.js
100
family

node1788.members.0.js
616
genus
5052

class
73
node1789.members.0.js
147541
318

node1790.members.0.js
subclass
19
245
451868

226
node1791.members.0.js
order
92860

class
137
node1792.members.0.js
147550
612

node1793.members.0.js
75
subclass
475
222543

400
node1794.members.0.js
order
5125

node1795.members.0.js
phylum
169
542
5204

5302
373
subphylum
88
node1796.members.0.js

155619
class
node1797.members.0.js
285

species
155900
259
node1798.members.0.js

10239
530
285
superkingdom
node1799.members.0.js

245
node1800.members.0.js
28883
order
